# RNF213 Is an Interferon‐Stimulated Gene That Targets Influenza A Virus NP and Activates MDA5 to Restrict Infection

**DOI:** 10.1002/advs.202511012

**Published:** 2026-03-18

**Authors:** Haoning Li, Yuying Zhang, Jingjing Wang, Qianqian Zhang, Hailiang Sun, Fanhua Wei

**Affiliations:** ^1^ College of Animal Science and Technology Ningxia University Yinchuan China; ^2^ School of Biological Science and Technology University of Jinan Jinan China; ^3^ College of Veterinary Medicine South China Agricultural University Guangzhou China

**Keywords:** influenza virus, interferon, interferon‐stimulated genes, MDA5, RNF213

## Abstract

Influenza A virus (IAV) infection induces a type I interferon (IFN) response in host cells, which exerts antiviral effects by upregulating interferon‐stimulated genes (ISGs). However, the ISG response to IAV remains incompletely understood. Here, we systematically identify antiviral ISGs in A549 cells following type I and type II IFN treatment. We demonstrate that ring finger protein 213 (RNF213) strongly inhibits IAV replication in vitro. Notably, RNF213 knockout (KO) mice exhibit heightened susceptibility to IAV infection, with exacerbated disease severity. Mechanistically, RNF213, which is induced by both IFN signaling and IAV infection, amplifies IFN production by enhancing melanoma‐differentiation‐associated gene 5 (MDA5) signaling. We further show that RNF213 interacts with MDA5 via its AAA+ ATPase and E3 ligase domains, promoting K63‐linked polyubiquitination of MDA5 at Lys137 and Lys743. Additionally, RNF213 mediates K48‐linked polyubiquitination of viral nucleoprotein (NP), targeting it for proteasomal degradation. Our findings identify RNF213 as a critical antiviral ISG that bridges innate immune activation and direct viral restriction. Our study reveals a dual mechanism of RNF213 in antiviral immunity, highlighting its potential as a therapeutic target against influenza.

## Graphical abstract

RNF213 is characterized as a dual‐functional antiviral effector. It directly mediates the degradation of the influenza A virus nucleoprotein (NP) while simultaneously activating the MDA5‐mediated innate immune signaling pathway. This coordinated response establishes a powerful host defense system against viral infection.

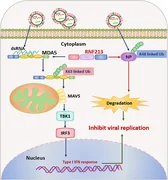

## 1 Introduction

Influenza viruses are enveloped, single‐stranded, negative‐sense RNA viruses that belong to the *Orthomyxoviridae* family. They are categorized based on the matrix protein (M) and NP into four types: A, B, C, and D [1]. Among these, IAV is the most virulent and widespread, causing significant economic losses in the livestock and poultry industries. Furthermore, IAV has the ability to cross species barriers, infecting mammals and leading to fatalities. Globally, influenza virus infections result in an estimated 290 000 to 650 000 deaths annually, representing a substantial threat to public health safety. Based on the variations in hemagglutinin (HA) and neuraminidase (NA) proteins, IAV is further classified into subtypes. To date, H17 and H18 are from bat IAV [2], and H19 does not bind the canonical receptor sialic acid and exhibits a limited host range and zoonotic potential [3].

Current strategies to combat IAV infection primarily rely on vaccines and antiviral medications. However, the rapid evolutionary nature of IAV enables it to circumvent vaccine‐induced immunity and develop resistance to antiviral drugs. Emerging virus subtypes often exhibit reduced binding affinity to neutralizing antibodies generated by vaccines, leading to mismatches or diminished vaccine effectiveness. Additionally, certain antiviral‐resistant variants have become prevalent in circulating strains. For instance, mutations such as H274Y in the NA protein and L26F, V27A, and S31N in the M2 protein have rendered antiviral drugs ineffective [4, 5, 6, 7]. Despite the genetic diversity and evolution of IAV subtypes, their capacity to infect and cause disease in hosts remains fundamentally dependent on virus‐host interactions. Consequently, further exploration of host proteins that influence the viral life cycle and pathogenicity, coupled with a more profound understanding of the natural immune mechanisms governing virus‐host dynamics, is imperative. Such insights can significantly advance IAV prevention strategies and the development of novel antiviral therapeutics.

The innate immune system provides the first line of defense against influenza virus infection. During IAV infection, the innate immune system initiates with the recognition of viral pathogen‐associated molecular patterns (PAMPs) by the host pattern recognition receptors (PRRs), including Toll‐like receptors (TLRs), retinoic acid‐inducible gene‐I (RIG‐I)‐like receptors (RLRs), and NOD‐like receptors (NLRs) [8, 9, 10], which activate a series of subsequent signaling pathways, prompting the production of proinflammatory cytokines and IFNs. Type I IFN (IFN‐α/β) is widely recognized as the primary antiviral interferon, while type II IFN (IFN‐γ) also exhibits significant antiviral capabilities [11]. For instance, early administration of IFN‐γ following influenza virus infection has been shown to protect mice from lethal outcomes [12]. Subsequently, IFNs bind to their respective receptors, such as the heterodimeric IFNAR1/IFNAR2 complex for IFN‐α/β and the IFNGR1/IFNGR2 dimer for IFN‐γ, activating the Janus kinase‐signal transducer and activator of transcription (JAK‐STAT) pathway. This process leads to the transcriptional upregulation of hundreds of ISGs. Each type of IFN has been shown to induce a distinct yet partially overlapping set of ISGs [13]. Ideally, ISGs function to suppress viral replication and safeguard cells and tissues from infection‐related damage [14]. Consequently, identifying and understanding the roles of specific ISGs is critical for developing effective clinical interventions in influenza virus infection and immunity.

RNF213, also known as Mysterin, is a member of the RNF family and has a molecular weight of 591 kDa. RNF213 was initially identified as a susceptibility gene for moyamoya disease (MMD) [15, 16]. Subsequent studies have revealed the critical role of RNF213 in combating viral, bacterial, parasitic, and chlamydial infections. During duck Tembusu virus (DTMUV) infection, upregulation of RNF213 induces K48‐linked ubiquitination of suppressor of cytokine signaling 1 (SOCS1), leading to its proteasomal degradation and ultimately inhibiting DTMUV infection [17]. Conversely, deletion of the RNF213 gene has been observed to increase susceptibility to Rift Valley fever virus (RVFV) infection, while overexpression of RNF213 in mice has been shown to enhance resistance to RVFV and significantly alleviate post‐infection symptoms [18]. Moreover, RNF213 restricts *Listeria monocytogenes* [19] and cytosolic *Salmonella* [20] infections and results in reduced infections of herpes simplex virus 1 (HSV‐1), human respiratory syncytial virus (RSV), and coxsackievirus B3 (CVB3) [19]. RNF213 also inhibits the de novo infection and lytic reactivation of γ‐herpesvirus by degrading the replication and transcription activator (RTA) of both Kaposi's sarcoma‐associated herpesvirus (KSHV) and mouse gammaherpesvirus 68 (MHV68) through the proteasome‐dependent pathway [21]. In addition, RNF213 recognizes and ubiquitinates modified lipopolysaccharide (LPS) of Gram‐negative bacteria as well as the Parasitophorous vacuole membrane (PVM) structure of Toxoplasma gondii to inhibit *Salmonella* and *Toxoplasma gondii* infections [20, 22]. These diverse findings suggest that RNF213 may employ multiple strategies—ranging from modulating host immune signaling to directly targeting pathogen components—to execute its antimicrobial functions, highlighting its potential as a versatile effector in host defense.

Given its established role as an interferon‐stimulated gene and its broad‐spectrum antimicrobial activity, we hypothesized that RNF213 might function as a key antiviral ISG during influenza A virus infection, potentially through a multifaceted mechanism. Here, we identified RNF213 as an antiviral ISG induced by both the type I and type II IFNs, which can effectively inhibit IAV replication in vitro and in vivo. We have also demonstrated the molecular mechanism of RNF213 in positively regulating the MDA5‐mediated IFN‐β production. We further demonstrated that RNF213 interacted with MDA5 via its AAA+ ATPase and E3 ligase domains and promoted K63‐linked polyubiquitination of MDA5 at Lys137 and Lys743. We also found that RNF213 interacted with viral NP and degraded NP through K48‐linked ubiquitination. Our findings establish RNF213 as a multifunctional antiviral ISG that bridges innate immune activation with direct viral restriction, providing new insights into host defense mechanisms against influenza.

## 2 Materials and Methods

### 2.1 Ethics Statement

All animal experiments were performed in accordance with the regulations and guidelines established by the committee and international standards for animal welfare and were approved by the Experimental Animal Ethics Committee of South China Agricultural University (Identification code: 2023f200).

### 2.2 Viruses and Cells

Influenza A/Puerto Rico/8/1934 (PR8; H1N1) strain was maintained in our laboratory. The virus was propagated in MDCK cells or 10‐day‐old embryonated eggs, and its infectivity was quantified by the 50% tissue culture infectious dose (TCID_50_) assay of MDCK cultures. MDA5 KO (MDA5^−/−^ HEK293T) cell lines were generously given by Dr. Xin Cao from Jilin Agricultural University. The methods of cell culture, viral titration, and mouse infection were performed as previously described [23]. The human lung adenocarcinoma epithelial cells (A549), human embryonic kidney cells (HEK293T), and Madin‐Darby canine kidney cells (MDCK) were cultured at 37°C under 5% CO_2_ in DMEM supplemented with 10% (v/v) heat‐inactivated fetal bovine serum (FBS; Gibco), 100 U/mL penicillin, and 100 µg/mL streptomycin.

### 2.3 Plasmid Construction and Transfection

Expression plasmids for RIG‐I, MDA5, MAVS, TBK1, IRF3, HA‐Ub WT, and HA‐Ub mutants were generated and maintained in our lab as described before [23, 24]. Full‐length RNF213 and mutants of RNF213 were generated using PRK5 containing different tags or pCDNA3.1 vectors. Mutants of RNF213 and some mutants expressing plasmids of MDA5 were constructed using the KOD‐Plus‐Mutagenesis kit (Toyobo). Expression plasmids for Flag‐MDA5, Flag‐MDA5‐K23R, Flag‐MDA5‐K43R, Flag‐MDA5‐K68R, Flag‐MDA5‐K128R, Flag‐MDA5‐K137R, Flag‐MDA5‐K169R, and Flag‐MDA5‐K174R were kindly provided by Zengjun Lu (Chinese Academy of Agricultural Sciences). Expression plasmids for HA‐MDA5‐CARD, HA‐MDA5‐Helicase, HA‐MDA5‐CTD, HA‐MDA5‐△CTD, and HA‐MDA5‐△CARD were generously given by Hongbing Shu (Wuhan University). Expression plasmids for HA‐ISG15 were kindly provided by Wei Zhao (Shandong University).

### 2.4 Antibodies and Reagents

The following antibodies were used for immunoblotting and immunoprecipitation: anti‐RNF213 (Santa Cruz Biotechnology, sc‐293391); anti‐RIG‐I (Proteintech, 20566‐1‐AP); anti‐HA (Proteintech, 66006‐2‐Ig); anti‐FLAG (Cell Signaling Technology, #8146); anti‐His (Cell Signaling Technology, #9991); anti‐Myc (Cell Signaling Technology, #2278); anti‐Phospho‐IRF3 (Cell Signaling Technology, #4947); anti‐Phospho‐TBK1 (Cell Signaling Technology, #5483); anti‐IRF3 (ImmunoWay, YT2398); anti‐TBK1 (Abcam, ab40676); anti‐NP (Abcam, ab104870); anti‐Ubiquitin (linkage‐specific K63) (Abcam, ab271929); anti‐actin (Proteintech, 66009‐1‐Ig); HRP‐conjugated secondary antibodies: goat anti‐rabbit IgG (H+L) HRP (Biosharp, BL003A) and goat anti‐mouse IgG (H+L) HRP (Biosharp, BL001A).

The ligands and cytokines used were poly(I:C) (InvivoGen) and ISD (MedChemExpress). Recombinant human IFN‐α and IFN‐γ were procured from Sigma‐Aldrich and PeproTech, respectively.

Plasmid transfections in A549 and HEK293T cells were performed using Lipofectamine 3000 reagent (Invitrogen). Immunoprecipitation assays were carried out using Protein A/G agarose beads (Santa Cruz Biotechnology, sc‐2003).

### 2.5 Virus Infection In Vivo

RNF213 knockout (KO) mice were generated by Cyagen Biosciences. Seven‐week‐old wild‐type (WT) and KO littermates were anesthetized and inoculated intranasally with the PR8 virus at a dose of 1 × 10^3^ or 1 × 10^4^ TCID_50_ per mouse in 50 µL of phosphate‐buffered saline (PBS). Following infection, daily monitoring of body weight and survival rates was conducted. To determine lung viral load, tissues were snap‐frozen, homogenized in MEM medium (two cycles of 20 s each), and centrifuged at 2000 rpm for 15 min at 4°C. Virus titers in the supernatants were determined by calculating the 50% egg infectious dose (EID_50_) on 9‐ to 11‐day‐old specific pathogen‐free (SPF) embryonated chicken eggs, as previously described [23, 24]. For histopathological analysis, at 3 days post‐infection (dpi), mice were euthanized. Lung tissues were dissected, fixed in 4% paraformaldehyde, embedded in paraffin, sectioned, and stained with hematoxylin and eosin (H&E) using a standard protocol.

### 2.6 siRNA‐Mediated Gene Silencing

To silence the specified target genes, siRNAs and a negative control (NC) siRNA were designed and synthesized from GenePharma. The cells were then subjected to transfection with 50 nm siRNA using 2.0 µL of Lipofectamine RNAiMAX (Invitrogen) for 48 h. Following this, the cells were prepared for subsequent analysis. The siRNA sequences were as follows: human RNF213 siRNA #1, 5’‐CAAUGUCGACUUUGAUAAACU‐3’; human RNF213 siRNA #2, 5’‐CGTCCAATTTACGTCACCGCGTCAT‐3’.

### 2.7 Quantitative RT‐PCR (q‐PCR)

Total RNA from virus‐infected cells or lung tissues was isolated from virus‐infected cells or lung tissues using an RNA extraction kit (Thermo Scientific) following the manufacturer's protocol. First‐strand cDNA synthesis was performed using 1 µg of total RNA with the TransScript RT reagent kit (TransGen). Q‐PCR was performed using the SYBR Green RT‐PCR kit (Roche). Data were normalized to GAPDH expression. All reactions were performed in triplicate, and the resulting data were analyzed using the 2^−ΔΔCt^ method. The specific q‐PCR primers are detailed in Table S2.

### 2.8 Luciferase Reporter Assays

HEK293T cells were plated in 24‐well plates and co‐transfected with 125 ng of luciferase reporter plasmids along with an equivalent quantity of various expression plasmids or an empty vector control. Ten ng of pRL‐TK plasmids were used as an internal control. After 24 h transfection, luciferase activity was measured with the Dual‐Luciferase Reporter Assay system according to the manufacturer's instructions (Promega) as described [23, 24].

### 2.9 His Pull‐Down Assay

To confirm direct protein interactions, a pull‐down assay was performed in HEK293T cells using a His Protein Pull Down Kit (ACE Biotechnology, China). Cells were co‐transfected with plasmids encoding His‐tagged RNF213 and HA‐tagged MDA5 or Flag‐tagged NP. Forty‐eight hours post‐transfection, cells were lysed with lysis buffer, and the cell lysates were then centrifuged to remove insoluble debris. A total of 500 µg of whole cell lysate containing His‐RNF213 was incubated with 30 µL of pre‐equilibrated beads for 12 h at 4°C with gentle rotation. After extensive washing five times with 1 mL of Balance/Wash buffer to remove non‐specifically bound proteins, the complexes were eluted and analyzed by western blotting.

### 2.10 Western Blotting

Cell lysates were prepared by thoroughly lysing cells in RIPA buffer supplemented with 1 mm phenylmethylsulfonyl fluoride (PMSF). The lysates were then centrifuged, and the total protein concentration was quantified using a bicinchoninic acid (BCA) protein assay kit (Beyotime, China). Equal amounts of protein samples were then denatured and subjected to electrophoretic separation on sodium dodecyl sulfate (SDS)‐polyacrylamide gel. Following electrophoresis, the separated proteins were transferred onto polyvinylidene difluoride (PVDF) membranes via electroblotting. The membranes were blocked with 5% (w/v) non‐fat dry milk prepared in TBST buffer (10 mm Tris‐HCl, pH 8.0, 150 mm NaCl, 0.05% Tween‐20) for 90 min at room temperature (RT). Subsequently, the membranes were incubated overnight at 4°C with primary antibodies. After washing, the membranes were probed with species‐specific horseradish peroxidase (HRP)‐conjugated secondary antibodies for 1 h at RT. Protein‐antibody interactions were visualized using an enhanced chemiluminescence (ECL) detection system (Thermo Fisher). Detection of cytosolic proteins was normalized to β‐actin, which served as an internal loading control.

### 2.11 RNA‐seq Experiment

A549 cells were seeded in 6‐well plates (Corning, Inc., NY) in triplicate and incubated with IFN‐α (100 IU/mL) or IFN‐γ treated (1 µg/mL) for 6 h. A549 cells treated with IFN‐α or IFN‐γ for 0 h were designated the control group. Following incubation, the cells were collected, and total RNA was extracted using TRIzol reagent (Invitrogen, Carlsbad, CA). RNA quality and concentration were assessed using the NanoPhotometerÂ spectrophotometer (IMPLEN, CA, USA) and the RNA Nano 6000 Assay Kit on the Bioanalyzer 2100 system (Agilent Technologies, CA, USA), respectively. Subsequently, sequencing libraries were prepared from 1 µg of total RNA using the TruSeq Stranded Total RNA Sample Prep Kit (Illumina, San Diego, CA) following the manufacturer's protocol. The libraries were then sequenced on an Illumina HiSeq XTen system (Illumina, San Diego, CA) by CapitalBio Corporation. The identification of differentially expressed genes (DEGs) was based on a fold change ≥1 and a *p* < 0.05. These DEGs were then analyzed for enrichment in Gene Ontology (GO) biological processes and Kyoto Encyclopedia of Genes and Genomes (KEGG) pathways in order to elucidate their functional roles and biological significance.

### 2.12 Co‐Immunoprecipitation (Co‐IP)

For the Co‐IP assay, cells were harvested and lysed using 800 µL of IP lysis buffer (Thermo Scientific), which was supplemented with protease and phosphatase inhibitors. A fraction of each whole cell lysate was retained to verify protein expression levels, while 500 µg of the lysates were utilized for the Co‐IP assays. The lysates were then incubated overnight at 4°C with the indicated antibodies under continuous rotation. Subsequently, 40 µL of Protein A/G agarose was added to the mixture and incubated for 2 h at 4°C with gentle rotation. Following this, the beads were washed four times with cold lysis buffer and subjected to analysis via SDS‐PAGE and Western blotting.

### 2.13 Immunofluorescent and Confocal Microscopy

HEK293T cells cultured on coverslips were washed twice using pre‐warmed PBS and then fixed with 4% paraformaldehyde for 15 min at RT. Following this, cells were permeabilized using an immunostaining permeabilization buffer containing Triton X‐100 (Beyotime Biotechnology) for 10 min. Subsequently, cells were blocked using QuickBlock Blocking Buffer for 20 min at RT. The fixed and permeabilized cells were then incubated overnight at 4°C with primary antibodies diluted in immunostaining primary antibody dilution buffer. Following this, the coverslips were washed thrice with PBS and further incubated with Alexa Fluor 488‐conjugated or Alexa Fluor 555‐conjugated secondary antibodies for 1 h at RT. Thereafter, the coverslips were washed thrice more with PBS. For nuclear staining, the coverslips were mounted onto microscope slides using DAPI Staining Solution (Beyotime Biotechnology) for 5 min. Then, the cells were subjected to microscopy analysis with the Zeiss LSM780 confocal laser microscope.

### 2.14 Ubiquitination Assay

For analysis of the ubiquitination of MDA5 in HEK293T cells, HEK293T cells were transfected with Myc‐MDA5, HA‐Ub (WT), or HA‐Ub mutants (K48 or K63) in combination with His‐RNF213 plasmids or empty vector. Following 24 h after transfection, cells were harvested and lysed using RIPA buffer (composed of 50 mm Tris‐HCl at pH 8.0, 150 mm NaCl, 1% NP‐40, 0.1% SDS, and 1 mm EDTA) containing protease inhibitor cocktail and 10 µm deubiquitinase inhibitor N‐ethylmaleimide (NEM, Sigma). Then, the whole‐cell extracts were immunoprecipitated with anti‐Myc and analyzed by immunoblot with the indicated antibodies as described before [23, 24].

### 2.15 Quantitative Proteomic Analysis of Ubiquitination Modifications

Six‐ to 8‐week‐old wild‐type (WT) and RNF213 KO mice (n = 2) were infected intranasally with 100 TCID_50_ of PR8 virus in 50 µL PBS after anesthesia. Lung samples were collected, weighed, ground with liquid nitrogen into cell powder, and then transferred to a 5‐mL centrifuge tube. After that, four volumes of lysis buffer (8 m urea, 50 mm Tris‐HCl, pH 8.0, supplemented with 1% protease inhibitor cocktail and 10 mm N‐ethylmaleimide (NEM) to preserve ubiquitination) were added, followed by sonication on ice (3 cycles of 10 s on, 10 s off). Protein concentration was determined using a bicinchoninic acid (BCA) assay kit (Beyotime). Protein samples were reduced and digested as previously described [25]. After trypsin digestion, 5 mg of tryptic peptides were dissolved in NETN buffer (100 mm NaCl, 1 mm EDTA, 50 mm Tris‐HCl, 0.5% NP‐40, pH 8.0), and the supernatants were transferred into pre‐cleaned ubiquitination resins (Product No. PTM‐1104, PTM Bio, Inc., China), and incubated overnight at 4°C with gentle shaking. After incubation, the resins were washed four times with NETN buffer and twice with deionised water. Then, the resin‐bound peptides were rinsed three times with 0.1% trifluoroacetic acid, and the bound peptides were collected and lyophilized under vacuum. After draining, the peptides were desalted with C18 ZipTips (Millipore), and the desalted rinses were again lyophilized under vacuum. Finally, LC/MS analysis was performed as previously described [25]. In brief, the separated peptides were analyzed on an Orbitrap Fusion mass spectrometer (ThermoFisher Scientific) with a nano‐electrospray ion source. The electrospray voltage applied was 2.0 kV. The raw MS data were processed using the MaxQuant search engine (v.1.6.15.0). The false discovery rate (FDR) for peptides and proteins was set to 1%. For ubiquitination site quantification, only sites detected in at least two replicates per group were included in statistical analysis. Differential ubiquitination was assessed using a fold‐change threshold of >1.2 for KO vs WT comparisons.

### 2.16 Statistical Analysis

In the present study, all bar charts were constructed to present data as means ± standard error of the mean (SEM). The statistical analyses were conducted using GraphPad Prism software, version 7.00 (GraphPad Software Inc., USA). The Kaplan‐Meier method was employed for survival analysis. Differences in means were considered statistically significant at *p* < 0.05. And significance levels are as follow: ^*^ *p* < 0.05; ^**^ *p* < 0.01; ^***^ *p* < 0.001; NS., non‐significant.

## 3 Results

### 3.1 Identification of ISGs in A549 Cells after IFN‐α and IFN‐γ Treatment

To characterize the IFN response, transcriptomic analysis was performed on A549 cells treated with IFN‐α (100 U/mL) or IFN‐γ (1 µg/mL) for 6 h. IFN‐α treatment upregulated 535 genes and downregulated 379 genes (Figure S1A,B), while IFN‐γ upregulated 1,142 genes and downregulated 828 genes (Figure S1C,D). GO enrichment analysis showed that IFN‐α‐treated cells were enriched in processes such as “Response to type I interferon” and “Innate immune response” (Figure S1E), whereas IFN‐γ‐treated cells exhibited enrichment in “Response to cytokine” and “Defense response” (Figure S1G). KEGG pathway analysis indicated that DEGs from both treatments were primarily associated with IAV and HSV infections (Figure S1F,H). In addition, we identified 242 ISGs commonly induced by both IFN‐α and IFN‐γ (Table S1). Notably, RNF213 was among the common set of ISGs, confirming its status as a bona fide ISG. Most of these ISGs have known roles in influenza virus infection, targeting various stages of the viral life cycle. RNF213, an E3 ubiquitin ligase not previously characterized in this context, was selected for further study as a potential novel antiviral ISG against IAV infection.

### 3.2 The Expression of RNF213 Is Induced by IFN Treatment and IAV Infection

**FIGURE 1:**
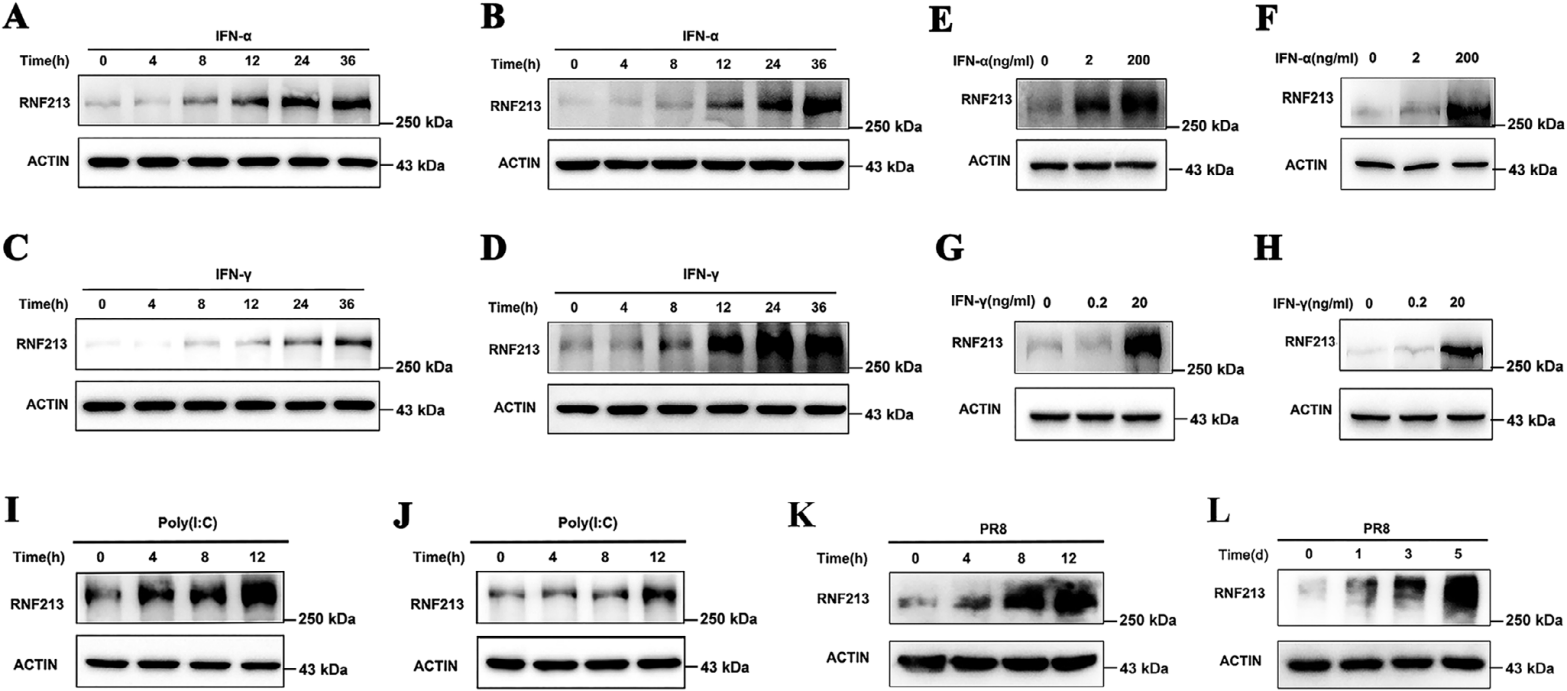
RNF213 expression is induced by IFN treatment and IAV infection. (A) After IFN‐α stimulation of A549 cells, protein samples were collected at 0 h, 4 h, 8 h, 12 h, 24 h, and 36 h, respectively, and RNF213 expression was examined by immunoblotting. (B) RNF213 expression in IFN‐α‐treated HEK293T cells at different time points was examined by western blotting. (C) RNF213 expression in IFN‐γ‐treated A549 cells at different time points was examined by western blotting. (D) RNF213 expression in IFN‐γ‐treated HEK293T cells at different time points was examined by western blotting. (E) RNF213 expression was detected by immunoblotting after stimulation of A549 cells with 0 ng/mL, 2 ng/mL, and 200 ng/mL of IFN‐α at 24 h. (F) RNF213 expression was detected by immunoblotting after stimulation of HEK293T cells with 0 ng/mL, 2 ng/mL, and 200 ng/mL of IFN‐α at 24 h. (G) RNF213 expression was detected by immunoblotting after stimulation of A549 cells with 0 ng/mL, 0.2 ng/mL, and 20 ng/mL of IFN‐γ at 24 h. (H) RNF213 expression was detected by immunoblotting after stimulation of HEK293T cells with 0 ng/mL, 0.2 ng/mL, and 20 ng/mL of IFN‐γ at 24 h. (I) RNF213 expression in HEK293T cells transfected with poly(I:C) at 0 h, 4 h, 8 h, and 12 h after transfection was examined by western blotting. (J) RNF213 expression in A549 cells transfected with poly(I:C) at 0 h, 4 h, 8 h, and 12 h after transfection was examined by western blotting. (K) RNF213 level in A549 cells infected with live PR8 virus at an MOI of 1 at 0 hpi, 4 hpi, 8 hpi, and 12 hpi was examined by western blotting. (L) RNF213 level in the lung tissue homogenates of mice challenged with PR8 (1 × 10^3^ TCID_50_) virus at the indicated time points was evaluated by western blotting. (A) to (H): β‐actin is shown as a loading control. The representative results are shown.

To determine if RNF213 is an ISG, we treated A549 and HEK293T cells with IFN‐α or IFN‐γ. Both IFNs significantly increased RNF213 protein levels in a time‐ and dose‐dependent manner (Figure 1A–H). Furthermore, transfection with the viral mimic poly(I:C) induced a progressive upregulation of RNF213 in both HEK293T and A549 cells (Figure 1I,J). To assess this during viral infection, we used the influenza A virus (PR8). PR8 infection significantly increased RNF213 expression in A549 cells at 4 h, 8 h, and 12 h post‐infection (hpi) and in mouse lung tissue at 1 dpi, 3 dpi, and 5 dpi (Figure 1K,L). The stronger and more rapid induction of RNF213 by poly(I:C) and IAV infection, compared to exogenous IFN‐β, is likely attributable to the synergistic and self‐amplifying nature of the innate immune signaling cascade triggered by viral components. Collectively, these findings indicate that RNF213 is an ISG induced by both IFN treatment and IAV infection.

### 3.3 RNF213 Inhibits IAV Replication

**FIGURE 2:**
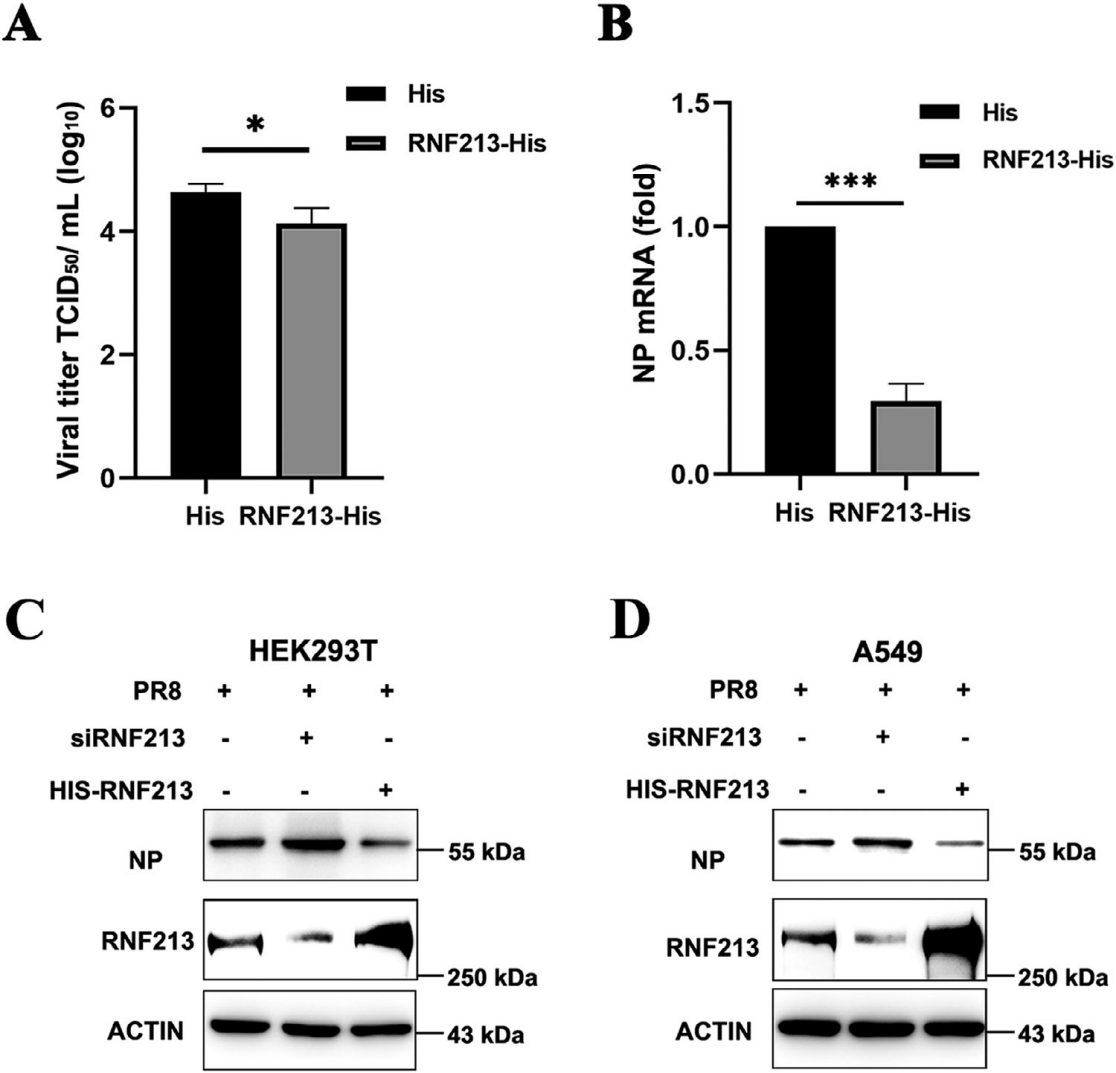
RNF213 inhibits IAV replication. (A) A549 cells were transfected with RNF213‐His plasmids or empty control for 24 h and then infected with PR8 virus at 1.0 MOI. Viral titers were measured by TCID_50_ assay at 18 hpi. Data presented as means ± SEMs and are representative of three independent experiments. Statistical significance was determined by a two‐tailed unpaired Student's *t*‐test. ^*^ *p* <0.05; ^**^ *p* <0.01. (B) A549 cells were transfected with negative control or RNF213‐overexpressing plasmids, and 24 h after transfection, the cells were infected with PR8 virus at an MOI of 1. The mRNA of NP was analyzed by q‐PCR at 18 hpi. Data presented as means ± SEMs and are representative of three independent experiments. Statistical significance was determined by a two‐tailed unpaired Student's t‐test. ^**^ *p* <0.01; ^***^ *p* <0.001. (C) HEK293T cells were transfected with RNF213‐targeting siRNA or RNF213‐overexpressing plasmids along with a negative control. After 24 h, the cells were infected with PR8 virus at 1.0 MOI, and expression of NP was measured by western blotting at 18 hpi. (D) A549 cells were transfected with RNF213‐targeting siRNA or RNF213‐overexpressing plasmids along with a negative control. After 24 h, the cells were infected with PR8 virus at 1.0 MOI, and expression of NP was measured by western blotting at 18 hpi. Data are representative of three independent experiments.

To further assess the antiviral effect of RNF213 against IAV, experiments were conducted in which His‐tagged RNF213 was overexpressed in A549 cells. These cells were subsequently infected with the PR8 virus at a multiplicity of infection (MOI) of 1. The results showed a significant reduction in viral titers in RNF213‐overexpressing cells compared to control cells at 18 hpi (Figure 2A) (*p* < 0.05). Moreover, the results demonstrate a marked suppression of viral NP mRNA levels in PR8‐infected A549 cells at 18 hpi (Figure 2B) (*p* < 0.001). Furthermore, transfection of cells with RNF213‐targeting siRNA significantly decreased the protein expression of RNF213 (Figure S2). In addition, transfection of RNF213‐targeting siRNA increased the protein level of viral NP protein in HEK293T and A549 cells (Figure 2C,D), while overexpression of RNF213 inhibited the expression of NP in HEK293T and A549 cells after PR8 virus infection (Figure 2C,D). Overall, these findings indicate that RNF213 plays a role in modulating influenza virus infection.

### 3.4 RNF213 KO Mice Are Susceptible to Influenza Virus Infection

**FIGURE 3:**
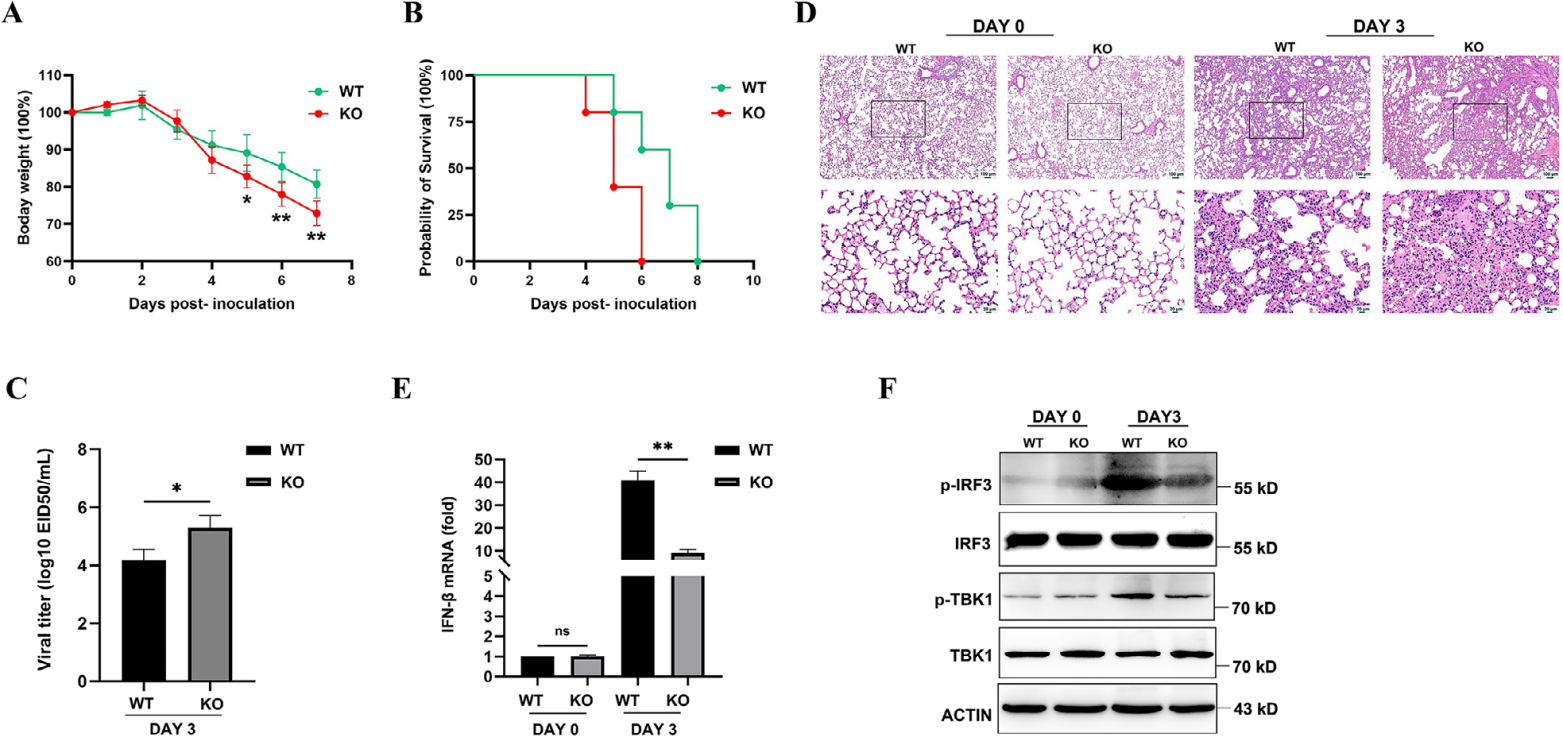
RNF213 KO mice are susceptible to IAV infection. (A) WT and RNF213 KO mice (*n* = 6) were infected with 1 × 10^3^ TCID_50_ of PR8 virus. Changes in body weight were monitored daily. Data presented as means ± SD. (B) Survival of WT and RNF213 KO mice (*n* = 10) after infection with 1 × 10^4^ TCID_50_ of PR8 virus. Kaplan‐Meier Survival Curves were compared using the log‐rank (Mantel‐Cox) analysis. ^*^ *p* < 0.05; ^**^ *p* < 0.01. (C) Viral titers in the lungs of WT and RNF213 KO mice at 3 dpi after PR8 infection were determined by EID_50_ assay. Error bars indicate SEM. ^*^ *p* < 0.05; ^**^ *p* < 0.01. (D) H&E staining of lung tissues from WT and RNF213 KO mice after challenge with 1 × 10^3^ TCID_50_ of PR8 virus at 3 dpi. Representative of H&E staining images from 6 mice per group of three independent experiments. (E) The mRNA expression of IFN‐β in lung tissues of WT and KO mice infected with 1 × 10^3^ TCID_50_ of PR8 virus at 3 dpi was determined by RT‐PCR. Data presented as means ± SEMs and are representative of three independent experiments. ^*^ *p* < 0.05; ^**^ *p* < 0.01. (F) WT and RNF213 KO mice (*n* = 3) were infected with 1 × 10^3^ TCID_50_ of PR8 virus. Activation of p‐IRF3 and p‐TBK1 in the lung tissues at the indicated time points was measured by western blotting. Data are representative of three independent experiments.

To explore the role of RNF213 in IAV infection in vivo, the body weight of both WT and RNF213 KO mice was monitored after infection with PR8 virus at a dose of 1 × 10^3^ TCID_50_. Prior to the experiment, the genotypes of WT and KO mice were confirmed by PCR (Figure S3). The results showed that PR8 virus‐infected KO mice exhibited significantly higher body weight loss than WT mice at 5 dpi, 6 dpi, and 7 dpi (Figure 3A). To further investigate the functional significance of RNF213 in influenza virus infection, we intranasally infected RNF213 KO mice with PR8 virus at a higher dose of 1 × 10^4^ TCID_50_ and monitored the survival of the mice. The results revealed a significantly lower survival rate in PR8‐infected RNF213 KO mice compared to their WT counterparts (*p* = 0.0038) (Figure 3B), suggesting that RNF213 plays a critical role in mitigating IAV infection in vivo. Further analysis using a 50% egg infective dose (EID_50_) assay demonstrated that viral loads in the lung tissues of RNF213 KO mice were markedly higher than those in WT mice at 3 dpi (Figure 3C) (*p* < 0.05). This indicates that the absence of RNF213 leads to enhanced viral replication. Furthermore, histological examination revealed more severe lung injury in RNF213 KO mice compared to WT mice at 3 dpi (Figure 3D). Consequently, the mRNA expression of IFN‐β in the lung tissues of PR8‐infected mice at 3 dpi was significantly suppressed in response to RNF213 deficiency (Figure 3E) (*p* < 0.01). In addition, PR8 virus‐induced p‐IRF3 and p‐TBK1 activation were inhibited in the lung tissues of RNF213 KO mice as compared with WT mice at 3 dpi (Figure 3F). Therefore, RNF213 is required for host defense against influenza virus infection in vivo.

### 3.5 RNF213 Positively Regulates the RLR‐Mediated IFN‐β Signaling Pathway

**FIGURE 4:**
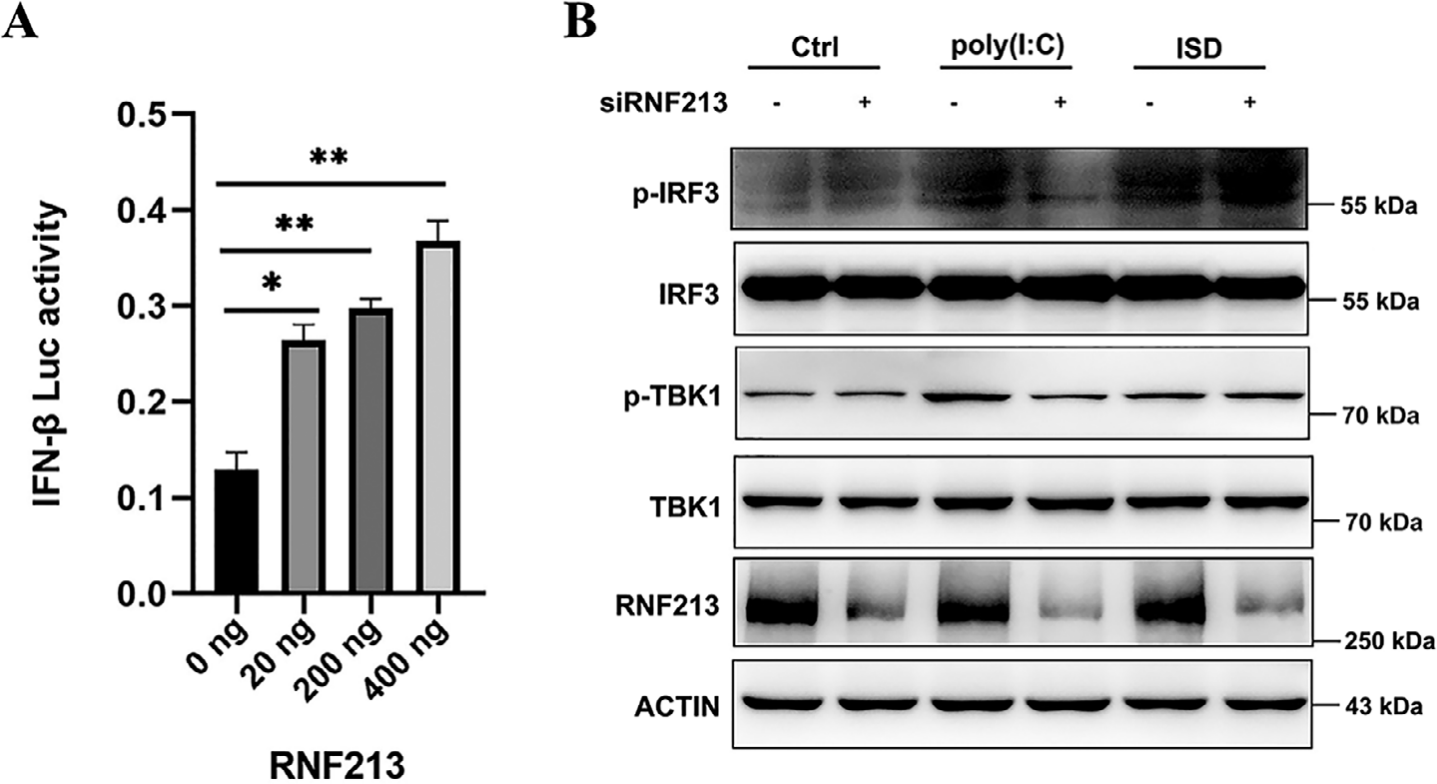
RNF213 positively regulates the RLR‐mediated IFN‐β signaling pathway. (A) HEK293T cells were transfected with IFN‐β reporter plasmid together with RNF213 expression plasmids at doses of 0, 20, 200, and 400 ng. IFN‐β activity was measured using luciferase reporter assays after treatment with poly(I:C) for 12 h, respectively. Statistical significance was determined by a two‐tailed unpaired Student's *t*‐test. ^*^ *p* < 0.05; ^**^ *p* < 0.01. (B) HEK293T cells were transfected with RNF213‐targeting siRNA or a negative control. After 24 h, the cells were transfected with poly(I:C) or ISD for 12 h, and expression of p‐IRF3, IRF3, p‐TBK1, and TBK1 was measured by western blotting. Data are representative of three independent experiments.

To explore the role of RNF213 in antiviral immune responses, the impact of RNF213 on the induction of IFN‐β expression downstream of various PRRs was examined using an IFN‐β promoter reporter assay. Cytosolic poly(I:C), a known activator of IFN‐β expression via RIG‐I and MDA‐5 pathways in HEK293T cells, was utilized as a model stimulus [26]. The findings revealed that the expression of RNF213 significantly enhanced poly(I:C)‐induced IFN‐β production at a dose of 20 ng (Figure 4A) (*p* < 0.05). Furthermore, transfection of RNF213 into HEK293T cells resulted in a substantial augmentation in poly(I:C)‐induced IFN‐β expression at concentrations of 200 ng and 400 ng, thereby demonstrating a discernible dose‐dependent effect (Figure 4A) (*p* < 0.01). In addition, transfection of RNF213‐targeting siRNA into HEK293T cells markedly suppressed poly(I:C)‐induced activation of p‐IRF3 and p‐TBK1 (Figure 4B). However, HEK293 cells transfected with RNF213‐targeting siRNA followed by ISD (a synthetic double‐stranded DNA (dsDNA) sequence recognized by cGAS) transfection did not change the expression of p‐IRF3 and p‐TBK1 as compared with the control group (Figure 4B). These results underscore the role of RNF213 as a positive regulator of IFN‐β production and highlight its contribution to antiviral immune responses.

### 3.6 RNF213 Targets MDA5

**FIGURE 5:**
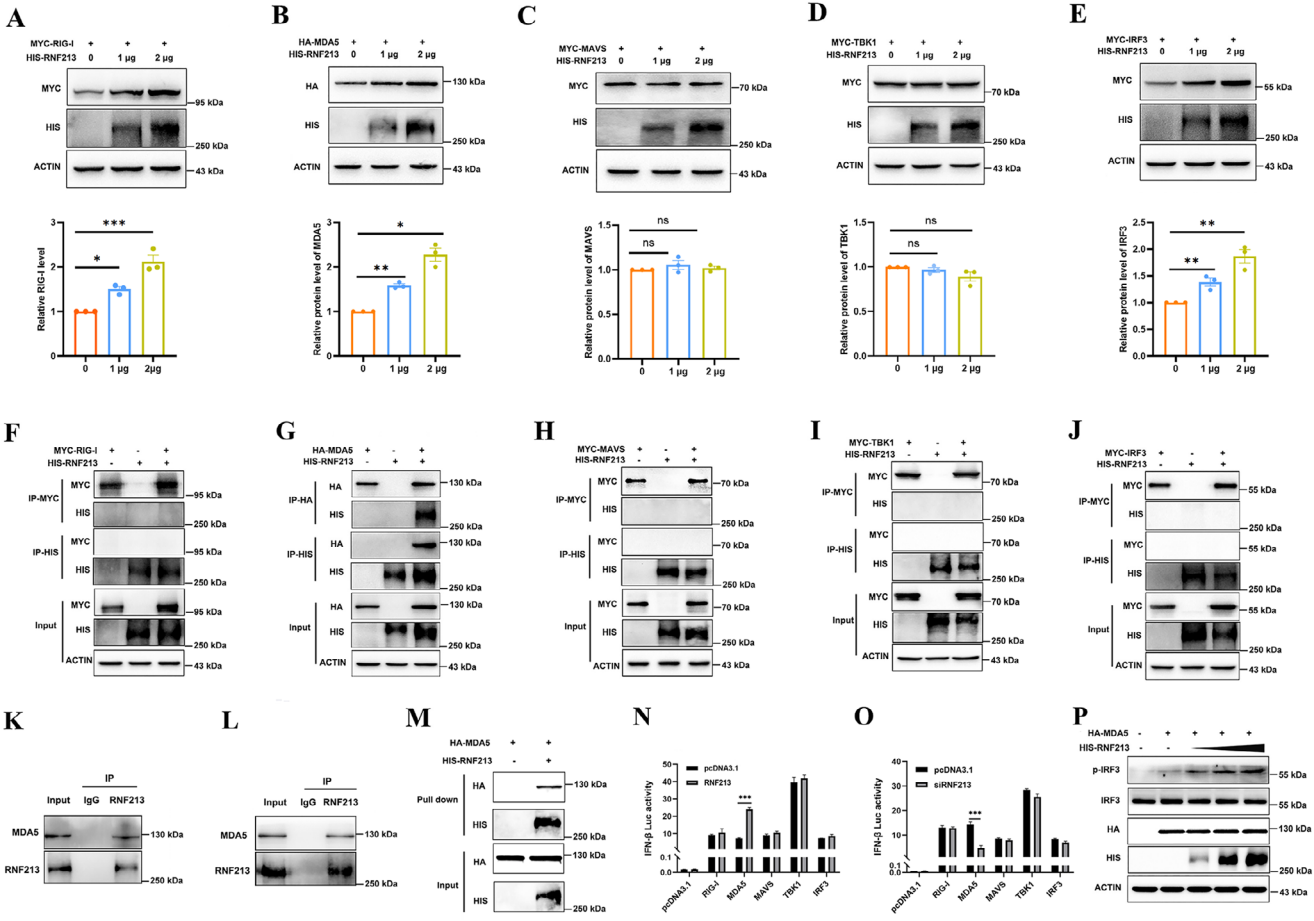
RNF213 targets MDA5. (A) HEK293T cells were co‐transfected with RIG‐I‐Myc expression plasmids with RNF213‐His expression vectors at doses of 0 µg, 1 µg, and 2 µg. After 24 h of transfection, protein expression of RIG‐I‐Myc was measured by western blotting. (B) MDA5‐HA expression in HEK293T co‐transfected with MDA5‐HA plasmids and different concentrations of RNF213‐His expression vectors for 24 h was examined by western blotting. (C) MAVS‐Myc expression in HEK293T co‐transfected with MAVS‐Myc plasmids and different concentrations of RNF213‐His expression vectors for 24 h was detected by western blotting. (D) TBK1‐Myc expression in HEK293T co‐transfected with TBK1‐Myc plasmids and different concentrations of RNF213‐His expression vectors for 24 h was analyzed by western blotting. (E) IRF3‐Myc expression in HEK293T co‐transfected with IRF3‐Myc plasmids and different concentrations of RNF213‐His expression vectors for 24 h was examined by western blotting. (F) HEK293T cells were co‐transfected with plasmids encoding RIG‐I‐Myc together with a plasmid encoding RNF213‐His. Cell lysates were collected at 24 h after transfection and immunoprecipitated with anti‐Myc or anti‐His antibody and probed with anti‐His or anti‐Myc antibody. (G) Co‐IP analysis of the interaction between HA‐tagged MDA5 and His‐tagged RNF213 in HEK293T cells at 24 h after transfection. (H) Co‐IP analysis of the interaction between Myc‐tagged MAVS and His‐tagged RNF213 in HEK293T cells at 24 h after transfection. (I) Co‐IP analysis of the interaction between Myc‐tagged TBK1 and His‐tagged RNF213 in HEK293T cells at 24 h after transfection. (J) Co‐IP analysis of the interaction between Myc‐tagged IRF3 and His‐tagged RNF213 in HEK293T cells at 24 h after transfection. (K) A549 cells were transfected with poly(I:C) for 12 h. Cell lysates were immunoprecipitated with IgG or anti‐RNF213 antibody and probed with anti‐MDA5 antibody. (L) A549 cells were infected with PR8 virus at an MOI of 1 for 12 h. Cell lysates were immunoprecipitated with IgG or anti‐RNF213 antibody and probed with anti‐MDA5 antibody. (M) Direct binding of RNF213 and MDA5 using the His pull‐down assay. The pull‐down bands were detected by Western blotting. (N) HEK293T cells were co‐transfected with plasmids encoding RIG‐I, MDA5, MAVS, TBK1, or IRF3 along with empty vector or RNF213 expression plasmids. After 24 h of transfection, IFN‐β activity was measured by luciferase reporter assays. Luciferase levels were normalized to Renilla values. Values shown as fold change over empty vector control. (O) Luciferase activity of IFN‐β‐luciferase reporter in HEK293T cell co‐transfected with plasmids encoding RIG‐I, MDA5, MAVS, TBK1, or IRF3, along with a negative control or siRNA of the RNF213 gene. (P) IRF3 and p‐IRF3 expression levels in HEK293T cells co‐transfected with MDA5 plasmids tagged with HA and increasing concentration of RNF213 expression plasmids tagged with His were detected by immunoblotting at 24 h after transfection. (A) to (L), and (P): β‐actin is shown as a loading control. The representative results are shown. (N) and (O): Data presented as means ± SEMs and are representative of three independent experiments. Statistical significance was determined by a two‐tailed unpaired Student's *t*‐test. ^*^ *p* < 0.05; ^**^ *p* < 0.01.

To elucidate the molecular mechanisms underlying RNF213‐mediated regulation of the type I IFN signaling pathway, HEK293T cells were co‐transfected with RNF213 overexpressing plasmids at concentrations of 1 µg and 2 µg, along with plasmids encoding RIG‐I, MDA5, MAVS, TBK1, or IRF3. The findings demonstrated that RNF213 expression was dose‐dependent, resulting in elevated protein levels of RIG‐I, MDA5, and IRF3 (Figure 5A,B,E), while the expression of MAVS and TBK1 remained unaltered (Figure 5C,D). To further identify the specific molecular targets of RNF213, the interactions between RNF213 and RIG‐I, MDA5, MAVS, TBK1, or IRF3 were assessed in HEK293T cells. The results revealed that RNF213 selectively interacted with MDA5 (Figure 5G) but showed no interaction with RIG‐I, MAVS, TBK1, or IRF3 (Figure 5F,H,I,J). Endogenous RNF213 readily interacted with MDA5 after poly(I:C) transfection (Figure 5K) and PR8 infection (Figure 5L) for 12 h in A549 cells. Moreover, the direct interaction between RNF213 and MDA5 was confirmed (Figure 5M). Further investigation into the functional impact of RNF213 overexpression was conducted using reporter assays to measure IFN‐β promoter activity. Notably, RNF213 significantly enhanced MDA5‐mediated activation of the IFN‐β‐luciferase reporter compared to the negative control (*p* < 0.001), whereas no such effect was observed with RIG‐I, MAVS, TBK1, or IRF3 (Figure 5N). In contrast, silencing of RNF213 expression significantly impeded the activation of the IFN‐β‐luciferase reporter induced by MDA5 (Figure 5O) (*p* < 0.001). To further explore the regulatory role of RNF213 in MDA5‐mediated IRF3 activation, HEK293T cells were co‐transfected with HA‐tagged MDA5 plasmids and increasing concentrations of His‐tagged RNF213 plasmids. Immunoblotting analysis revealed that RNF213 overexpression enhanced MDA5‐induced p‐IRF3 in a dose‐dependent manner (Figure 5P). Taken together, these findings indicate that RNF213 potentiates MDA5 activation, thereby positively regulating IFN‐β production.

### 3.7 RNF213 Binds to MDA5 via Its AAA+ ATPase and E3 Ligase Domains

**FIGURE 6:**
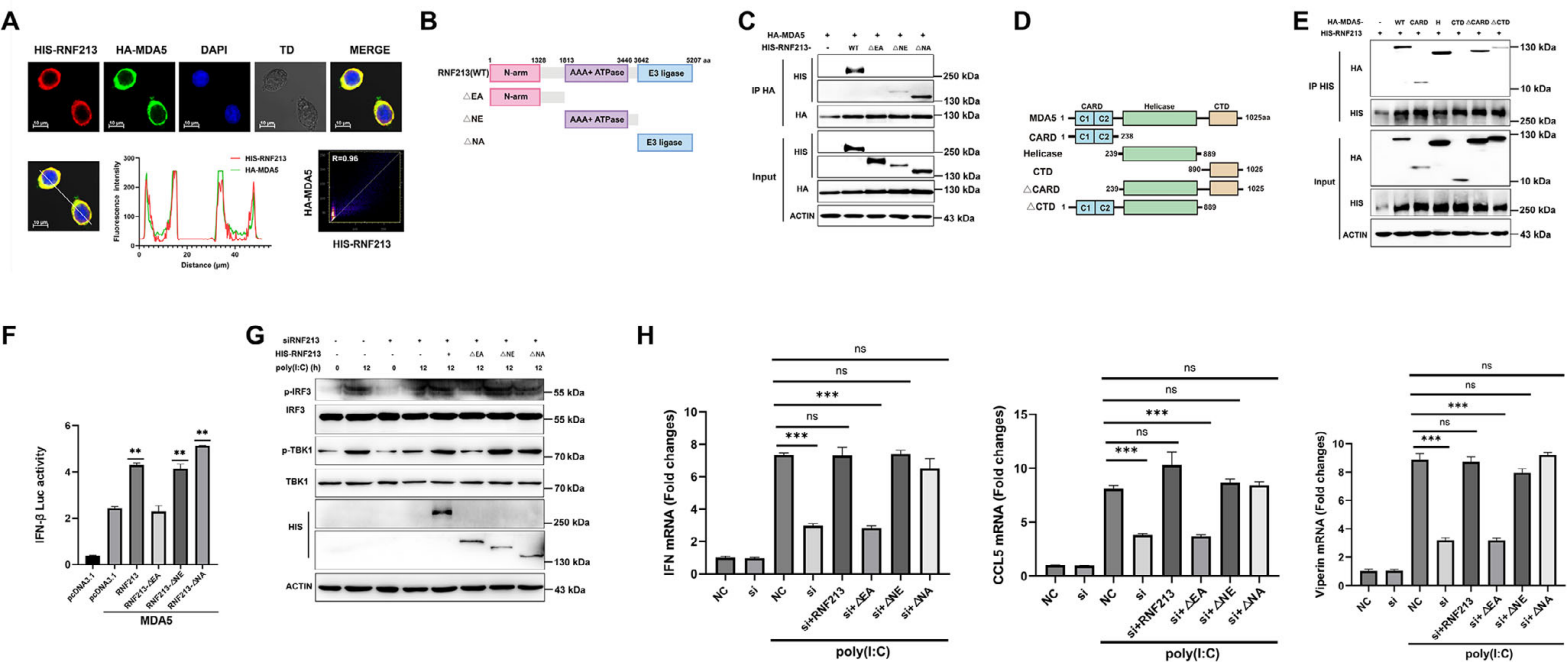
RNF213 binds to MDA5. (A) Confocal microscopy of HEK293T cells co‐transfected with vectors encoding His‐tagged RNF213 and HA‐tagged MDA5 for 24 h. Nuclei were detected with DAPI (blue). Scale bars: 10 µm. The Pearsons and Manders’ coefficients of indicated proteins along the plotted lines, as analyzed by ImageJ line scan analysis. (B) Schematic diagram of RNF213 and its truncation mutants. (C) His‐tagged RNF213 mutants and HA‐MDA5 were individually transfected into HEK293T cells. The cell lysates were immunoprecipitated with anti‐HA antibody and then immunoblotted with the indicated antibodies. (D) Schematic diagram of MDA5 and its truncation mutants. (E) HA‐tagged MDA5 mutants and His‐tagged RNF213 were individually transfected into HEK293T cells. The cell lysates were immunoprecipitated with anti‐His antibody and then immunoblotted with the indicated antibodies. (F) HEK293T cells were co‐transfected with HA‐tagged MDA5 plasmids together with empty vector or His‐tagged RNF213 full‐length or its truncated mutants. After 24 h of transfection, IFN‐β activity was measured by luciferase reporter assays. Data presented as means ± SEMs and are representative of three independent experiments. Statistical significance was determined by a two‐tailed unpaired Student's *t*‐test. ^*^ *p* < 0.05; ^**^ *p* < 0.01. (G) HEK293T cells were co‐transfected with His‐tagged RNF213 full‐length or its truncated mutants, along with a negative control or siRNA of the RNF213 gene for 24 h. Following transfection with poly(I:C), samples were collected at 0 h and 12 h, and protein levels were detected by immunoblotting with the indicated antibodies. (H) HEK293T cells were co‐transfected with His‐tagged RNF213 full‐length or its truncated mutants, along with a negative control or RNF213‐targeting siRNA for 24 h. Following transfection with poly(I:C) for 12 h, mRNA expression of IFN‐β, CCL5, and viperin was determined by q‐PCR. (A), (C), (E), and (G): Similar results were obtained from three independent experiments.

To investigate the mechanisms underlying MDA5 activation by RNF213, an analysis was conducted of the interaction between RNF213 and MDA5 in HEK293T cells co‐transfected with plasmids expressing His‐tagged RNF213 and HA‐tagged MDA5. Immunofluorescence analysis demonstrated that full‐length RNF213 colocalized with MDA5 in the cytoplasm (Figure 6A). RNF213 is unique in that it is the sole known protein that exerts both AAA+ ATPase and ubiquitin ligase activities [27]. To identify the specific domain of RNF213 responsible for its interaction with MDA5, a series of truncated RNF213 mutants was generated (Figure 6B). Immunoprecipitation assays revealed that MDA5 interacts with both the AAA+ ATPase and E3 ligase domains of RNF213, but not with the Narm domain (Figure 6C). These findings suggest that RNF213 binds to MDA5 specifically through its AAA+ ATPase and E3 ligase domains.

To further characterize this interaction, a series of MDA5 deletion mutants was constructed (Figure 6D). Co‐IP results demonstrated that removing the CARD and helicase domains of MDA5 disrupted its interaction with RNF213, indicating that these regions are critical for binding (Figure 6E). Furthermore, to evaluate the functional significance of the AAA+ ATPase and E3 ligase domains of RNF213 in MDA5‐mediated IFN production, we conducted a measurement of IFN‐β promoter activity in HEK293T cells co‐transfected with HA‐tagged MDA5 plasmids and either full‐length His‐tagged RNF213 or its truncated mutants. Following 24 h after transfection, IFN‐β activity was found to be significantly elevated in cells expressing full‐length RNF213, RNF213‐△NE, and RNF213‐△NA in comparison to the control group (Figure 6F) (*p* < 0.01). Conversely, no significant change in IFN‐β activity was observed in cells expressing RNF213‐△EA, suggesting that both the AAA+ ATPase and E3 ligase domains are essential for RNF213‐mediated induction of MDA5‐driven IFN expression (Figure 6F). Furthermore, poly(I:C)‐induced activation of p‐TBK1 and p‐IRF3 was markedly enhanced in cells expressing full‐length RNF213, as well as the RNF213‐△NE, and RNF213‐△NA truncation mutants, relative to the control group. In contrast, this potentiating effect was weakened in cells expressing the RNF213‐△EA mutant (Figure 6G). In addition, overexpression of full‐length RNF213, RNF213‐△NE, and RNF213‐△NA rescued the inhibitory transcription of IFN‐β, CCL5, and viperin in RNF213‐targeting siRNA‐transfected HEK293T cells after poly(I:C) transfection, whereas this ascending effect was inhibited in RNF213‐△EA‐overexpressing HEK293T cells (Figure 6H). In summary, these results demonstrate that RNF213 interacts with MDA5 through its AAA+ ATPase and E3 ligase domains, highlighting their critical role in regulating MDA5‐dependent immune signaling.

### 3.8 RNF213 Promotes MDA5 Ubiquitination

**FIGURE 7:**
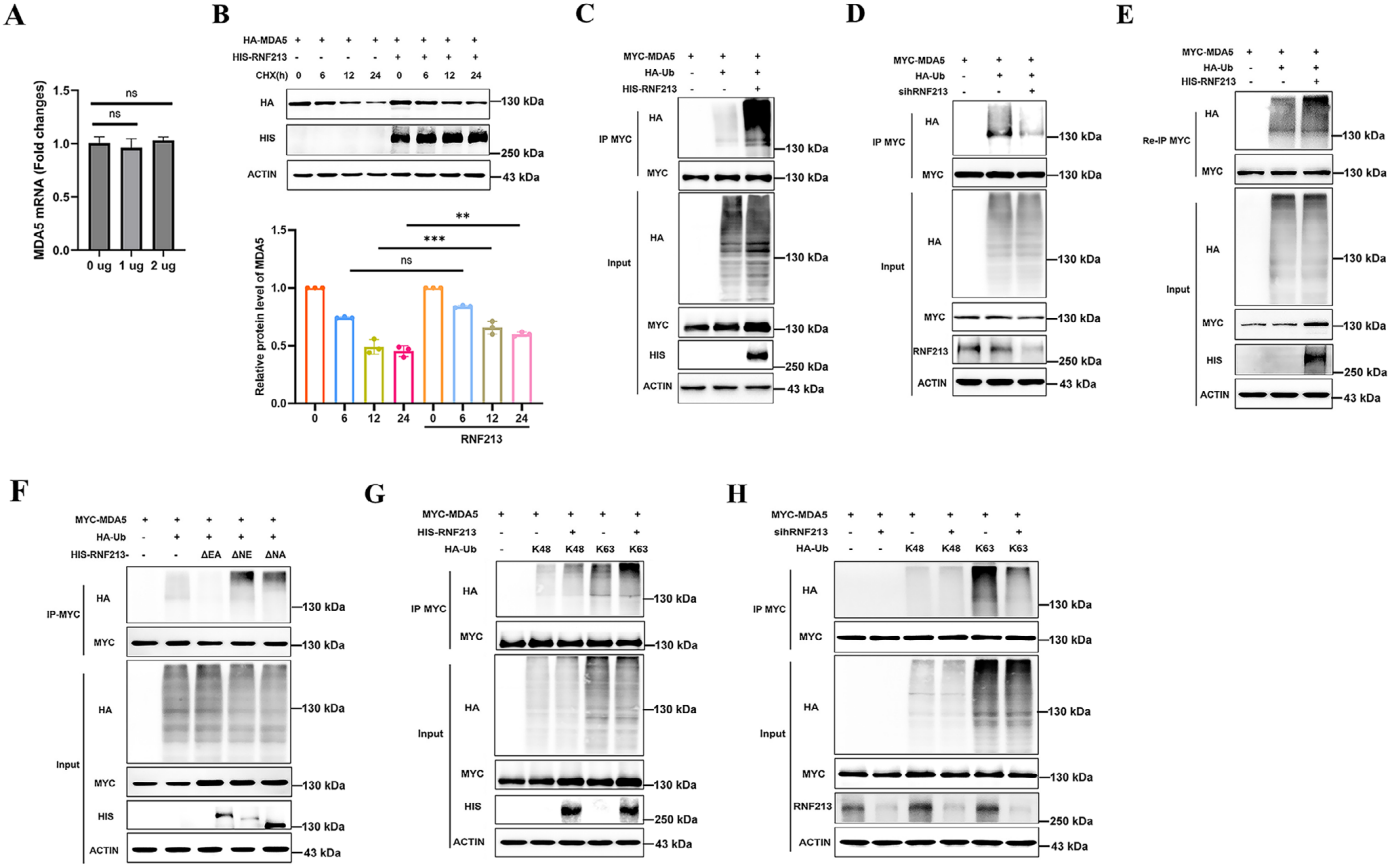
RNF213 promotes MDA5 ubiquitination. (A) MDA5 mRNA expression in A549 cells transfected with His‐tagged RNF213 plasmids at a dose of 0 µg, 1 µg, and 2 µg for 24 h was quantified by q‐PCR. (B) HEK293T cells were co‐transfected with HA‐tagged MDA5 plasmids together with empty vector or His‐tagged RNF213 plasmids. After 24 h of transfection, the protein expression level of MDA5 was detected by immunoblotting after adding CHX treatment for 0 h, 6 h, 12 h, and 24 h, respectively. (C) Co‐IP analysis of MDA5 ubiquitination in HEK293T cells transfected with expression plasmids encoding Myc‐tagged MDA5 and HA‐tagged ubiquitin together with empty vector or His‐RNF213 plasmids for 24 h. (D) Co‐IP analysis of MDA5 ubiquitination in HEK293T cells transfected with expression plasmids encoding Myc‐tagged MDA5 and HA‐tagged ubiquitin after negative control or RNF213‐targeting siRNA transfection for 24 h. (E) Lysates from HEK293T cells transfected with Myc‐tagged MDA5 and HA‐tagged ubiquitin, together with empty vector or His‐RNF213 expression plasmids for 24 h were subjected to immunoprecipitation with anti‐Myc antibody. The immunoprecipitated proteins were further subjected to immunoprecipitation with anti‐Myc antibody followed by western blotting analysis with the indicated antibodies. (F) HEK293T cells were co‐transfected with Myc‐tagged MDA5 plasmids and HA‐tagged ubiquitin together with empty vector or RNF213 truncated mutants. Cell lysates were collected at 24 h after transfection and immunoprecipitated with anti‐Myc antibody and probed with the indicated antibodies. (G) Co‐IP analysis of MDA5 ubiquitination in HEK293T cells transfected with expression plasmids encoding Myc‐tagged MDA5, HA‐ubiquitin‐K48, HA‐ubiquitin‐K63, together with empty vector or His‐tagged RNF213 for 24 h. (H) Co‐IP analysis of MDA5 ubiquitination in HEK293T cells transfected with expression plasmids encoding Myc‐tagged MDA5, HA‐ubiquitin‐K48, and HA‐ubiquitin‐K63 after negative control or siRNA of RNF213 transfection for 24 h. β‐actin is shown as a loading control. Similar results are obtained from three independent experiments.

To investigate the potential influence of RNF213 on the stability of MDA5 mRNA expression and protein synthesis, q‐PCR and a cycloheximide (CHX) chase assay were conducted. Overexpression of RNF213 did not change MDA5 mRNA expression in A549 cells (Figure 7A). The CHX chase experiments demonstrated that overexpressing RNF213 enhanced the stability of MDA5, significantly reducing its degradation rate at 12 h and 24 h post‐CHX treatment (Figure 7B). The RNF family represents the largest group of E3 ubiquitin ligases, many of which are known to exhibit E3 ubiquitin ligase activity. Several RNF proteins have been identified as regulators of innate immune signaling pathways during viral infections. To further explore whether RNF213 modulates MDA5 ubiquitination, HEK293T cells were used to express Myc‐tagged MDA5 and HA‐tagged ubiquitin, along with either a negative control, His‐RNF213 plasmids, or RNF213‐targeting siRNA. The findings indicated a significant increase in MDA5 ubiquitination levels following RNF213 expression (Figure 7C). Conversely, silencing RNF213 in HEK293T cells led to a reduction in MDA5 ubiquitination (Figure 7D). To ensure that the experimental outcomes were not influenced by the presence of other protein molecules, a secondary immunoprecipitation was performed. The results of the secondary immunoprecipitation confirmed that the level of ubiquitination of MDA5 continued to be increased by RNF213 expression (Figure 7E), suggesting a role for RNF213 in the ubiquitination of MDA5. Crucially, the ubiquitination of MDA5 by RNF213 was found to depend on the AAA+ ATPase domain and the E3 ligase domain, as the ubiquitination effect was markedly diminished in the presence of RNF213‐△EA (Figure 7F). This underscores the functional importance of these domains in RNF213‐mediated ubiquitination processes.

In order to investigate the mechanism by which RNF213 mediates the polyubiquitination of MDA5, HEK293T cells were co‐transfected with expression plasmids encoding Myc‐tagged MDA5, mutant forms of ubiquitin in which all lysine residues except the one at position 63 (HA‐Ub‐K63) or 48 (HA‐Ub‐K48) were mutated to arginine, together with empty vector or His‐tagged RNF213. The results demonstrated that RNF213 significantly enhanced the K63‐linked ubiquitination of MDA5, while it had no observable impact on the K48‐linked ubiquitination of MDA5 (Figure 7G). Furthermore, the level of K63‐linked ubiquitination of MDA5 was reduced after RNF213 silencing, while the level of K48‐linked ubiquitination of MDA5 was not obviously changed (Figure 7H). In addition, RNF213 also enhanced ISG15 conjugation to MDA5, independently of K137/K743 (Figure S4). Collectively, these findings suggest that RNF213 specifically facilitates the K63‐linked polyubiquitination of MDA5.

### 3.9 RNF213 Promotes MDA5 Ubiquitination at Lys137 and Lys743

**FIGURE 8:**
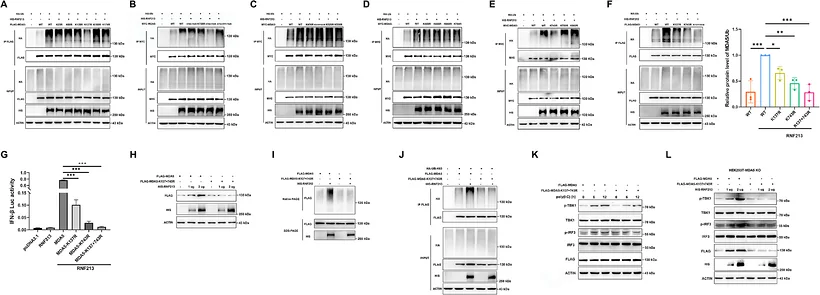
RNF213 promotes MDA5 ubiquitination at Lys137 and Lys743. (A–E) Immunoblotting of HEK293T cells co‐transfected with Flag‐tagged MDA5, HA‐ubiquitin‐K48 or MDA5 mutants bearing substitutions of K23R, K43R, K68R, K128R, K137R, K169R, K174R, K700R, K703R, K726R, K743R, K750R, K772R, K777R or K782R along with empty vector or His‐tagged RNF213 for 24 h. (F) Co‐IP analysis of MDA5 ubiquitination in HEK293T cells transfected with Flag‐tagged MDA5, HA‐ubiquitin‐K48, or MDA5 mutants bearing substitutions of K137R, K743R, or K137 plus 743R, along with empty vector or His‐tagged RNF213 for 24 h. (G) HEK293T cells were co‐transfected with empty vector or His‐tagged RNF213 plasmids together with HA‐tagged MDA5 full‐length or its mutants bearing substitutions of K137R, K743R, or K137 plus 743R. After 24 h of transfection, IFN‐β activity was measured by luciferase reporter assays. Data presented as means ± SEMs and are representative of three independent experiments. Statistical significance was determined by a two‐tailed unpaired Student's *t*‐test. ^***^ *p* < 0.01. (H) Immunoblotting of HEK293T cells co‐transfected with different concentrations of His‐tagged RNF213, MDA5 mutants at K137R and K743R, along with empty vector or Flag‐tagged MDA5 for 24 h. (I) HEK293T cells were co‐transfected with His‐tagged RNF213, MDA5 mutants at K137R and K743R along with empty vector or Flag‐tagged MDA5 for 24 h. Whole‐cell extracts were prepared and analyzed by immunoblotting and SDD‐AGE. (J) Co‐IP analysis of MDA5 ubiquitination in HEK293T cells transfected with Flag‐tagged MDA5, HA‐ubiquitin‐K63, or MDA5 mutants at K137R and K743R, along with empty vector or His‐tagged RNF213 for 24 h. (K) MDA5^−/−^ HEK293T cells were co‐transfected with MDA5 mutants at K137R and K743R along with empty vector or Flag‐tagged MDA5 for 24 h. Following transfection with poly(I:C), samples were collected at 0 h, 6 h, and 12 h, and protein levels were detected by immunoblotting with the indicated antibodies. (L) MDA5^−/−^ HEK293T cells were co‐transfected with different concentrations of His‐tagged RNF213, MDA5 mutants at K137R and K743R, along with empty vector or Flag‐tagged MDA5 for 24 h. Whole‐cell extracts were prepared and analyzed by immunoblotting with the indicated antibodies. (A) to (F), and (H) to (L): β‐actin is shown as a loading control. Similar results are obtained from three independent experiments.

To identify the specific lysine residues targeted by RNF213 for ubiquitination, we first employed a bioinformatic prediction using Phosphosite [28], PhosphoSitePlus [29], and PTMGPT2 [30] models, which yielded 20 high‐confidence candidate sites. Based on published literature (Table S3) implicating regions surrounding K703, K743, and K777 in MDA5 regulation, we included four additional proximal sites (K700, K750, K772, and K782) in our analysis. Subsequently, each of these 24 lysine (K) residues in MDA5 was systematically mutated to arginine (R) to assess their functional role. The findings revealed that the substitution of Lys137 or Lys743 diminished the enhancement effect of RNF213 on MDA5 polyubiquitination (Figure 8A–E). Furthermore, RNF213‐enhanced ubiquitination of MDA5 was completely abolished when transfected with K137/743R‐Ub double mutants (Figure 8F). To further investigate the functional significance of Lys137 and Lys743 sites, it was demonstrated that the expression of RNF213 effectively increased the activation of IFN‐β reporter genes in the presence of wild‐type MDA5. Conversely, this stimulatory effect was significantly decreased in the presence of MDA5‐K137R (*p* < 0.001), MDA5‐K743R (*p* < 0.001), and MDA5‐K137/743R mutants (*p* < 0.001) (Figure 8G). The introduction of double knock‐in mutations at MDA5 residues K137 and K743 significantly attenuated the ability of RNF213 to enhance MDA5 protein levels (Figure 8H), facilitate MDA5 oligomer assembly (Figure 8I), and promote its polyubiquitination (Figure 8J). In MDA5^−/−^ HEK293T cells, these mutations also suppressed activation of p‐TBK1 and p‐IRF3 compared to wild‐type MDA5 (Figure 8K). Furthermore, while RNF213 expression potentiated activation of p‐TBK1 and p‐IRF3 mediated by wild‐type MDA5, this enhancing effect was inhibited by the K137/K743 double mutations (Figure 8L). These results suggest that amino acids K137 and K743 of MDA5 are essential for its ubiquitination.

### 3.10 RNF213 Ubiquitinates and Degrades Viral NP

**FIGURE 9:**
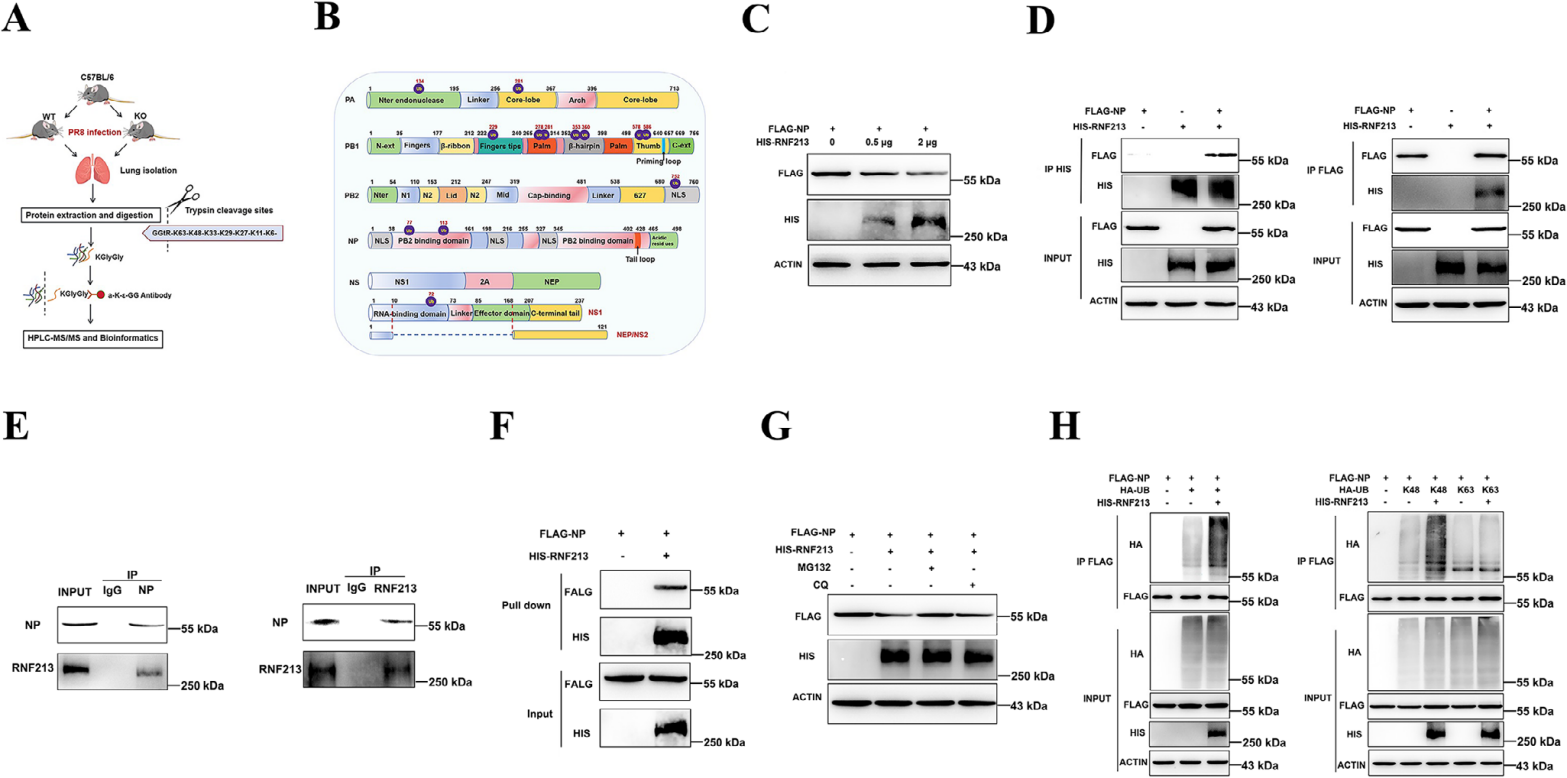
RNF213 ubiquitinates and degrades viral NP. (A) Experimental strategy. Workflow schematic for identification of K^ub^ sites in lung tissues from PR8 virus‐infected WT and RNF213 KO mice. (B) Ubiquitination sites in the functional domains of IAV proteins. The numbers in red represent the ubiquitinated lysine sites. (C) HEK293T cells were co‐transfected with NP‐Flag expression plasmids with RNF213‐His expression vectors at doses of 0 µg, 0.5 µg, and 2 µg. After 24 h of transfection, protein expression of NP‐Flag was measured by western blotting. (D) Co‐IP analysis of the interaction between Flag‐tagged NP and His‐tagged RNF213 in HEK293T cells at 24 h after transfection. (E) A549 cells were infected with PR8 virus at an MOI of 1 for 12 h. Cell lysates were immunoprecipitated with NP or anti‐RNF213 antibody and probed with the indicated antibodies. (F) Direct binding of RNF213 and NP using the His pull‐down assay. The pull‐down bands were detected by Western blotting. (G) After being transfected with Flag‐NP and either His‐RNF213 or control vector plasmids for 24 h, HEK293T cells were treated with MG132 (10 µm), CQ (50 µm), or DMSO for 6 h. After extraction, the cells were subjected to western blotting. (H) Co‐IP analysis of NP ubiquitination in HEK293T cells transfected with expression plasmids encoding Flag‐tagged NP, HA‐ubiquitin‐K48, HA‐ubiquitin‐K63, together with empty vector or His‐tagged RNF213 for 24 h. (C) to (H): β‐actin is shown as a loading control. Similar results are obtained from three independent experiments.

It has been reported that RNF213 inhibits the proliferation of cytosolic *Salmonella* through ubiquitylation of LPS [20]. To identify and quantify the ubiquitination changes that occur in viral proteins during IAV infection, a quantitative ubiquitomic approach was performed using lung tissues from PR8‐infected WT and RNF213 KO mice (Figure 9A). We identified 13 high‐confidence ubiquitinated residues on 5 viral proteins in lung tissues from PR8‐infected WT and KO mice (Table S4). Among them, one ubiquitination site was identified for PB2 (K752) and NS (K72) proteins, two ubiquitination sites were identified for PA (K134, K281) and NP (K113, K77) proteins, and seven ubiquitination sites were identified for PB1 (K281, K229, K360, K578, K278, K353, K586) protein (Figure 9B). Furthermore, our ubiquitomic analysis revealed ubiquitination on the viral NP protein at residues K77 and K113 (Table S4). Given the essential role of NP in viral replication and the trend of increased ubiquitination at K77 in KO samples, we hypothesized that RNF213 might directly target NP. To test this hypothesis, we performed the following experiments. We first co‐transfected RNF213‐overexpressing plasmids at concentrations of 0 µg, 0.5 µg, and 2 µg, along with plasmids encoding NP into HEK293T cells. The results demonstrated that RNF213 expression was dose‐dependent, resulting in decreased protein expression of NP in RNF213‐overexpressing HEK293T cells (Figure 9C). To further identify the underlying mechanism involved in the inhibition of IAV replication by RNF213, we examined the interaction between RNF213 and NP through Co‐IP assay. Flag‐tagged NP and His‐tagged RNF213 plasmids were co‐expressed in HEK293T cells. The results showed that RNF213 obviously interacted with NP (Figure 9D). Endogenous RNF213 readily interacted with NP after PR8 virus infection for 12 h in A549 cells (Figure 9E). Moreover, the direct interaction between RNF213 and NP was confirmed (Figure 9F). In addition, MG132 treatment completely reversed the decrease in NP activity, whereas CQ treatment did not (Figure 9G), indicating that RNF213 regulates NP protein stability through the ubiquitin‐proteasome system. To further examine whether RNF213 can catalyze the K48‐linked polyubiquitination of IAV NP, HEK293T cells were co‐transfected with expression plasmids encoding Flag‐tagged NP, HA‐ubiquitin‐K48, and HA‐ubiquitin‐K63 together with empty vector or His‐tagged RNF213. The results demonstrated that RNF213 significantly enhanced the K48‐linked ubiquitination of NP, while it had no observable impact on the K63‐linked ubiquitination of NP (Figure 9H). Taken together, our data indicate that RNF213 promotes K48‐linked polyubiquitination and degradation of IAV NP.

## 4 Discussion

IAV remains a leading cause of respiratory infections worldwide, posing a persistent threat to global public health and resulting in substantial economic losses. The virus's capacity for rapid evolution, driven by genetic mutation and reassortment, often leads to mismatches with existing vaccines and the emergence of strains resistant to current antiviral therapies. Consequently, the development of effective strategies to combat influenza virus infections remains a critical public health priority.

Upon infection, the innate immune system serves as the first line of defense against IAV. A key component of this response is the production of IFNs, which are triggered by PAMPs [31]. IFNs bind to their corresponding cell surface receptors, activating the JAK‐STAT signaling pathway. This pathway rapidly induces the expression of a broad spectrum of ISGs, which play a vital role in combating and eliminating viral invaders. A deeper understanding of the antiviral mechanisms of ISGs in IAV infection is essential for the rational design of novel antiviral therapies. In this study, we discovered that the expression of RNF213, a protein with antiviral properties, can be induced by both IFN signaling and IAV infection. RNF213 showed significant inhibitory effects on influenza virus replication both in vitro and in vivo. These results are consistent with previous research showing that RNF213 is upregulated in response to H5N1 virus infection [32] and is induced by IFN‐β in conditions such as intracellular bacterial infections [19], multiple sclerosis (MS) [33], and moyamoya disease (MMD) [34]. In addition, IFN‐γ has been shown to induce RNF213 during *Toxoplasma gondii* infection [22, 35], further supporting its role as an ISG. Mechanistically, RNF213 was found to suppress IAV replication in human cell lines and mouse models by interacting with MDA5, thereby enhancing IFN production. These results highlight the pivotal role of RNF213 in the innate immune response to influenza virus and provide new insights into its potential as a target for antiviral drug development.

Upon infection with the influenza virus, viral RNA is recognized by intracellular sensors such as RIG‐I or MDA5. This recognition triggers the aggregation of MAVS, which subsequently recruits and activates the kinases TBK1 and IKKε. These kinases ultimately phosphorylate and activate the transcription factors IRF3/7, leading to the production of type I IFN [36]. In this study, we show that overexpression of RNF213 in cells significantly increases IFN‐β expression and reduces viral replication, whereas knockdown or knockout of RNF213 attenuates these effects, suggesting RNF213 plays a critical role as an antiviral factor during IAV infection. Our findings further demonstrate that RNF213 overexpression selectively elevated the protein levels of RIG‐I, MDA5, and IRF3, whereas the levels of MAVS and TBK1 remained unaffected. This selective stabilization implies that RNF213, functioning through its E3 ubiquitin ligase activity, directly or indirectly interacts with and stabilizes these specific signaling components. We propose that this occurs by antagonizing their constitutive or signal‐induced degradation pathways. In contrast, core scaffolding proteins such as MAVS, which assembles into large, stable prion‐like aggregates upon activation, and the central kinase TBK1 likely possess intrinsic stability or are subject to degradation mechanisms that are not principally governed by RNF213. Notably, the subcellular localization of RNF213 itself is context‐dependent. While it can localize to lipid droplets under conditions of lipid overload to regulate lipid metabolism [27], it exhibits a diffuse cytoplasmic distribution in unstimulated cells, consistent with its role in cytosolic innate immune signaling against pathogens [20, 37]. This dynamic localization likely allows RNF213 to perform distinct cellular functions. Anyway, our findings uncover a novel mechanism by which RNF213 modulates the RLR‐mediated antiviral innate immune response and advance our understanding of its role in innate immunity.

Polyubiquitination of MDA5, in particular by K63‐linked chains, is essential for its antiviral activity and induction of type I IFN production [38]. Although MDA5 is recognized as a key player in the cellular defense against IAV [39, 40], the specific mechanisms underlying its activation and the post‐translational modifications (PTMs) involved remain less understood compared to those of RIG‐I. Identification of positive regulators of MDA5 is therefore crucial to elucidate its role in antiviral immunity. Our research shows that RNF213 positively regulates MDA5‐mediated IFN production by promoting K63‐linked polyubiquitination at Lys137 and Lys743 of MDA5. Mutation at these sites significantly impairs MDA5 polyubiquitination and its antiviral functions, highlighting the importance of RNF213 in modulating MDA5 activity. These results suggest that RNF213 enhances virus‐induced IFN signaling by facilitating K63‐linked polyubiquitination of MDA5. However, the precise mechanism by which RNF213 activates MDA5 requires further investigation. In particular, our findings do not exclude the possibility that other E3 ubiquitin ligases, such as members of the TRIM family, may also contribute to MDA5 polyubiquitination in a coordinated manner. This notion is supported by our observation that the RNF213 ΔNE construct, which lacks the E3 ligase domain, still induced a residual level of MDA5 ubiquitination, suggesting the involvement of additional cellular E3 ligases. A previous report demonstrated that the E3 ligase TRIM65 also catalyzes K63‐linked ubiquitination of MDA5 at K743 to facilitate its filament formation [41], suggesting a potentially complex regulatory network for MDA5 activity. In addition, ISG15 conjugation, a process critical for MDA5‐mediated antiviral IFN responses [42], has been linked to RNF213, which can interact with ISG15 [19]. Here, we further demonstrate that RNF213 promotes ISG15 conjugation to MDA5. Interestingly, the MDA5‐K137/743R mutant, which is refractory to RNF213‐mediated ubiquitination and signaling potentiation, retained wild‐type levels of ISGylation that were still enhanced by RNF213. This uncoupling indicates that K137 and K743 are specific hubs for ubiquitin modification but are dispensable for ISGylation. While ISGylation has been shown to stabilize MDA5 and promote its signaling [42], our findings establish that RNF213 orchestrates a dual modification program on MDA5. Collectively, our findings establish that RNF213 acts as a multifaceted regulator that coordinately enhances both K63‐linked ubiquitination and ISGylation of MDA5 to drive optimal antiviral signaling. The functional significance of the interaction between RNF213 and ISG15 in the activation of MDA5 during IAV infection remains an open question and warrants further investigation. Future studies utilizing immunopurification of MDA5 followed by mass spectrometric analysis under conditions of RNF213 overexpression or viral infection will be invaluable to definitively map the specific lysine residues modified by RNF213 and to potentially identify additional sites involved in the fine‐tuning of MDA5 activity. Future studies dissecting whether these modifications occur sequentially, synergistically, or on distinct MDA5 subpopulations will yield deeper insights into the precision of innate immune regulation. Taken together, these findings deepen our understanding of the intricate regulatory networks that govern the innate immune response to viral infection and highlight RNF213 as a key player in antiviral defense mechanisms.

Degradation of free NP restricts the translocation of NP to the nucleus, thereby inhibiting influenza virus replication. Several ISGs can target the viral NP protein. By way of example, the interaction between TRIM22 and viral NP leads to its polyubiquitination and proteasome‐dependent degradation, thereby inhibiting IAV replication [43]. TRIM41 directly binds the viral NP and targets it for ubiquitination and proteasomal degradation, thereby limiting IAV infection [44]. TRIM25 binds to vRNPs in the nuclei of infected cells and inhibits the onset of influenza viral RNA chain elongation [45]. Additionally, TRIM14 interacts with the NP protein and acts as an intermediate mediator to induce proteasomal degradation [46]. However, using the ubiquitome proteomics technology in combination with infection or IFN‐inducing conditions, it cannot be excluded that part of the GlyGly(K) sites are ISG15 conjugation sites, since ISG15 leaves a similar GlyGly remnant on modified K residues [47]. Although multiple ISGs have been identified, many mechanisms of their actions on the influenza virus remain unclear. Further study of ISGs contributes to a deeper understanding of the host anti‐influenza virus mechanism.

**FIGURE 10:**
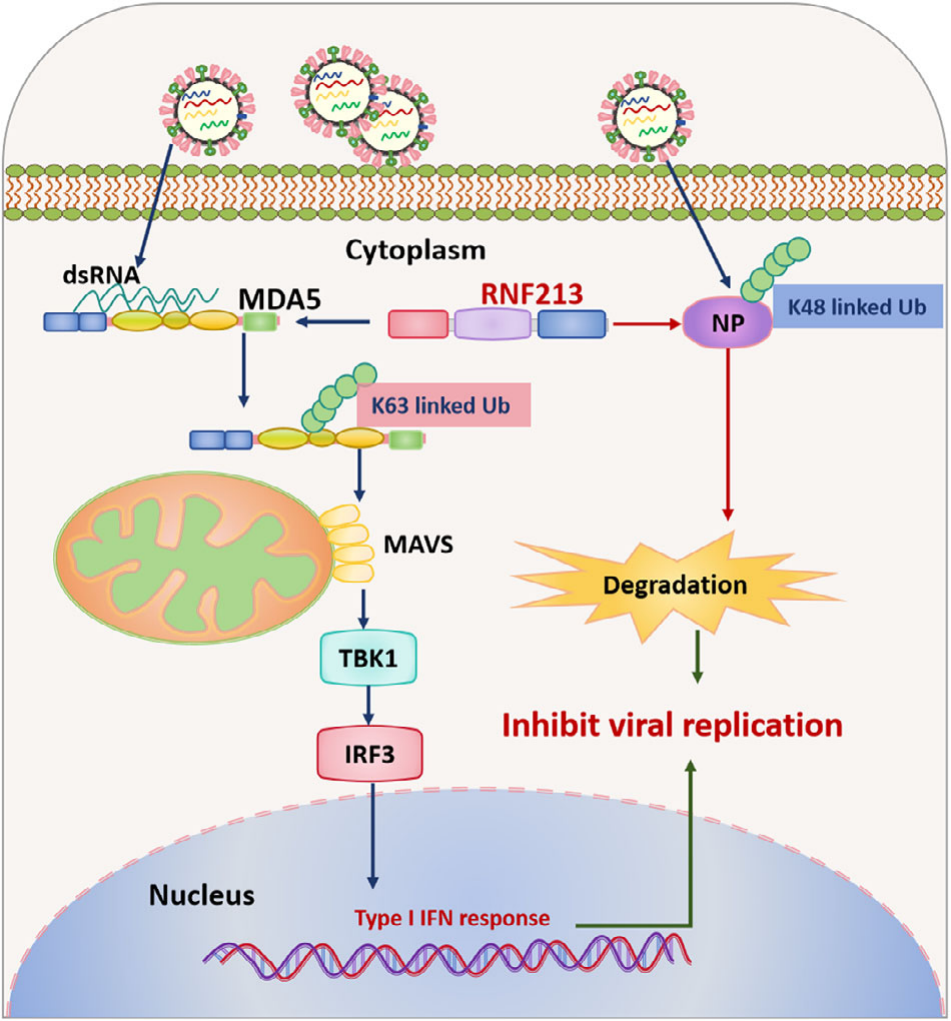
Schematic model of the proposed role of RNF213 in antiviral immunity against influenza virus infection. Briefly, influenza virus infection upregulates RNF213 expression. RNF213 promotes K63‐linked polyubiquitination of MDA5 and enhances IFN production. Moreover, RNF213 mediates K48‐linked polyubiquitination and proteasomal degradation of viral NP.

Collectively, our study reveals that RNF213 orchestrates a dual‐layer defense against IAV infection. First, it functions as a positive regulator of innate immunity by catalyzing K63‐linked ubiquitination of MDA5, thereby amplifying the type I IFN response and establishing an antiviral state. Second, it acts as a direct viral restriction factor by mediating K48‐linked ubiquitination and proteasomal degradation of the viral NP protein. This bifunctional activity positions RNF213 as a key node that bridges broad‐spectrum immune signaling with precise viral targeting, offering a potent and coordinated defense strategy. Based on these data, we propose a working model to illustrate how RNF213 regulates MDA5‐mediated type I IFN production and NP degradation in IAV infection (Figure 10). Future studies will be essential to investigate the effect of RNF213 deficiency or overexpression on the replication of multiple influenza virus types. This discovery indicates that RNF213 is a novel ISG in antiviral innate immune response to IAV infection and opens new avenues for the development of targeted antiviral therapies.

## Author Contributions

F.W. and H.L. conceived and designed the experiments; H.L. and Y.Z. performed the experiments; J.W., Q.Z., and H.S. analyzed the data; H.L., Y.Z., and F.W. wrote the manuscript.

## Conflicts of Interest

The authors declare no conflict of interest.

## Supporting information




**Supporting File**: advs74901‐sup‐0001‐SuppMat.docx.

**Supporting File**: advs74901‐sup‐0002‐Blots.zip.

**Supporting File**: advs74901‐sup‐0003‐TableS1.xlsx.

**Supporting File**: advs74901‐sup‐0004‐TableS2.docx.

**Supporting File**: advs74901‐sup‐0005‐TableS3.docx.

**Supporting File**: advs74901‐sup‐0006‐TableS4.xlsx.

## Data Availability

The data that support the findings of this study are openly available in ProteomeXchange Consortium at https://proteomecentral.proteomexchange.org, reference number 64429.
