# Supplementary material for: RNF213 Is an Interferon‐Stimulated Gene That Targets Influenza A Virus NP and Activates MDA5 to Restrict Infection
Source: Adv Sci (Weinh). 2026 Mar 18;13(31):e11012. doi: 10.1002/advs.202511012 (PMC13588011; doi:10.1002/advs.202511012)
Supplement: Supplementary file 1 [file ADVS-13-e11012-s001.docx]

**Supplemental Information**

**Figure S1. High-throughput gene expression analysis of IFN-α or IFN-γ treatment.**

(A) Volcano plots showing differentially expressed genes for IFN-α-treated A549 cells. The threshold was set as follows: *p* <0.05 and |log2FC|>1. FC: fold change. Green and red dots indicate significantly downregulated and upregulated genes, respectively. (B) Heatmap of differentially expressed patterns of the overlap genes in IFN-α-treated A549 cells. Row represents the overlap genes, and each column corresponds to one sample replicate. (C) Volcano plots showing differentially expressed genes for IFN-γ-treated A549 cells. The threshold was set as follows: *p* <0.05 and |log2FC|>1. FC: fold change. Green and red dots indicate significantly downregulated and upregulated genes, respectively. (D) Heatmap of differentially expressed patterns of the overlap genes in IFN-γ-treated A549 cells. Row represents the overlap genes, and each column corresponds to one sample replicate. (E) GO biological processes of differentially expressed overlap in IFN-α-treated A549 cells. The vertical axis shows the functional classification, and the horizontal axis shows the GeneRatio. (F) KEGG pathways of differentially expressed overlap genes in IFN-α-treated A549 cells. The vertical axis shows the number of genes, and the horizontal axis shows the GeneRatio. (G) Top 30 biological processes associated with commonly differentially expressed genes in IFN-γ-treated A549 cells. (H) KEGG pathways of differentially expressed overlap genes in IFN-γ-treated. The vertical axis shows the number of genes, and the horizontal axis shows the GeneRatio.

**Figure S2. Knockdown efficiency of RNF213-targeting siRNAs in HEK293T cells.**

Serum-starved HEK293T cells were transfected with siRNAs (either negative controls or RNF213-targeting siRNAs), and the RNF213 protein level was examined 48 h later by western blotting. β-actin is shown as a loading control. The representative results are shown.

**Figure S3. Genotyping strategy and results for RNF213 KO mice.**

(A) Schematic of the targeting strategy for generating RNF213 KO mice. (B) Representative agarose gel electrophoresis of PCR products from mouse tail genomic DNA. The complete primer sequences used are F1: 5’-GGGAAGCCTTGCCTGGGA GTGAG-3’; R1: 5’-GTCCTAAACTCCTGAGTGCTGTG-3’; F2: 5’-AAGTCAGAG GTTAGGGACATGGC-3’; R2: 5’-GTCCTAAACTCCTGAGTGCTGTG-3’. PCR was performed using Taq polymerase with an annealing temperature of 60.0°C. Expected product sizes: wild-type allele (641 bp), homozygous knockout allele (800 bp). Heterozygous mice show both bands. Genotypes are indicated above each lane (+/+, wild-type; –/–, homozygous knockout).

**Figure S4. RNF213 enhances ISGylation of MDA5 independently of K137 and K743.**

Analysis of MDA5 ISGylation in HEK293T cells co-transfected with HA-ISG15 and either wild-type (WT) Flag-MDA5 or the Flag-MDA5-K137/743R mutant, together with empty vector or His-RNF213 for 24 h. Immunoprecipitation was performed with anti-Flag antibody. β-actin is shown as a loading control in whole-cell lysates (Input). All panels are representative of at least three independent experiments.

**Figure S5. Quantitative analysis of key western blot experiments from three independent biological replicates.**

For each experiment, data from three independent replicates are shown. Left panels: Uncropped western blot images showing the relevant lanes for Replicates 1, 2, and 3. Right panels: Quantitative densitometry analysis presented as bar/line graphs (mean ± SEM, n=3). Statistical significance was assessed as described in the Methods. (A) Data corresponding to Fig. 5A: Effect of increasing RNF213 dosage on RIG-I protein levels. (B) Data corresponding to Fig. 5B: Effect of increasing RNF213 dosage on MDA5 protein levels. (C) Data corresponding to Fig. 5C: Effect of increasing RNF213 dosage on MAVS protein levels. (D) Data corresponding to Fig. 5D: Effect of increasing RNF213 dosage on TBK1 protein levels. (E) Data corresponding to Fig. 5E: Effect of increasing RNF213 dosage on IRF3 protein levels. (F) Data corresponding to Fig. 7B: Determination of MDA5 protein half-life following cycloheximide (CHX) treatment. (G) Data corresponding to Fig. 8F: Comparative analysis of ubiquitination levels for MDA5 wild-type and ubiquitination-site mutants.

**Supplementary Table 1. Up-regulated genes in A549 cells after both IFN-α and IFN-γ treatment for 6 h.**

**Supplementary Table 2. List of primer pairs used for q-PCR in this study.**

**Supplementary Table 3. Potential ubiquitination sites within MDA5.**

Potential ubiquitinated lysine residues within MDA5 were identified using the dbPTM, PhosphoSitePlus^®^, and PTMGPT2 models and were cross-referenced with published literature.

**Supplementary Table 4. Ubiquitination sites identified on viral proteins in lung tissues from PR8 virus-infected WT and RNF213 KO mice.**
