# Supplementary material for: RNF213 Is an Interferon‐Stimulated Gene That Targets Influenza A Virus NP and Activates MDA5 to Restrict Infection
Source: Adv Sci (Weinh). 2026 Mar 18;13(31):e11012. doi: 10.1002/advs.202511012 (PMC13588011; doi:10.1002/advs.202511012)
Supplement: Supplementary file 2 [file ADVS-13-e11012-s005.docx]

**Table S2. The qPCR primers sequence used in this study.**

| **Gene** | **Forward (5’-3’)** | **Reverse (5’-3’)** |
| --- | --- | --- |
| **GAPDH/Human** | AACTTTGGCATTGTGGAAGG | ACACATTGGGGGTAGGAACA |
| **GAPDH/Mouse** | ACAACTTTGGCATTGTGGAA | GATGCAGGGATGATGTTCTG |
| **IFN-β/Human** | CAACAAGTGTCTCCTCCAAAT | TCTCCTCAGGGATGTCAAAG |
| **IFN-β/Mouse** | TGAATGGAAAGATCAACCTCACCTA | CTCTTCTGCATCTTCTCCGTCA |
| **MDA5** | GCTGAAGTAGGAGTCAAAGCCC | CCACTGTGGTAGCGATAAGCAG |
| **CCL5** | CCTGCTGCTTTGCCTACATTGC | ACACACTTGGCGGTTCTTTCGG |
| **Viperin** | CCAGTGCAACTACAAATGCGGC | CGGTCTTGAAGAAATGGCTCTCC |
| **β-actin** | GCCGCCAGCTCACCAT | TCGATGGGGTACTTCAGGGT |
| **NP** | GGCCGTCATGGTGGCGAAT | CTCAATATGAGTGCAGACCGTGCT |
