# Supplementary material for: RNF213 Is an Interferon‐Stimulated Gene That Targets Influenza A Virus NP and Activates MDA5 to Restrict Infection
Source: Adv Sci (Weinh). 2026 Mar 18;13(31):e11012. doi: 10.1002/advs.202511012 (PMC13588011; doi:10.1002/advs.202511012)
Supplement: Supplementary file 3 [file ADVS-13-e11012-s004.zip › advs74901-sup-0002-Data/Uncropped WB strips.pdf]

Figure 1A

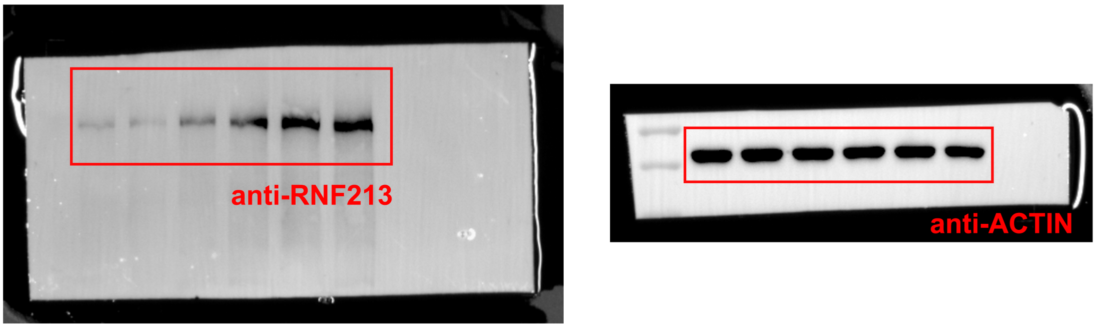

Figure 1B

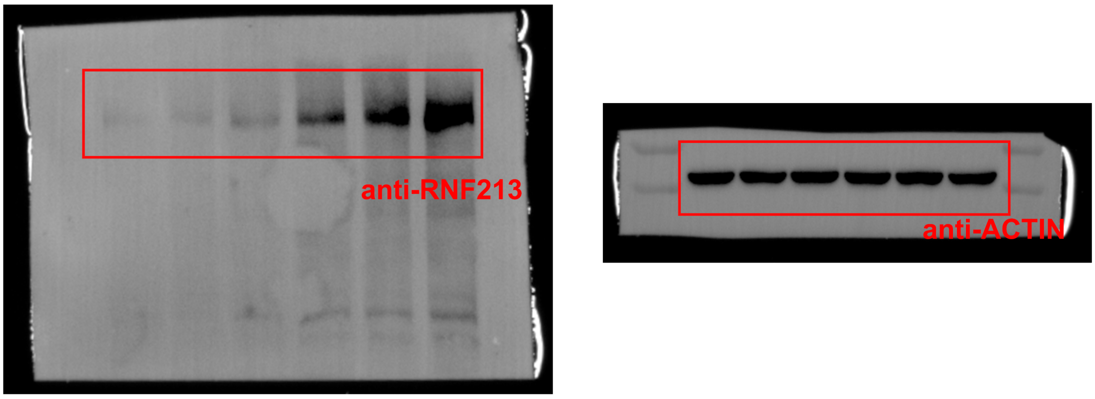

Figure 1C

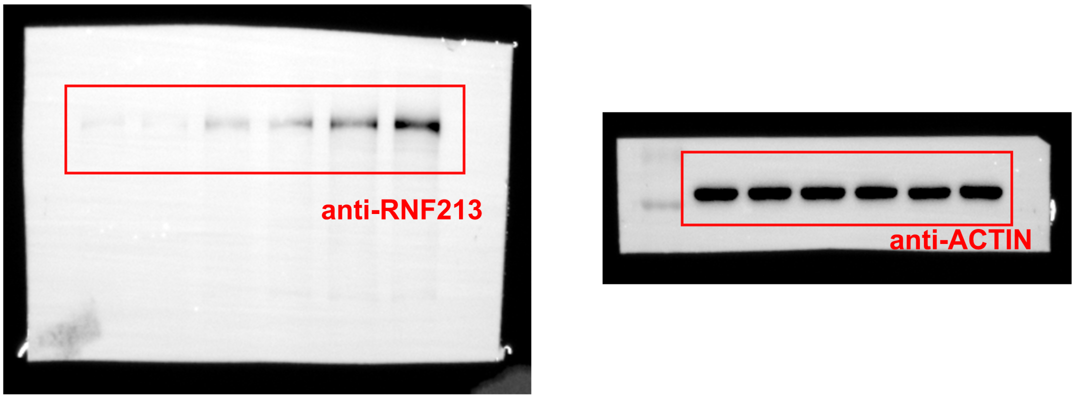

Figure 1D

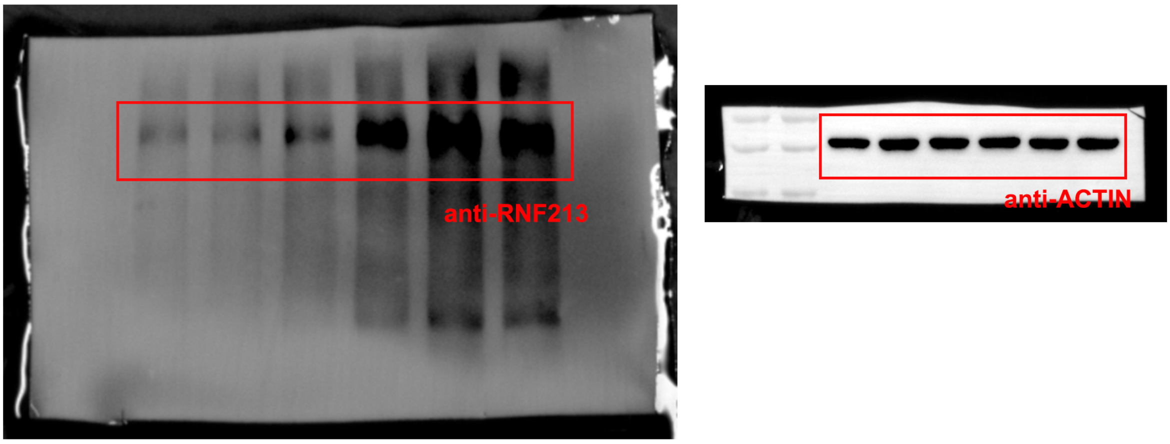

Figure 1E

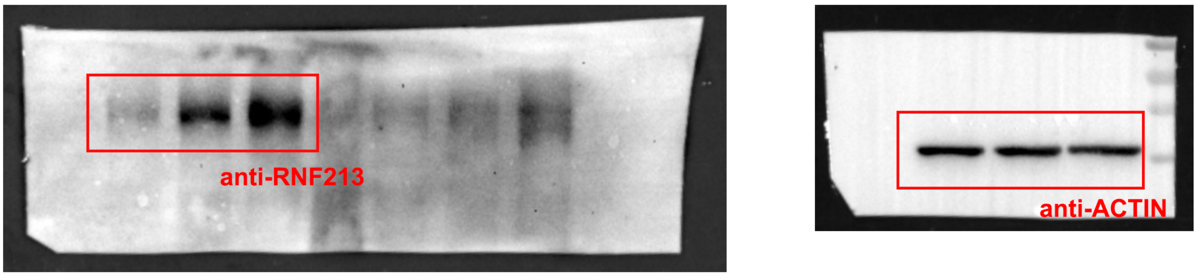

Figure 1F

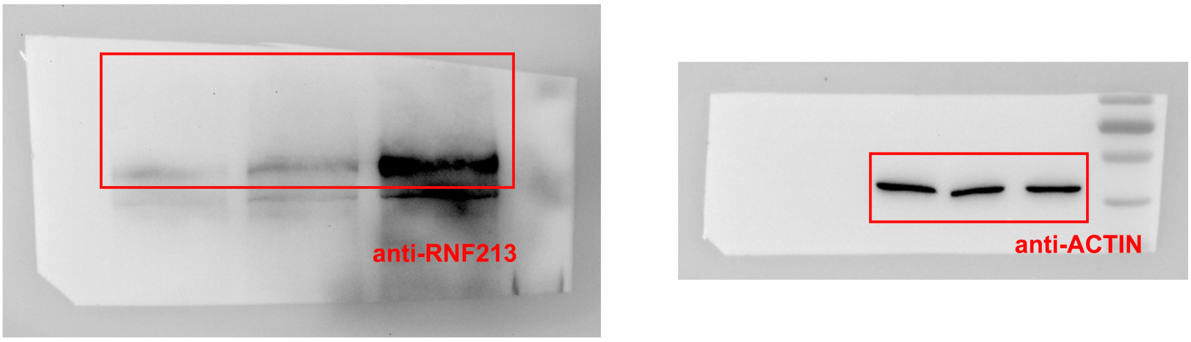

Figure 1G

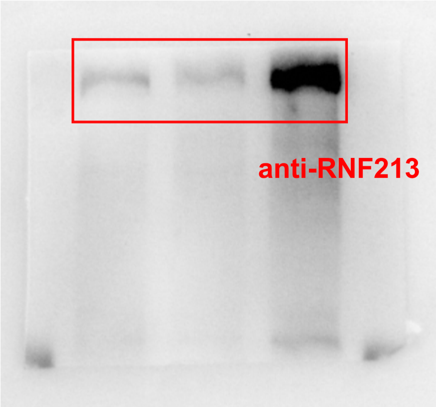

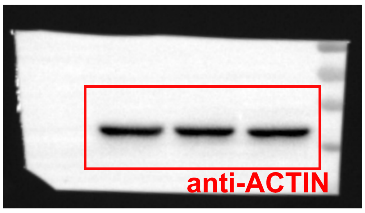

Figure 1H

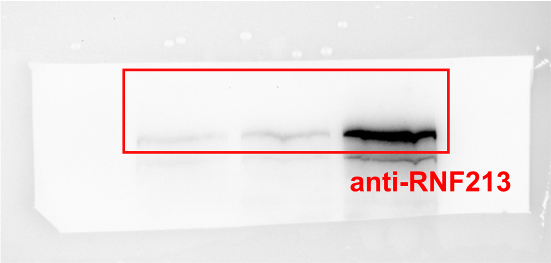

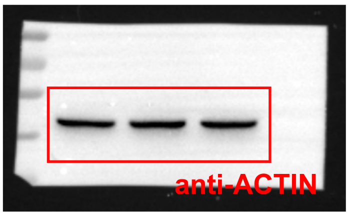

Figure 1I

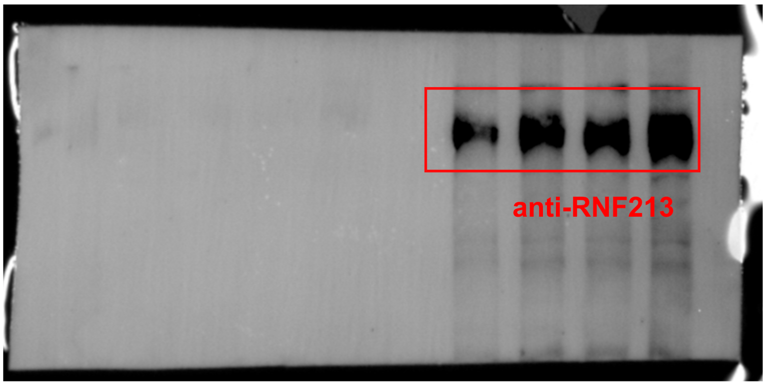

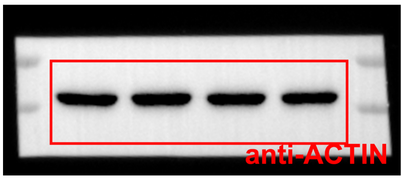

Figure 1J

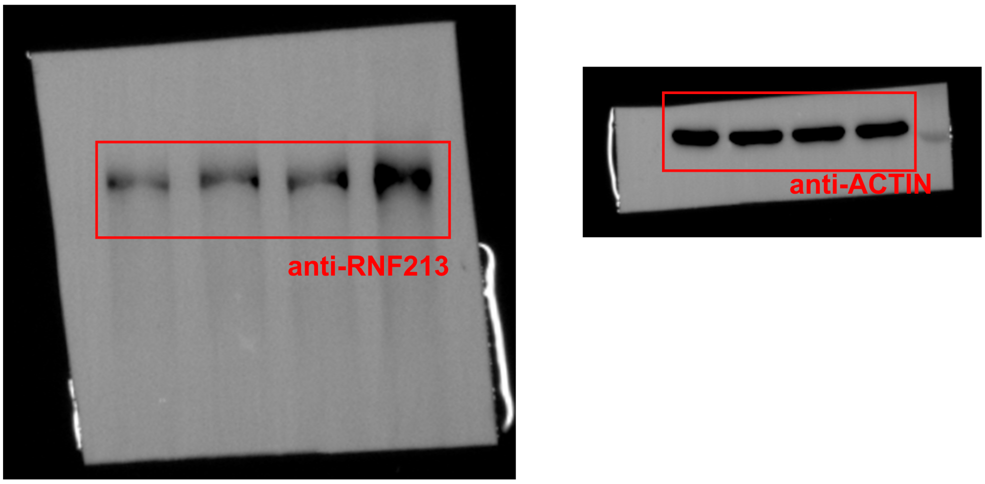

Figure 1K

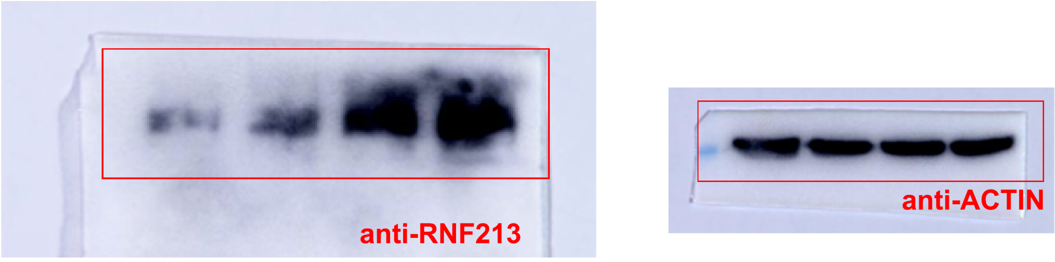

Figure 1L

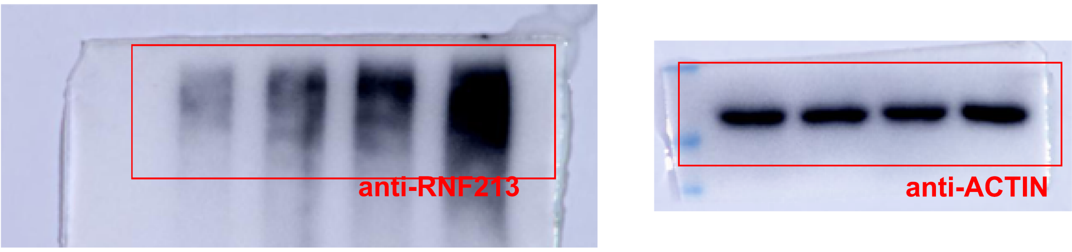

Figure 2C

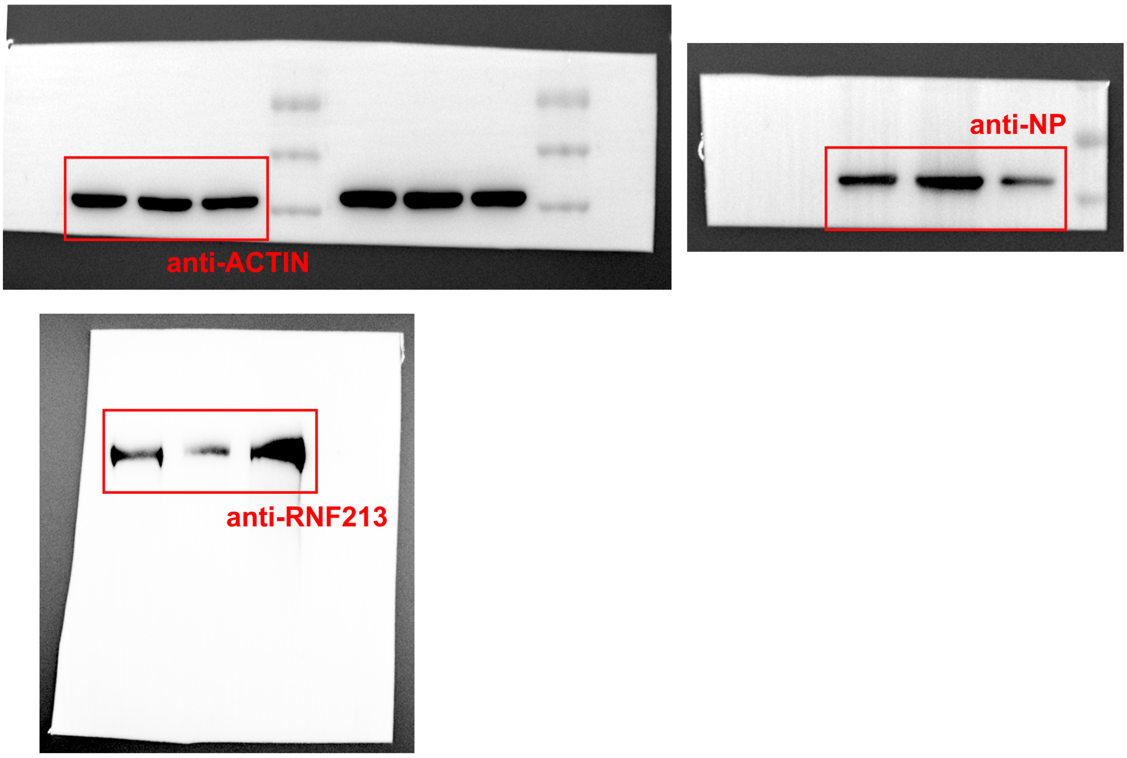

Figure 2D

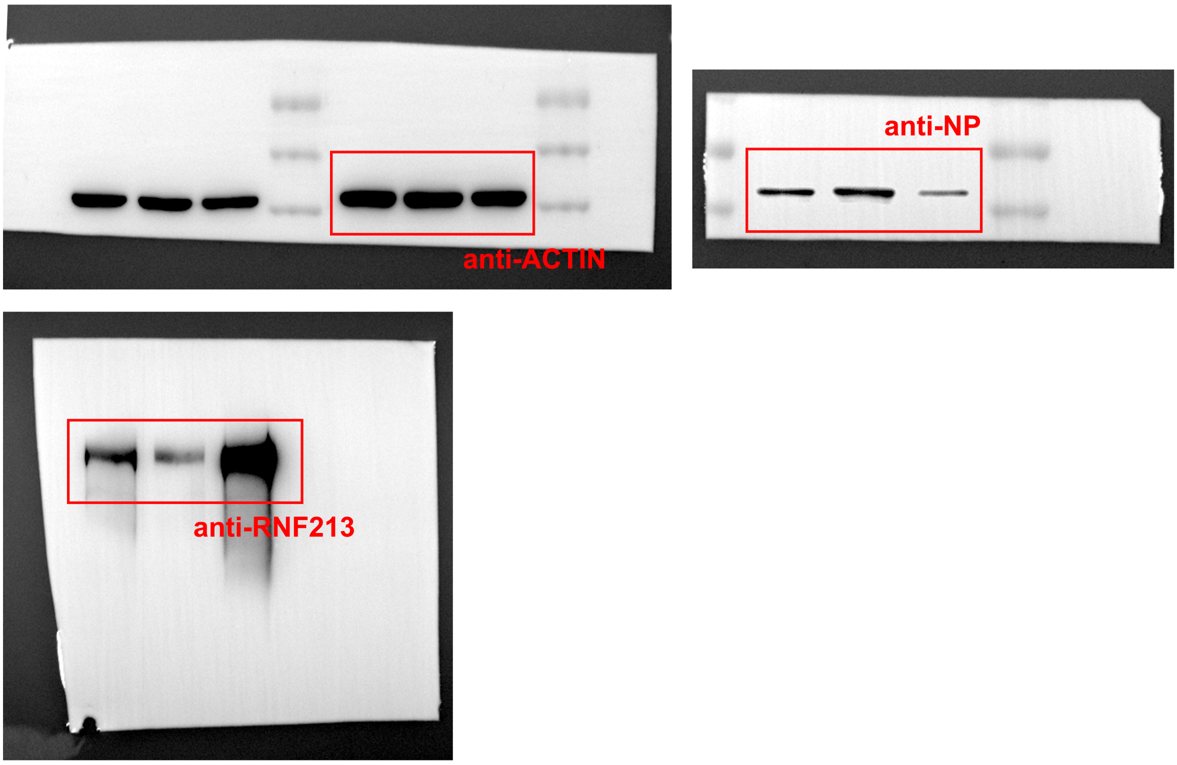

Figure 3F

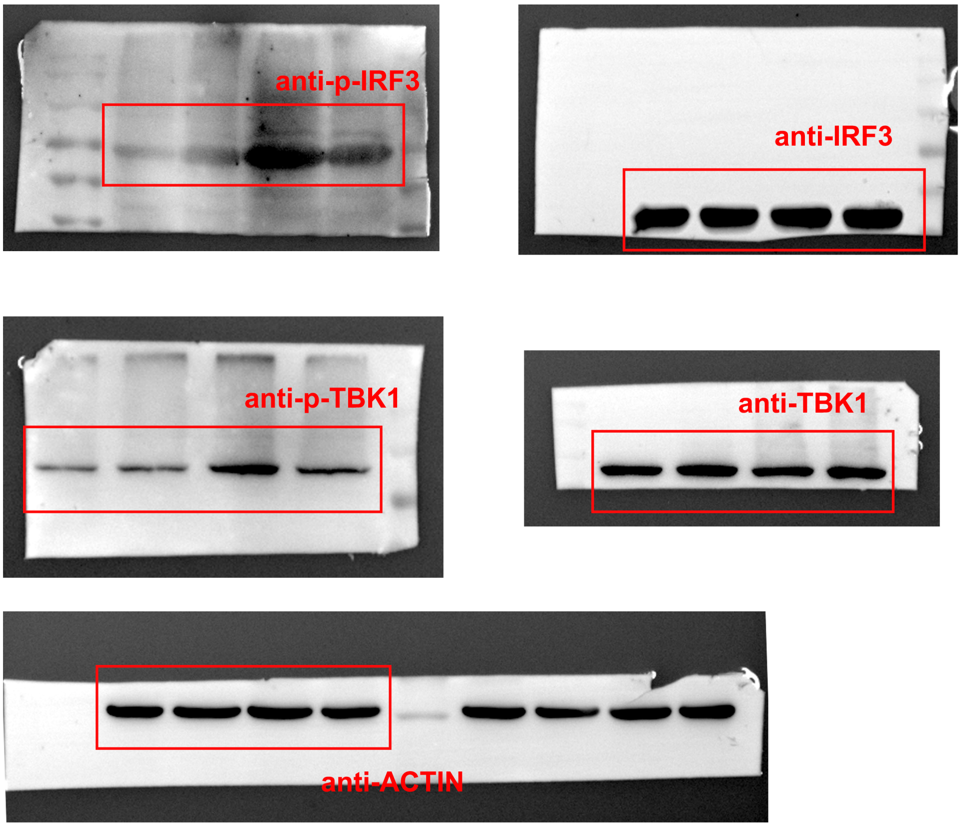

Figure 4B

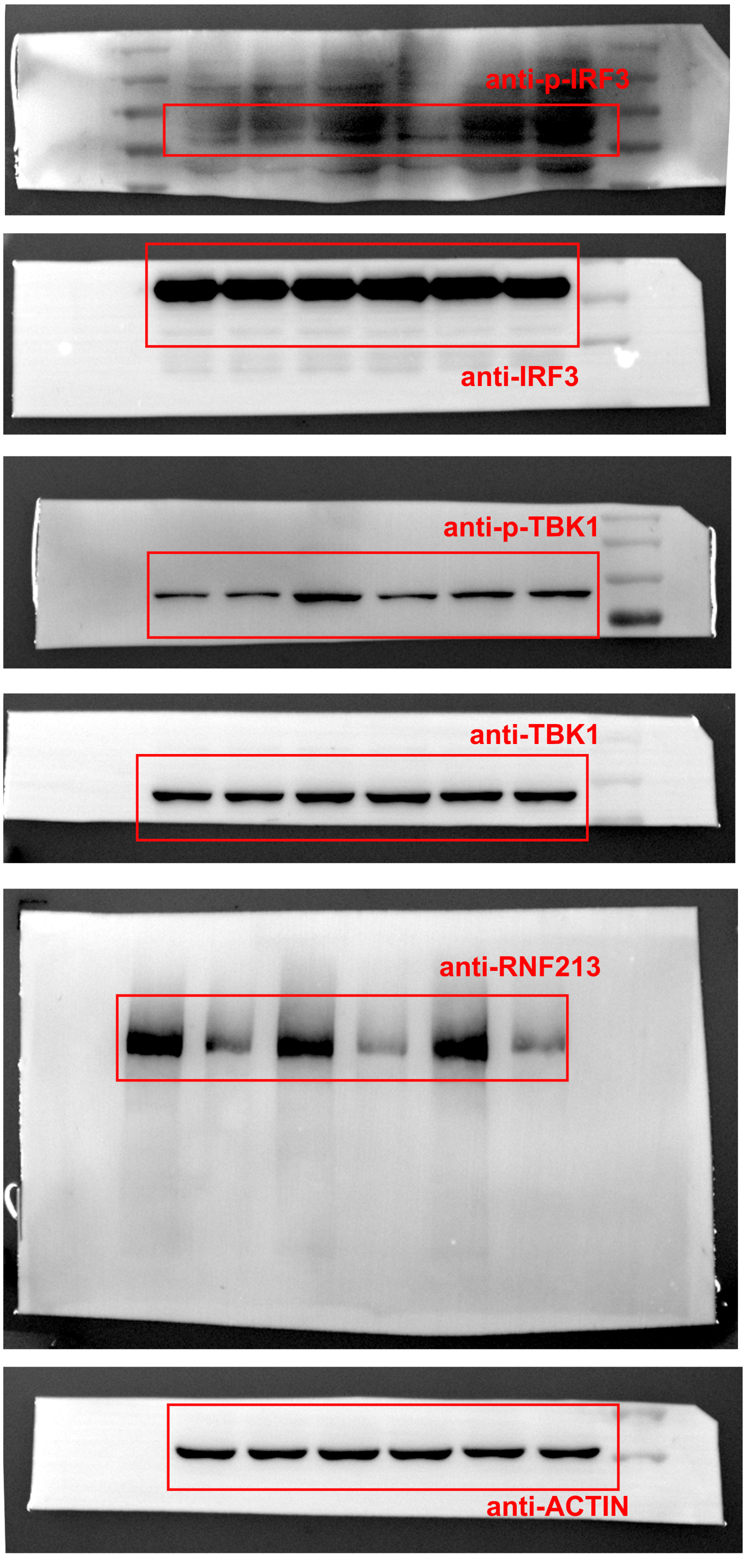
anti-p-IRF3
anti-IRF3
anti-p-TBK1
anti-TBK1
anti-RNF213
anti-ACTIN

Figure 5A

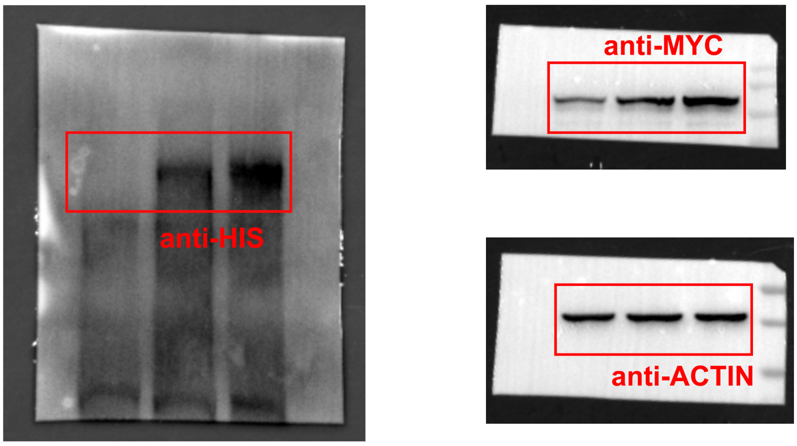

Figure 5B

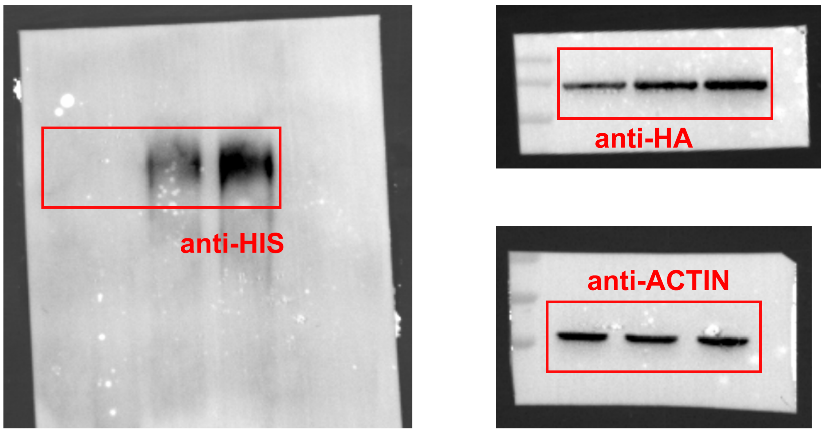

Figure 5C

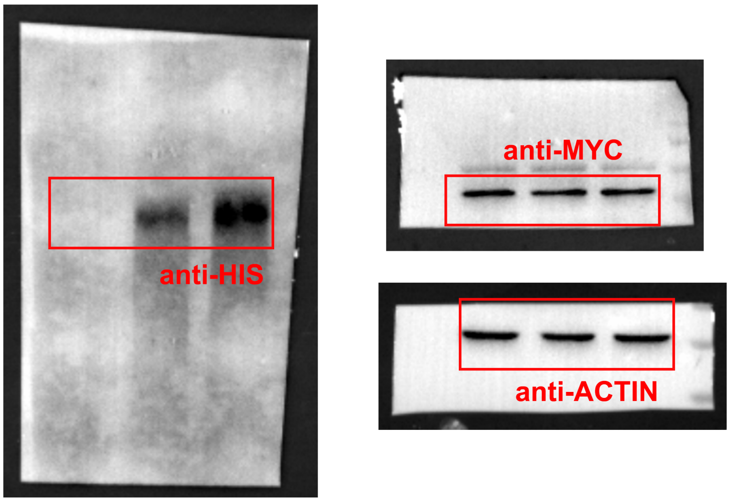

Figure 5D

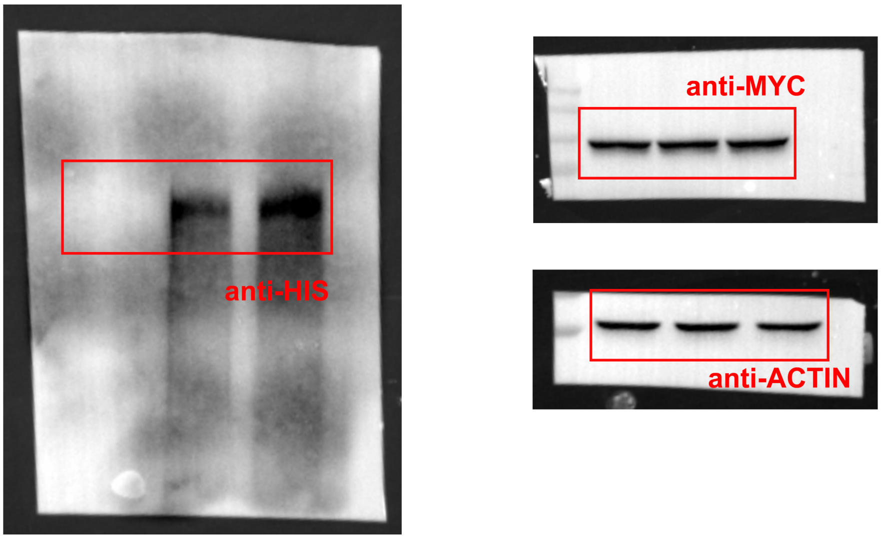

Figure 5E

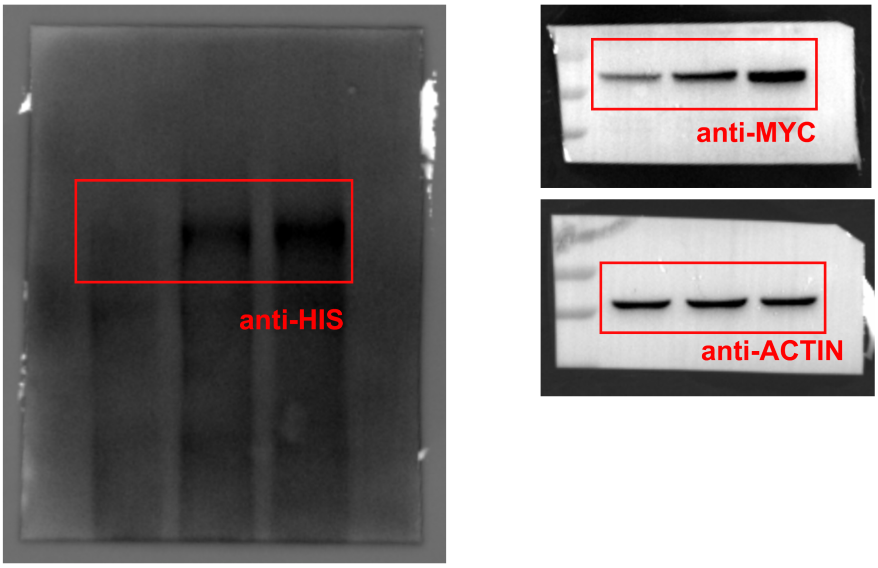

Figure 5F

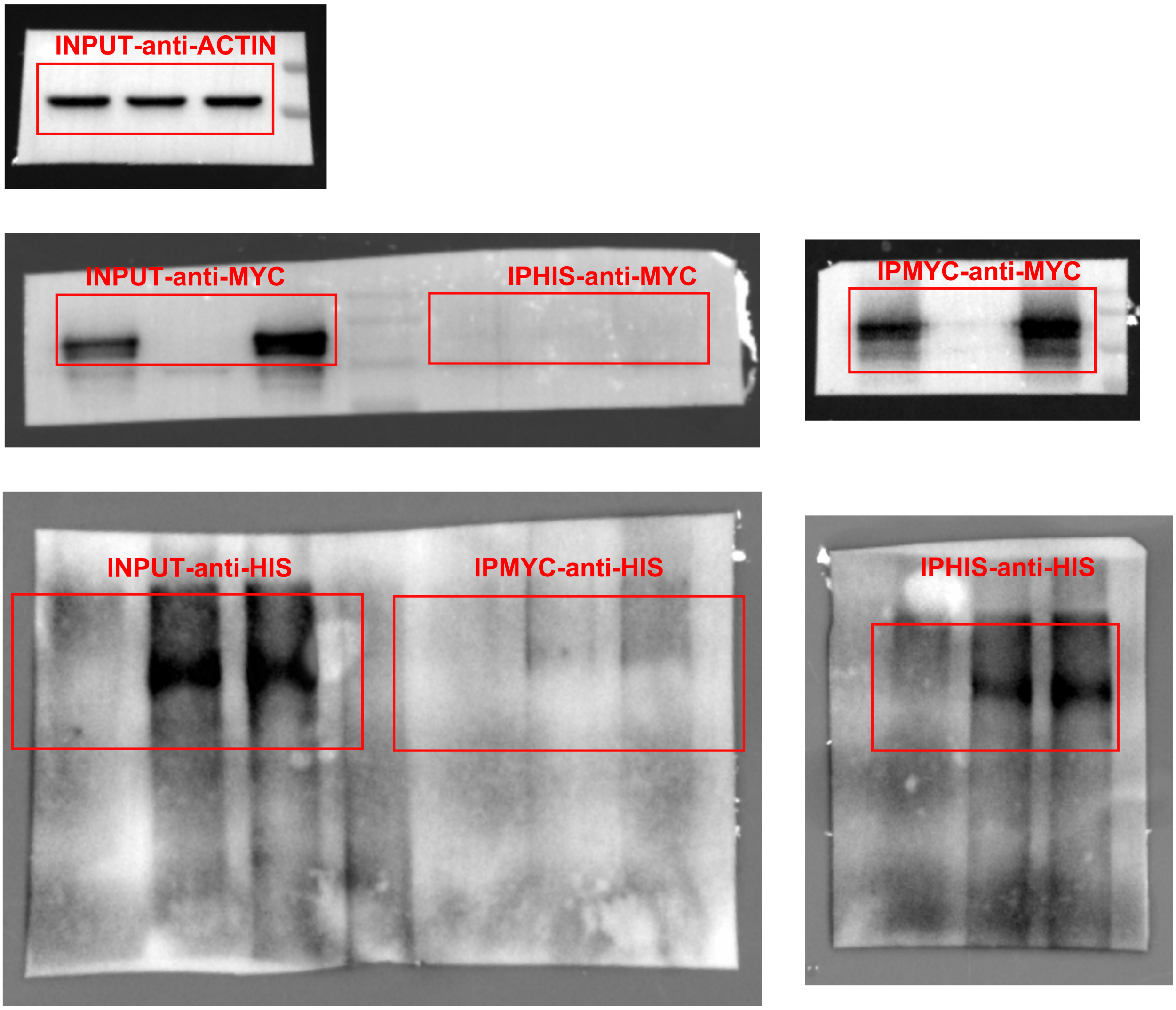
INPUT-anti-ACTIN
INPUT-anti-MYC
IPHIS-anti-MYC
IPMYC-anti-MYC
INPUT-anti-HIS
IPMYC-anti-HIS
IPHIS-anti-HIS

Figure 5G

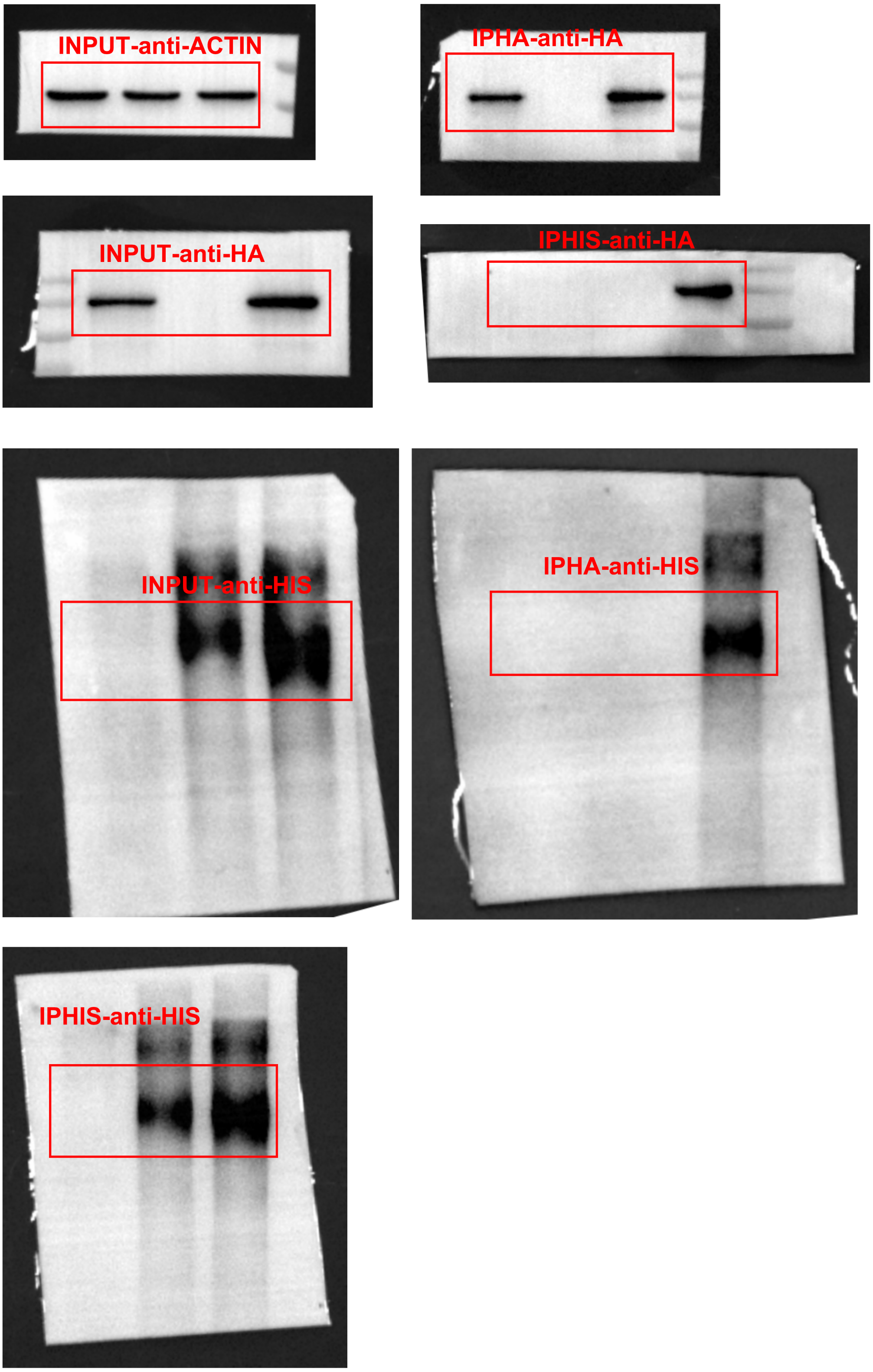
INPUT-anti-ACTIN
IPHA-anti-HA
INPUT-anti-HA
IPHIS-anti-HA
INPUT-anti-HIS
IPHA-anti-HIS
IPHIS-anti-HIS

Figure 5H

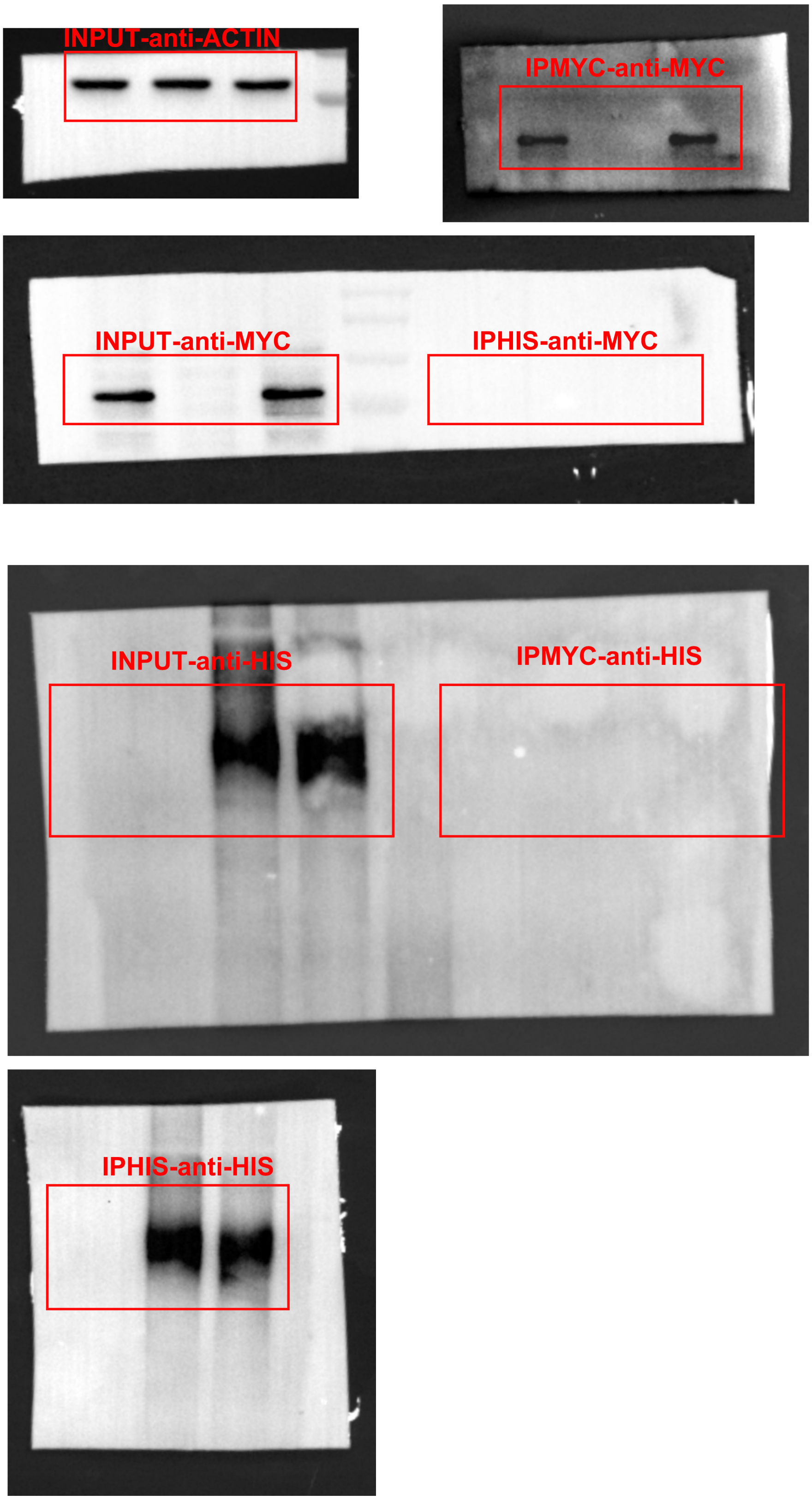
INPUT-anti-ACTIN
IPMYC-anti-MYC
INPUT-anti-MYC
IPHIS-anti-MYC
INPUT-anti-HIS
IPMYC-anti-HIS
IPHIS-anti-HIS

Figure 5I

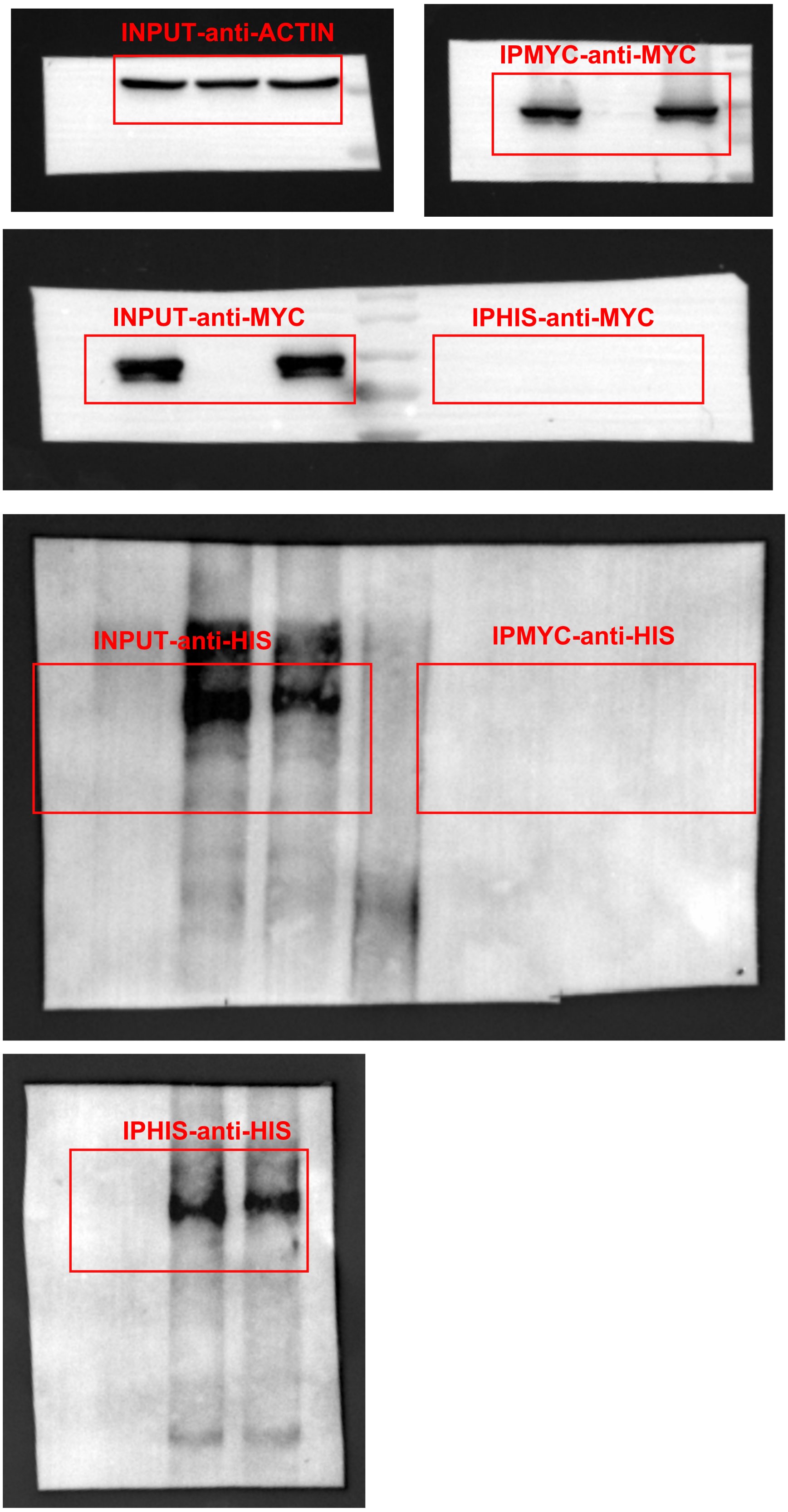
INPUT-anti-ACTIN
IPMYC-anti-MYC
INPUT-anti-MYC
IPHIS-anti-MYC
INPUT-anti-HIS
IPMYC-anti-HIS
IPHIS-anti-HIS

Figure 5J

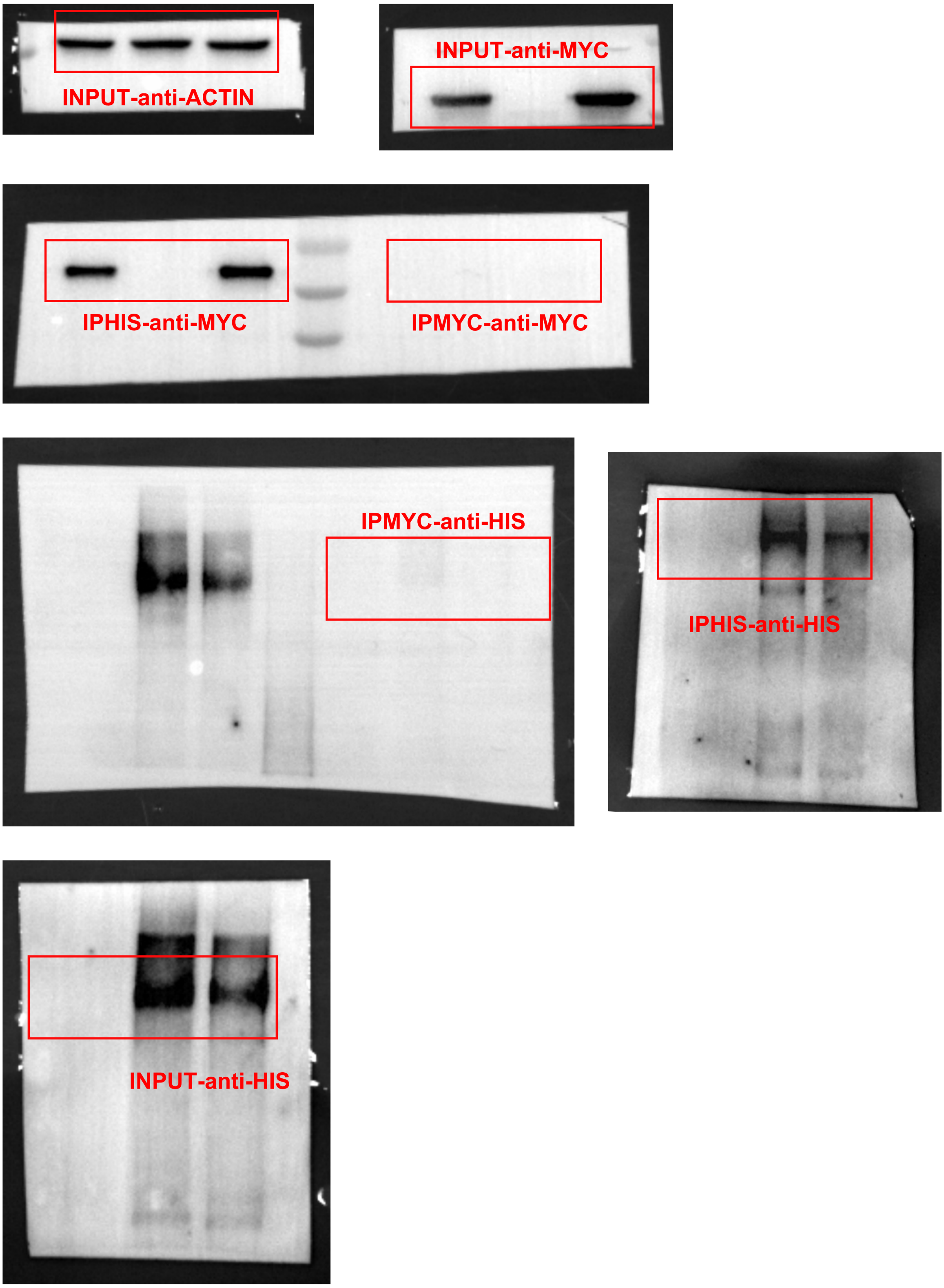
INPUT-anti-ACTIN
INPUT-anti-MYC
IPHIS-anti-MYC
IPMYC-anti-MYC
IPMYC-anti-HIS
IPHIS-anti-HIS
INPUT-anti-HIS

Figure 5K

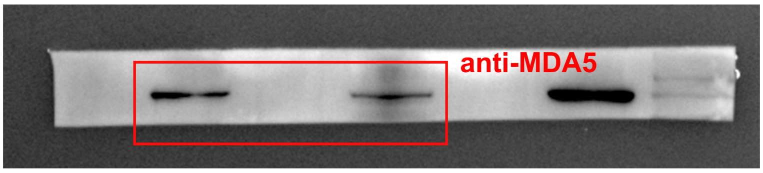

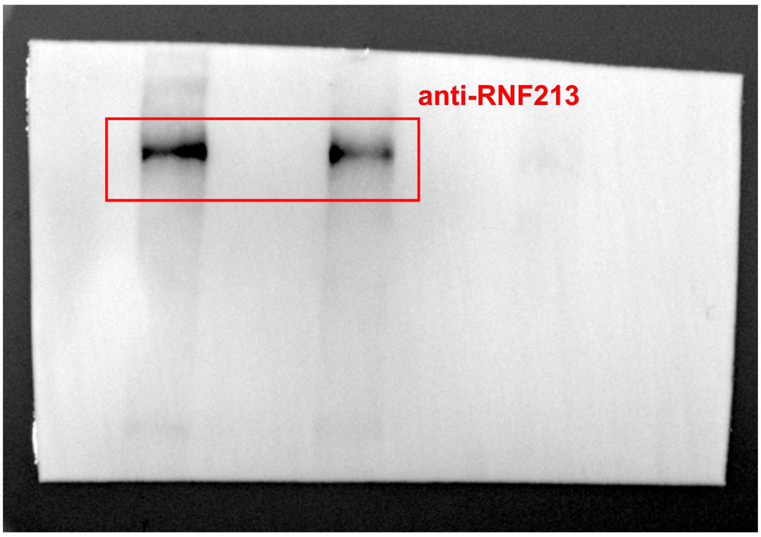

Figure 5L

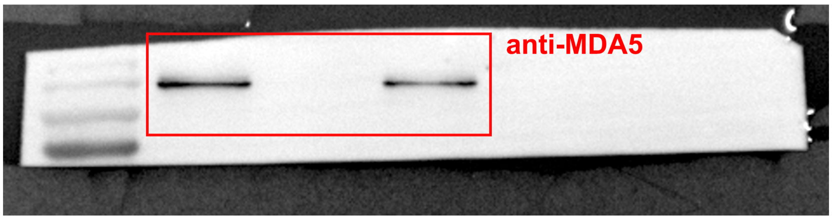

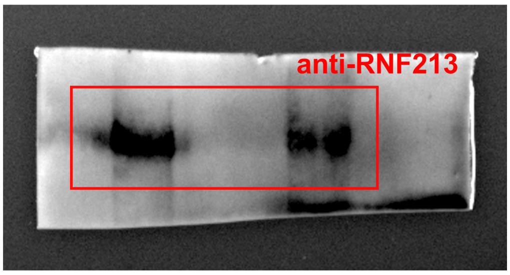

Figure 5M

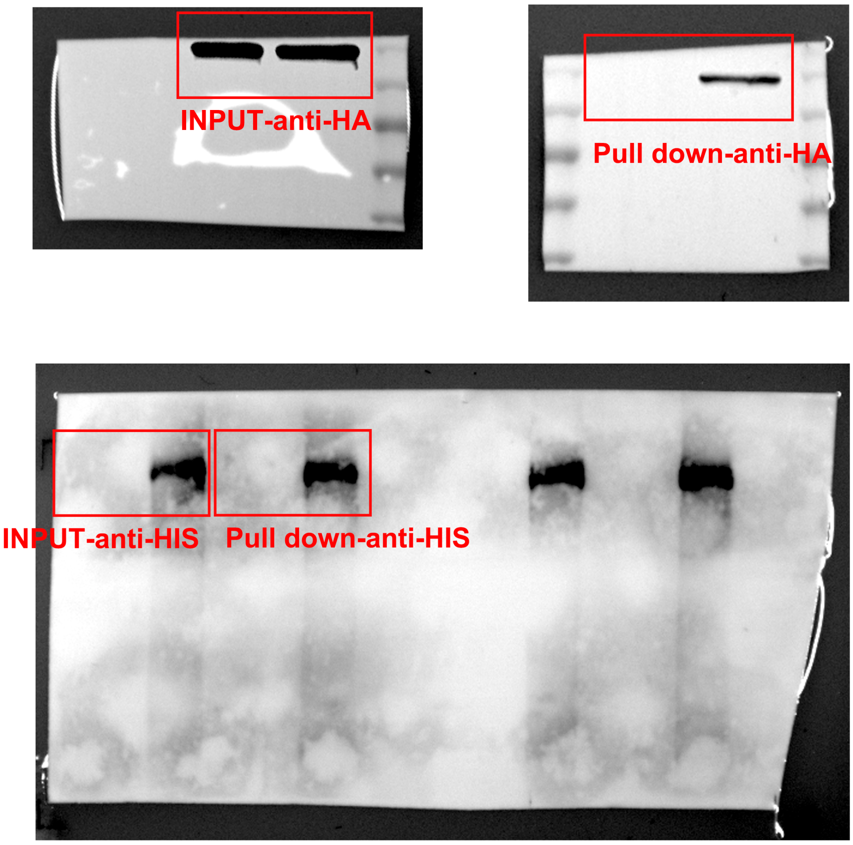
INPUT-anti-HA
Pull down-anti-HA
INPUT-anti-HIS
Pull down-anti-HIS

Figure 5P

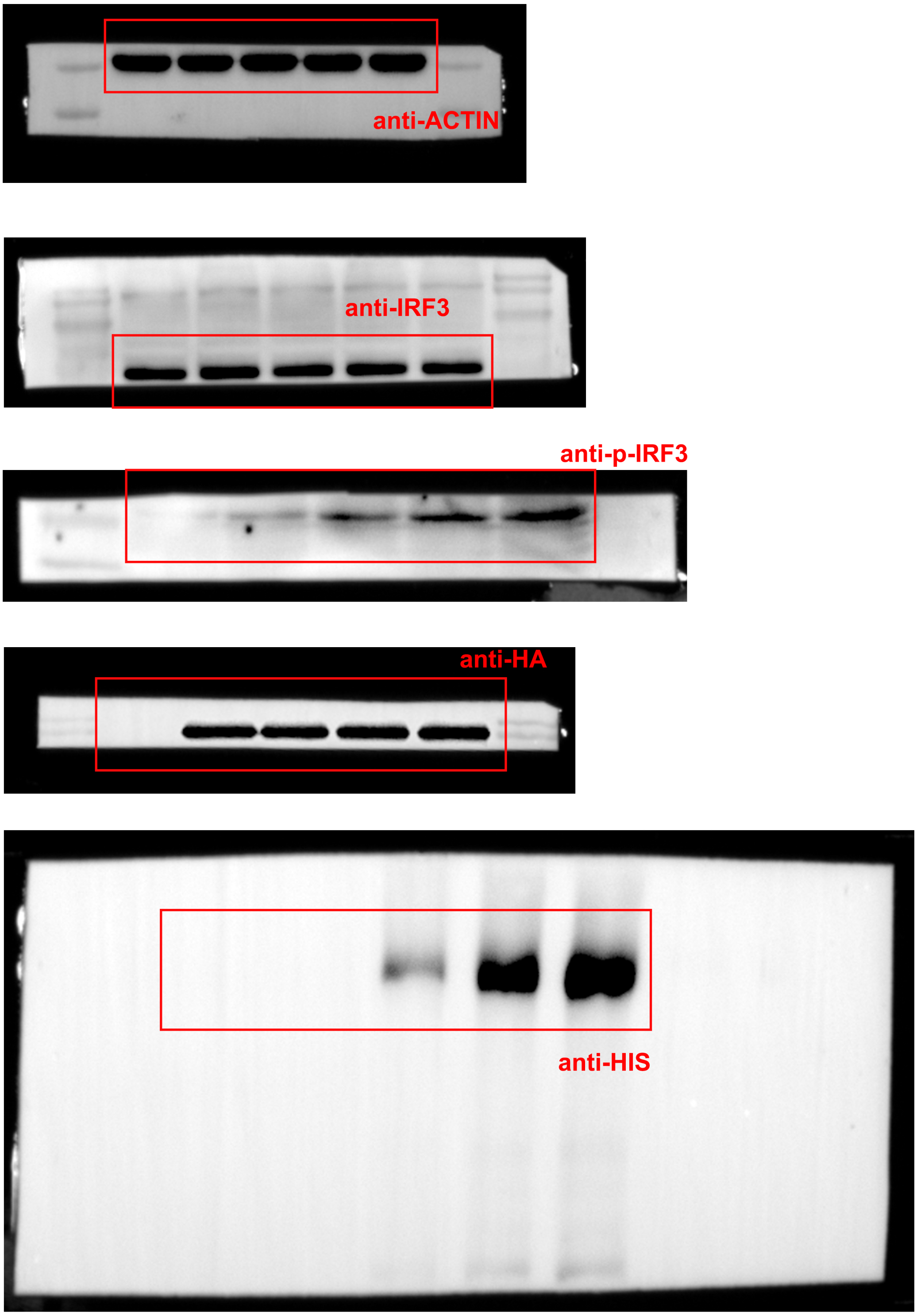

Figure 6C

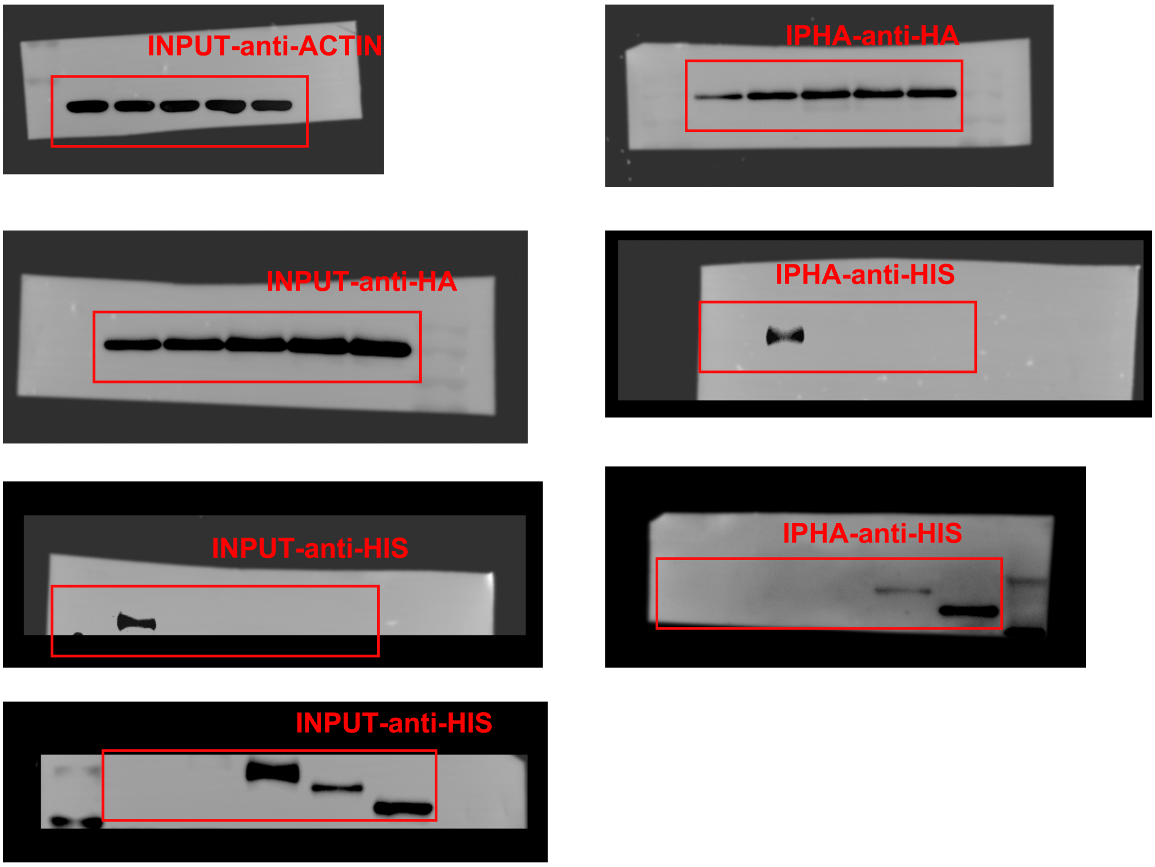
INPUT-anti-ACTIN
IPHA-anti-HA
INPUT-anti-HA
IPHA-anti-HIS
INPUT-anti-HIS
IPHA-anti-HIS
INPUT-anti-HIS

Figure 6E

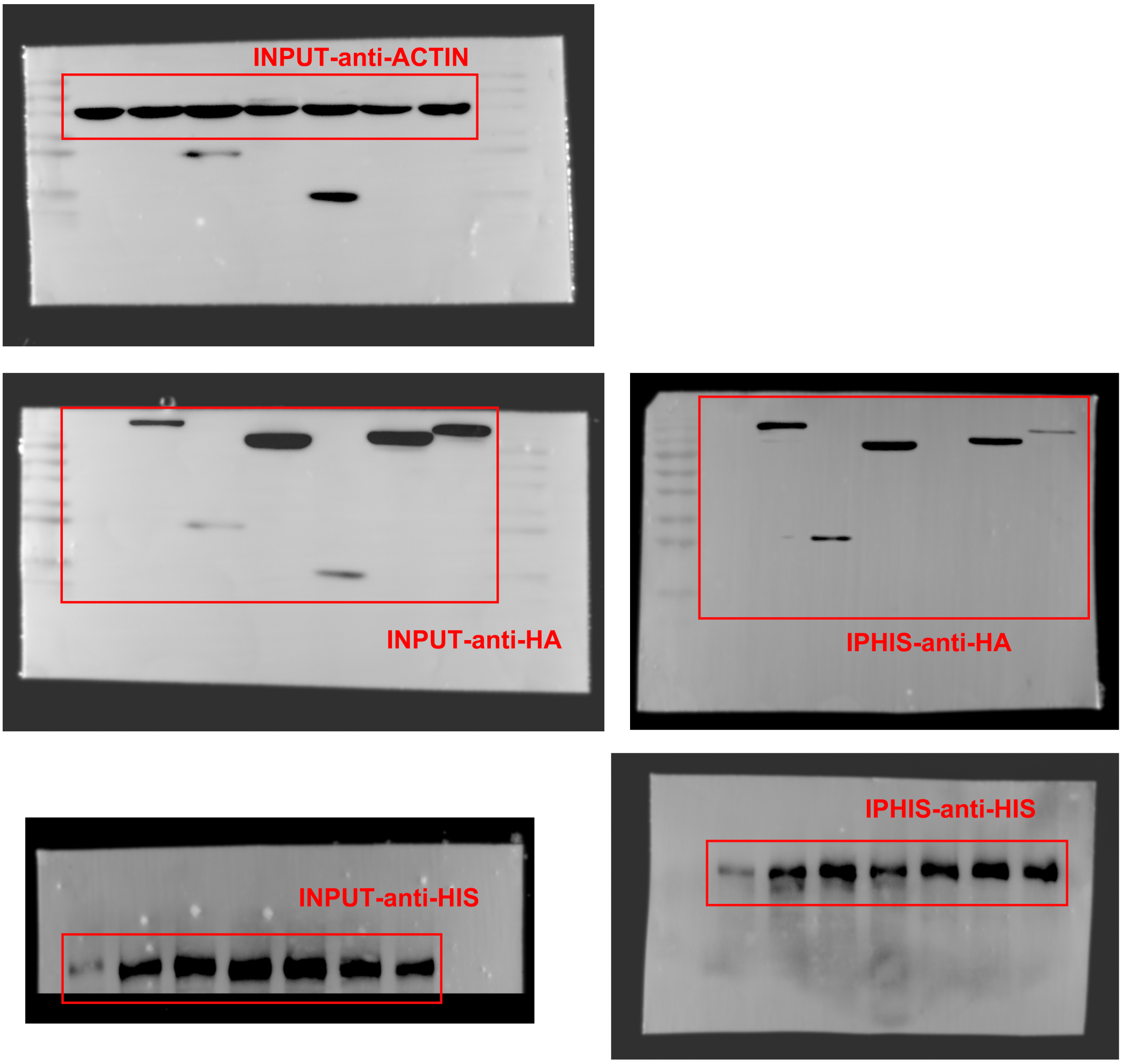
INPUT-anti-ACTIN
INPUT-anti-HA
IPHIS-anti-HA
INPUT-anti-HIS
IPHIS-anti-HIS

Figure 6G

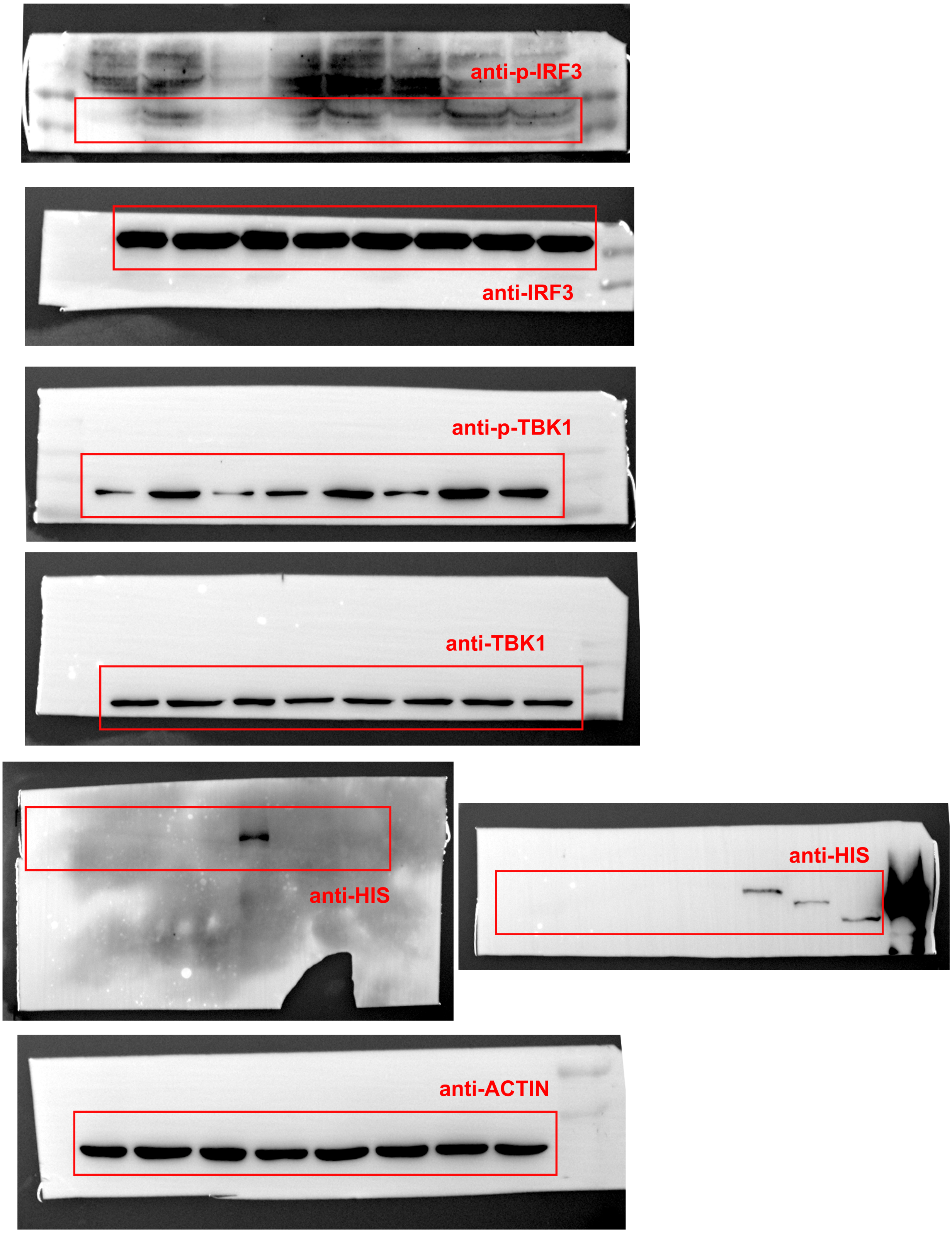
anti-p-IRF3
anti-IRF3
anti-p-TBK1
anti-TBK1
anti-HIS
anti-HIS
anti-ACTIN

Figure 7B

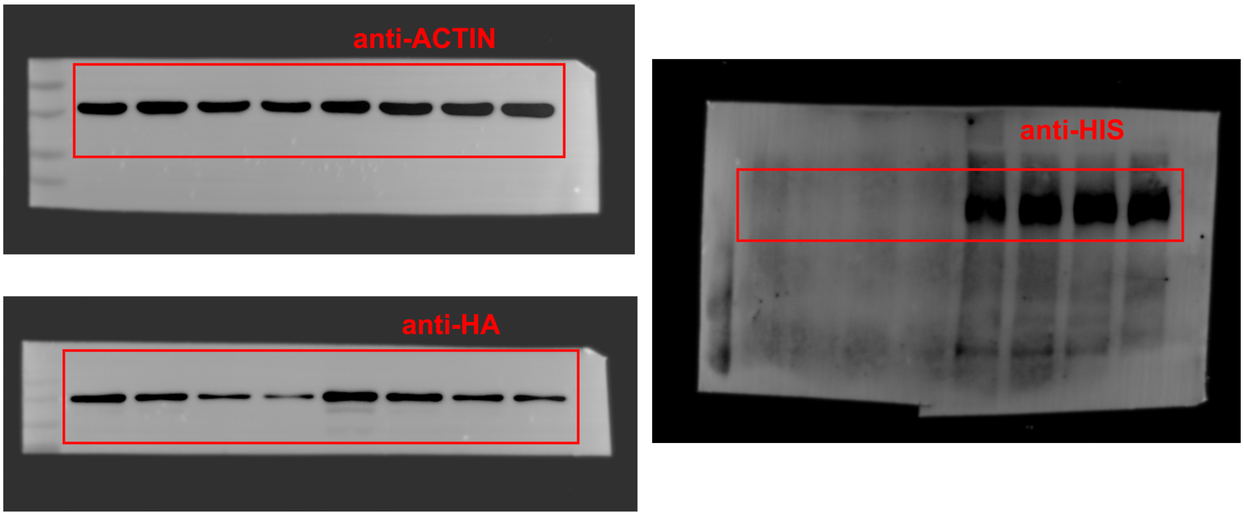

Figure 7C

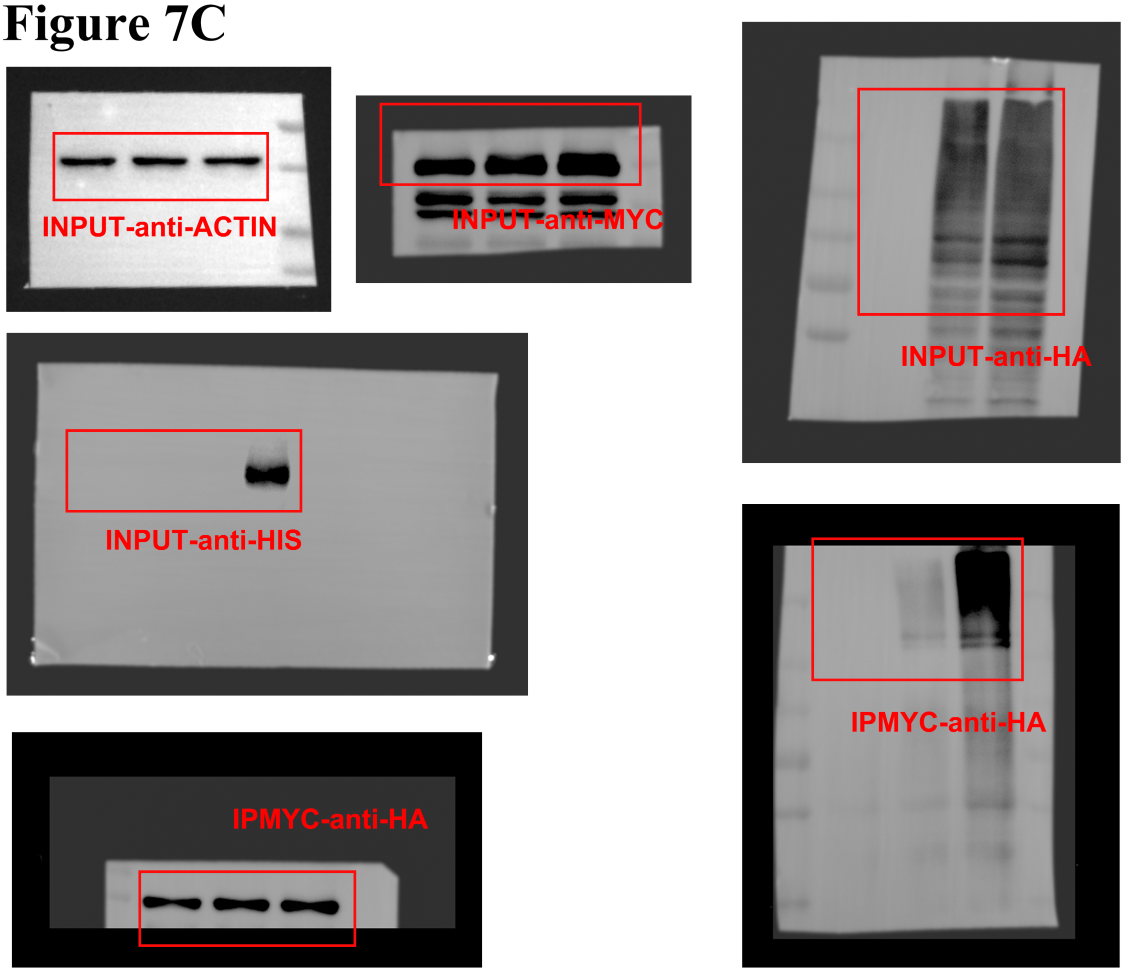

Figure 7D

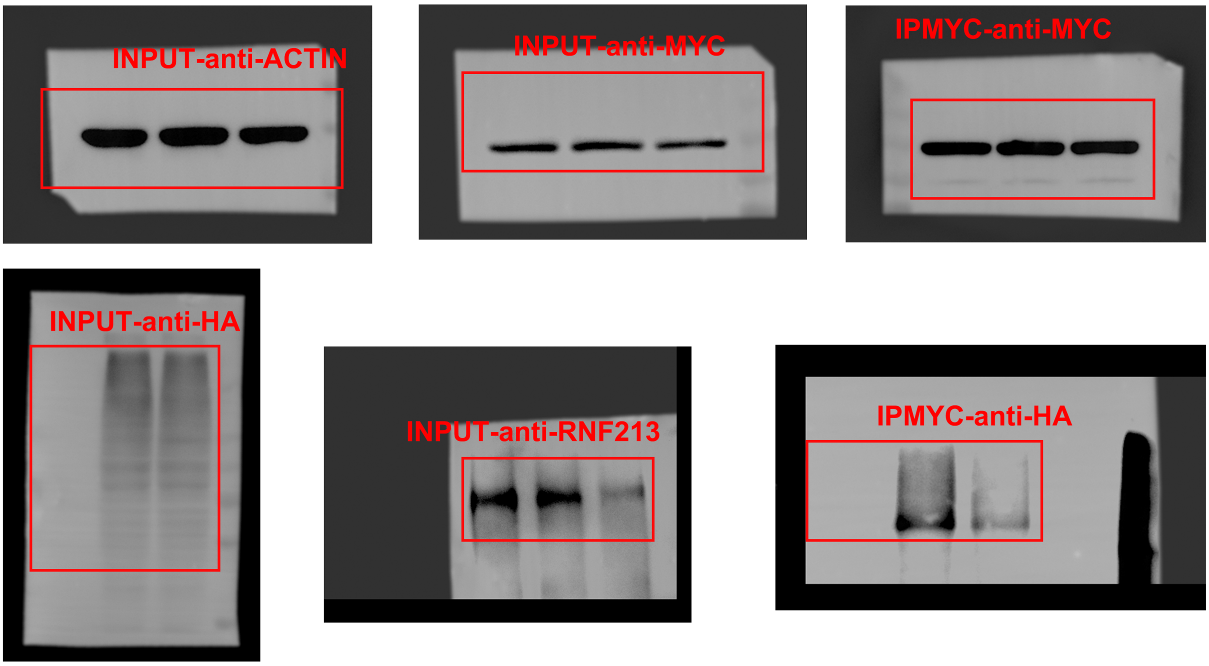

Figure 7E

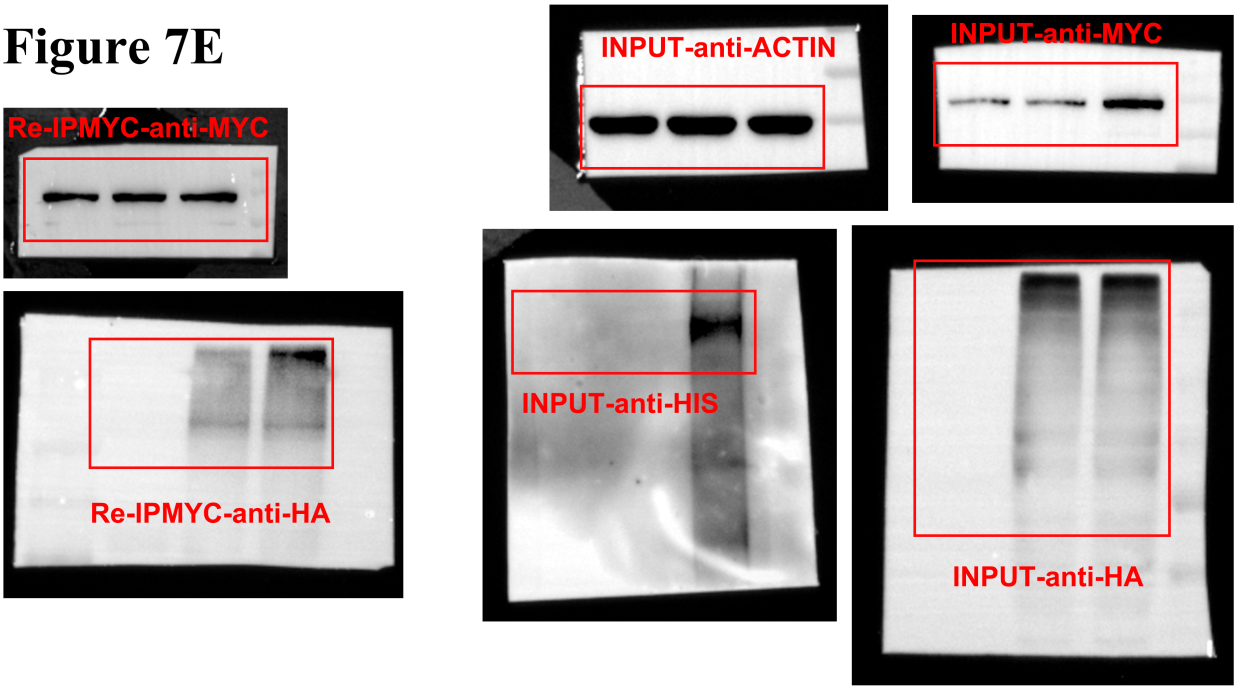

Figure 7F

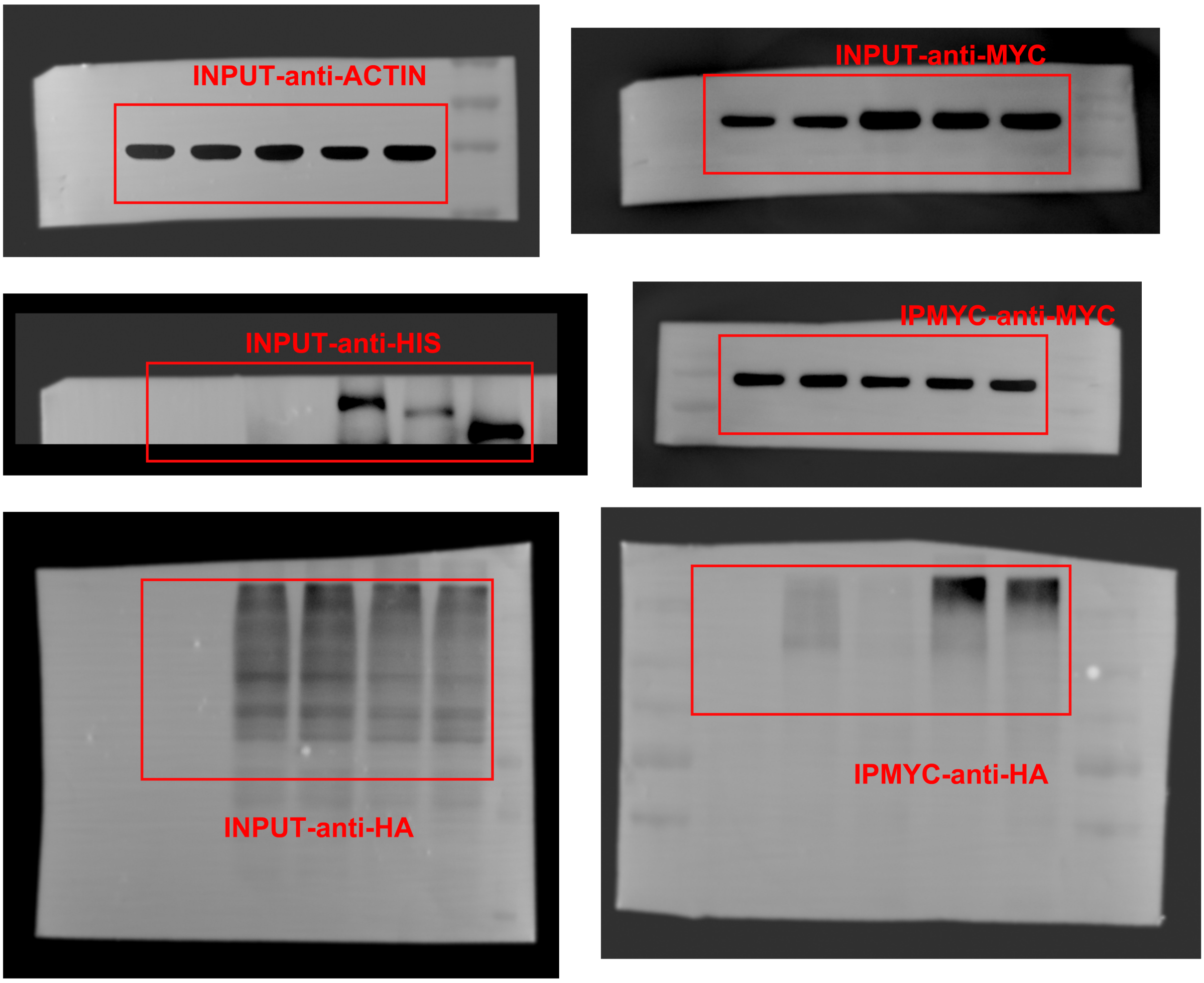
INPUT-anti-ACTIN
INPUT-anti-MYC
INPUT-anti-HIS
IPMYC-anti-MYC
INPUT-anti-HA
IPMYC-anti-HA

Figure 7G

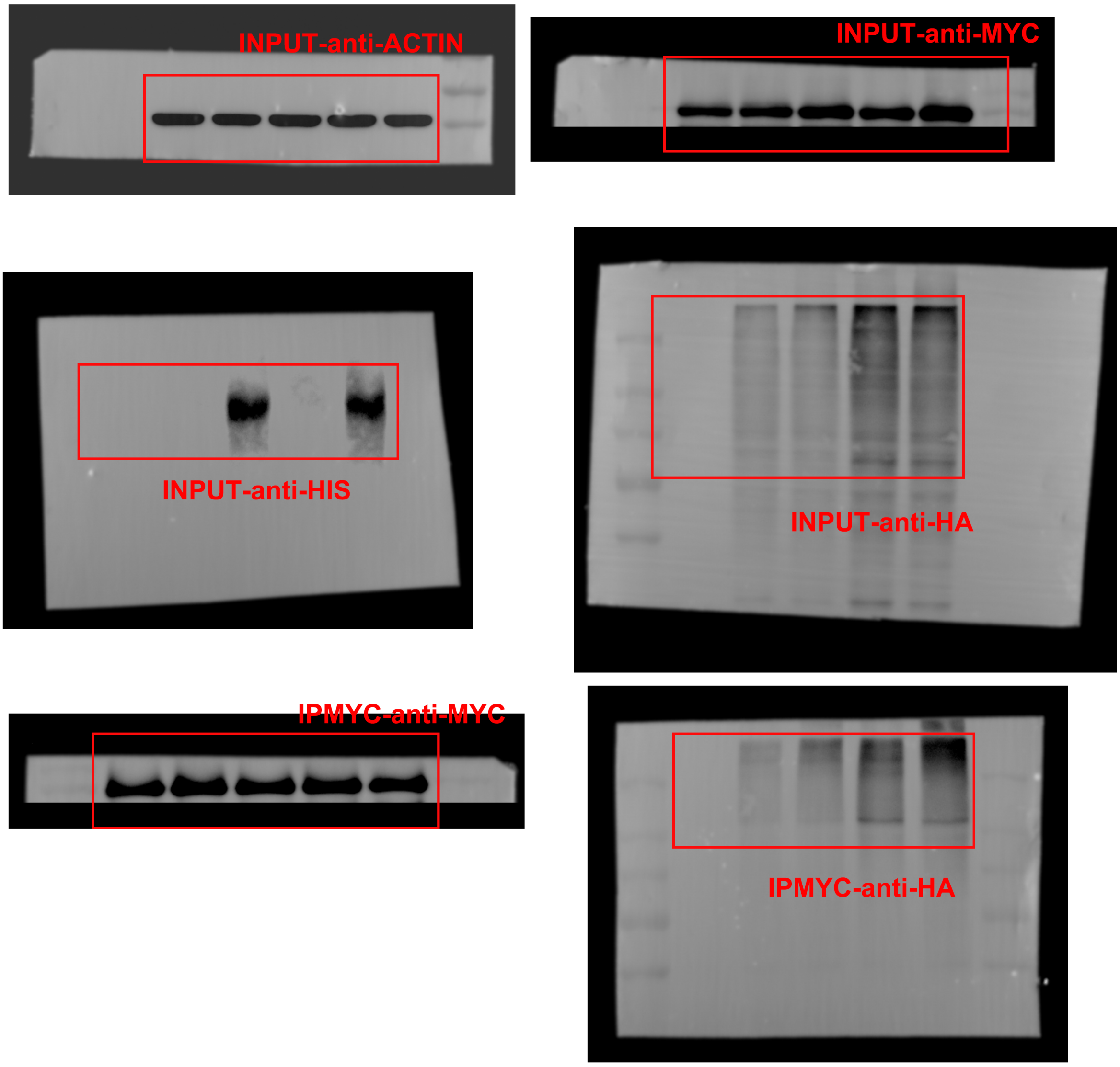

INPUT-anti-ACTIN
INPUT-anti-MYC
INPUT-anti-HIS
INPUT-anti-HA
IPMYC-anti-MYC
IPMYC-anti-HA

Figure 7H

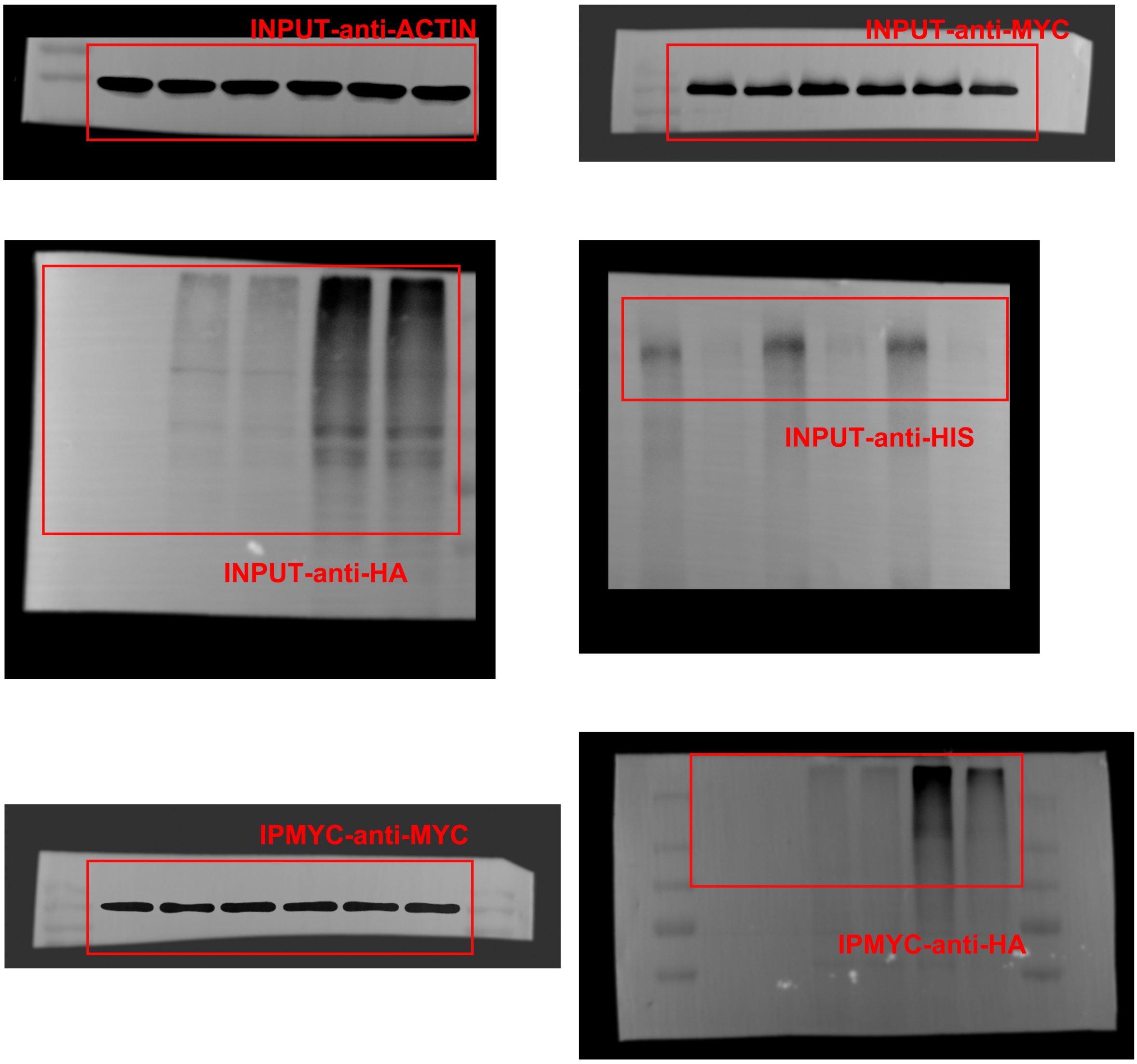
INPUT-anti-ACTIN
INPUT-anti-MYC
INPUT-anti-HA
INPUT-anti-HIS
IPMYC-anti-MYC
IPMYC-anti-HA

Figure 8A

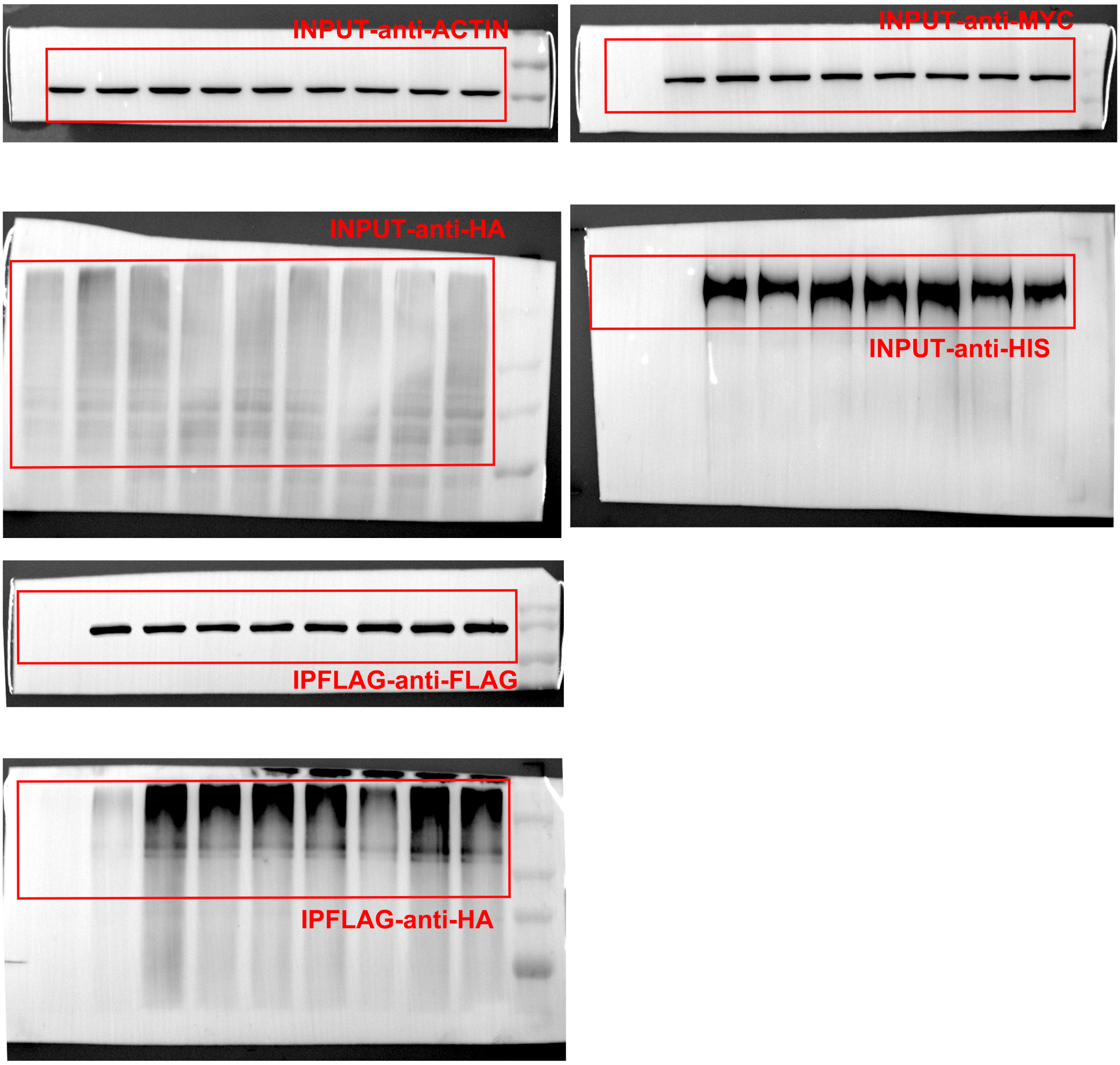
INPUT-anti-ACTIN
INPUT-anti-MYC
INPUT-anti-HA
INPUT-anti-HIS
IPFLAG-anti-FLAG
IPFLAG-anti-HA

Figure 8B

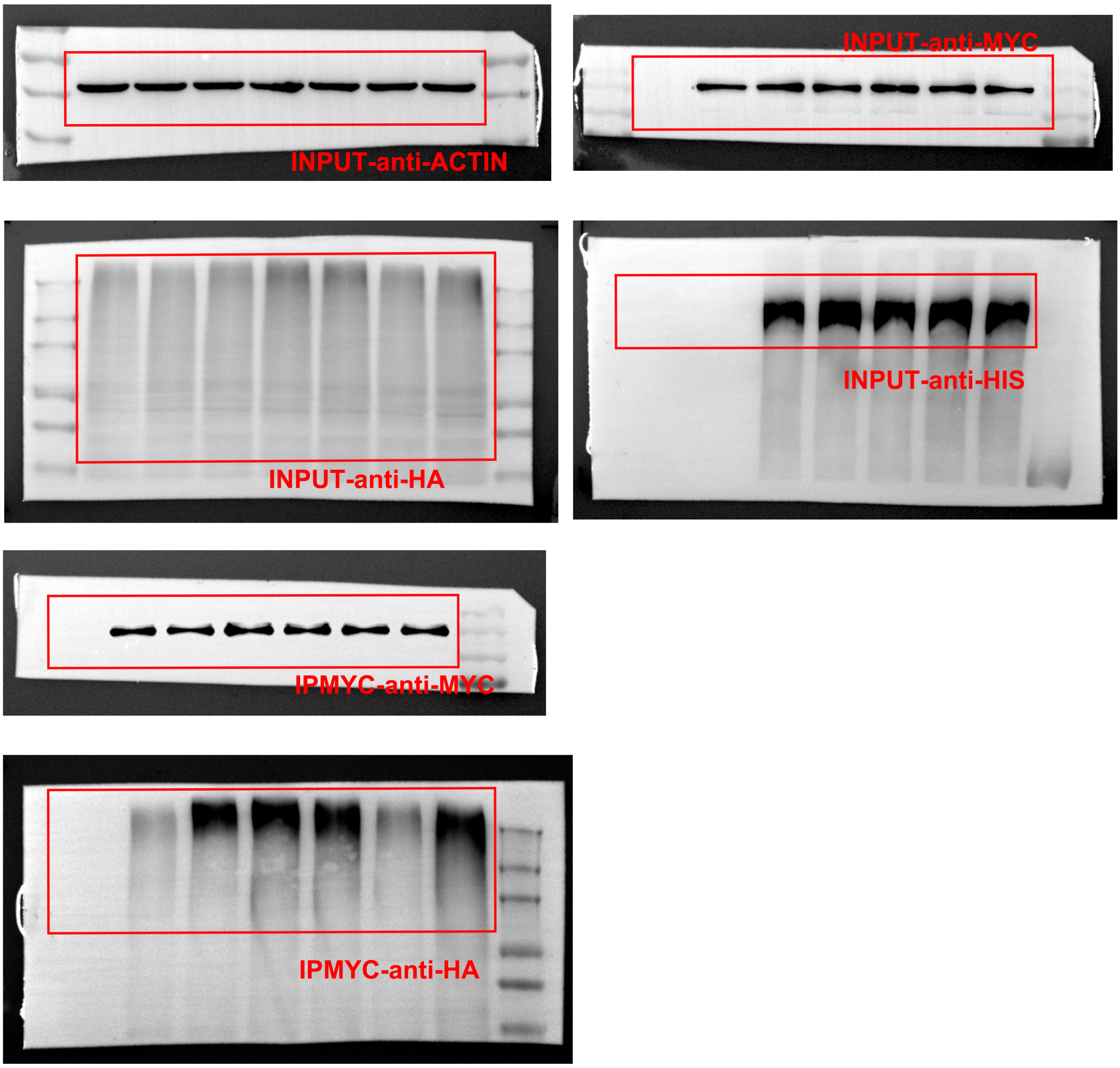
INPUT-anti-ACTIN
INPUT-anti-MYC
INPUT-anti-HA
INPUT-anti-HIS
IPMYC-anti-MYC
IPMYC-anti-HA

Figure 8C

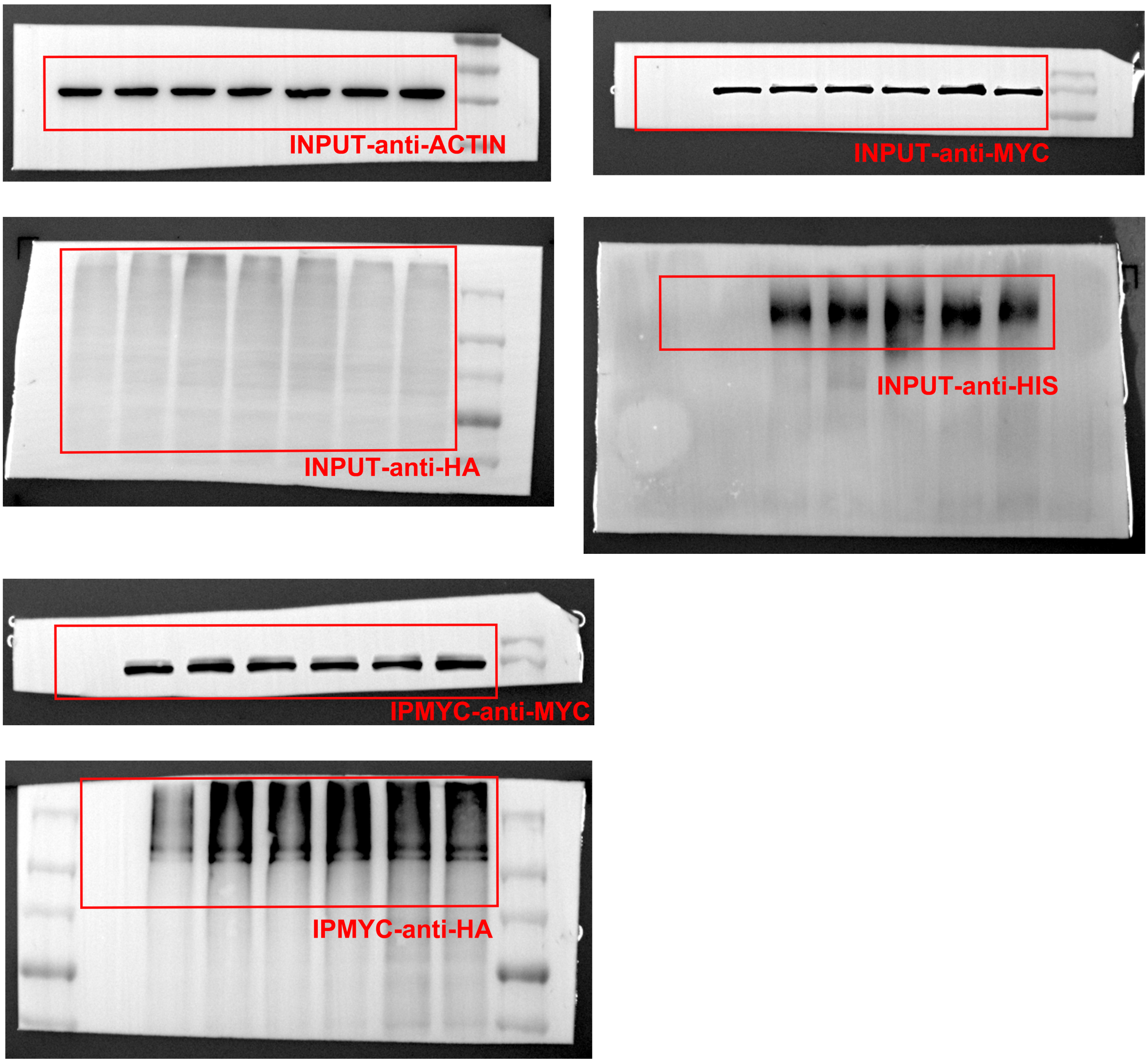
INPUT-anti-ACTIN
INPUT-anti-MYC
INPUT-anti-HA
INPUT-anti-HIS
IPMYC-anti-MYC
IPMYC-anti-HA

Figure 8D

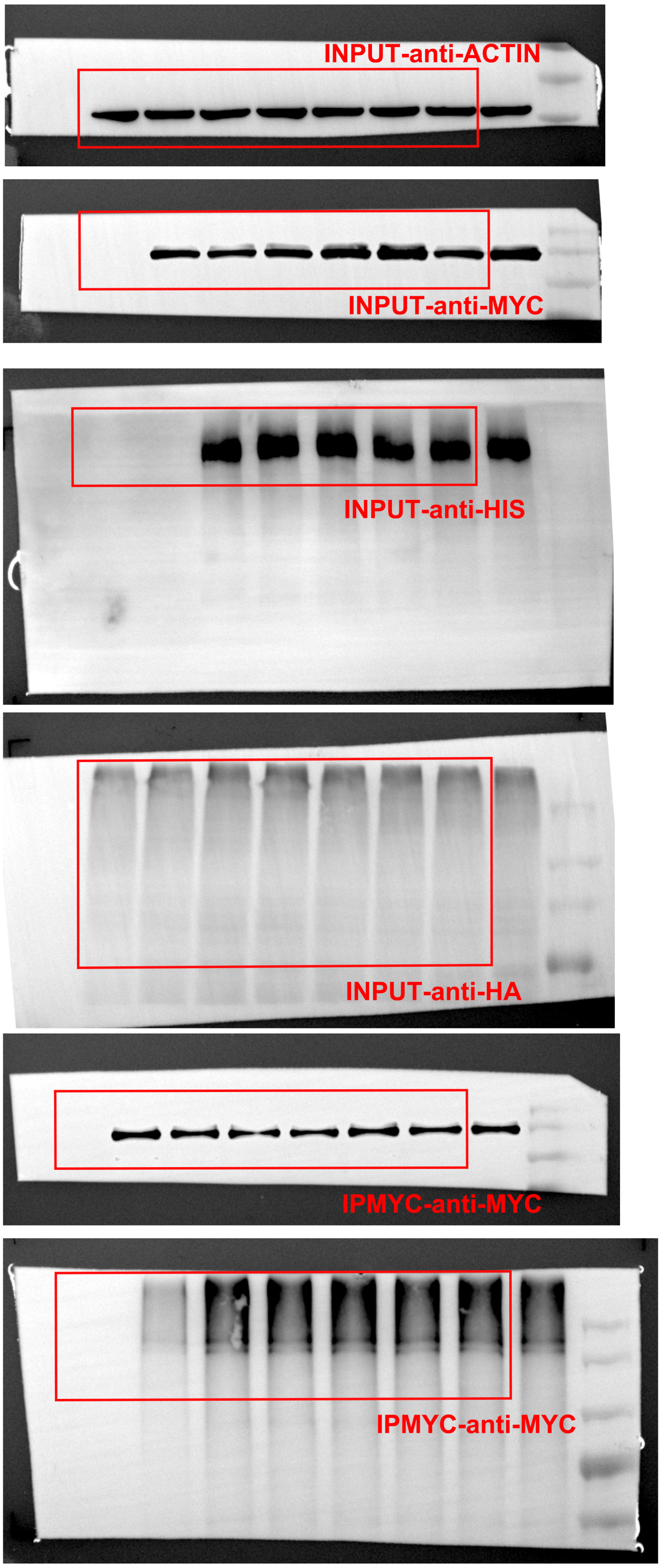
INPUT-anti-ACTIN
INPUT-anti-MYC
INPUT-anti-HIS
INPUT-anti-HA
IPMYC-anti-MYC
IPMYC-anti-MYC

Figure 8E

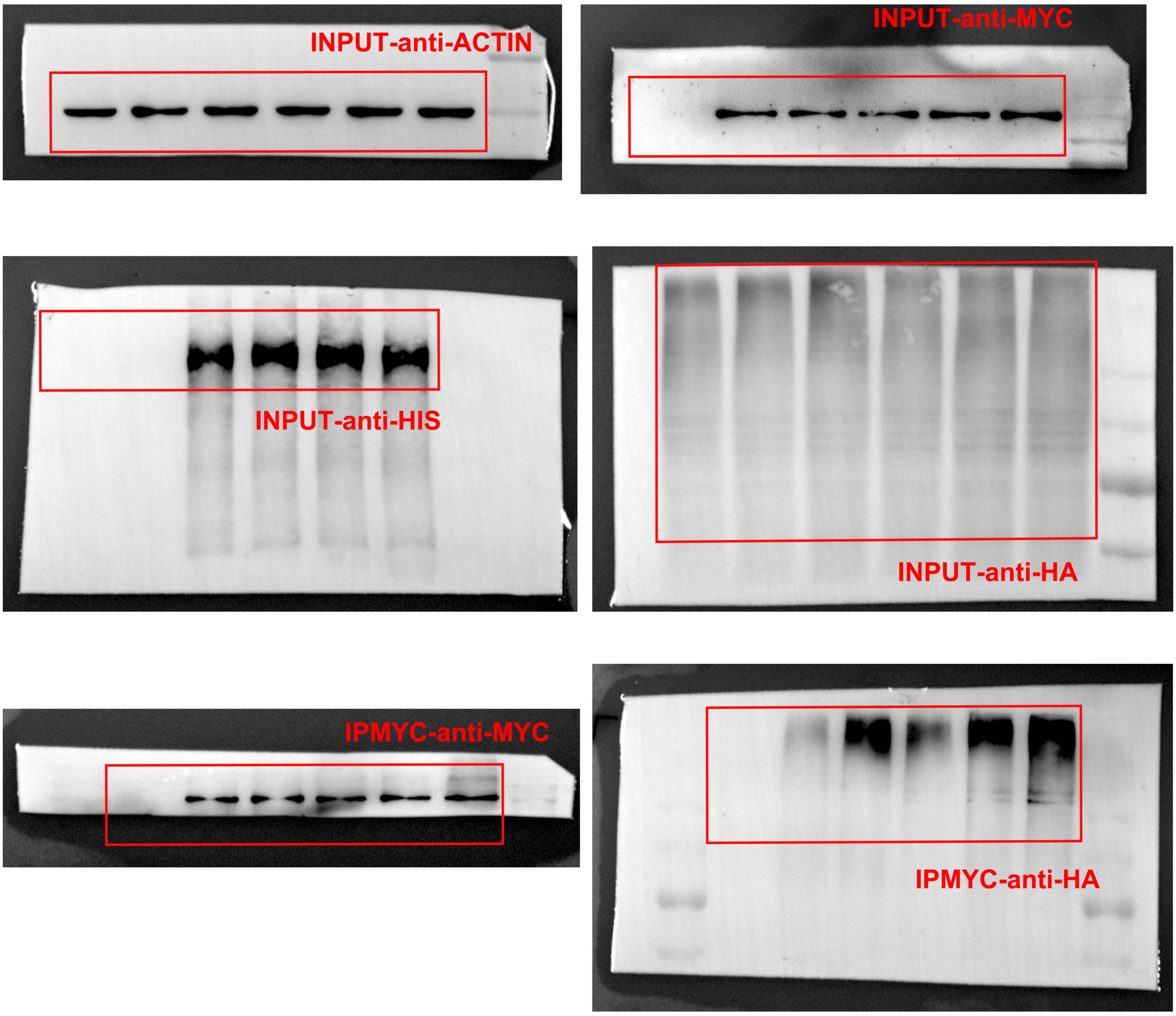
INPUT-anti-ACTIN
INPUT-anti-MYC
INPUT-anti-HIS
INPUT-anti-HA
IPMYC-anti-MYC
IPMYC-anti-HA

Figure 8F

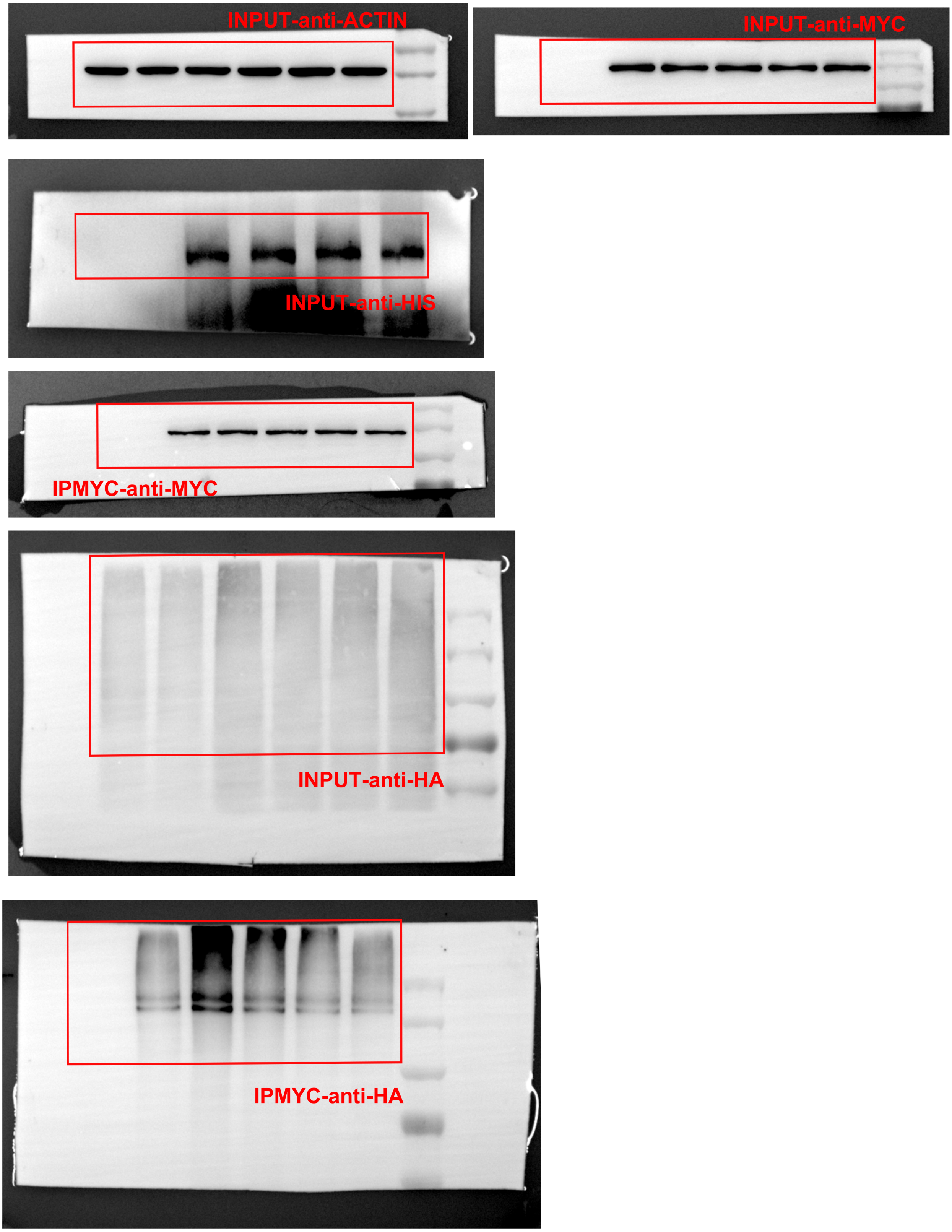
INPUT-anti-ACTIN
INPUT-anti-MYC
INPUT-anti-HIS
IPMYC-anti-MYC
INPUT-anti-HA
IPMYC-anti-HA

Figure 8H

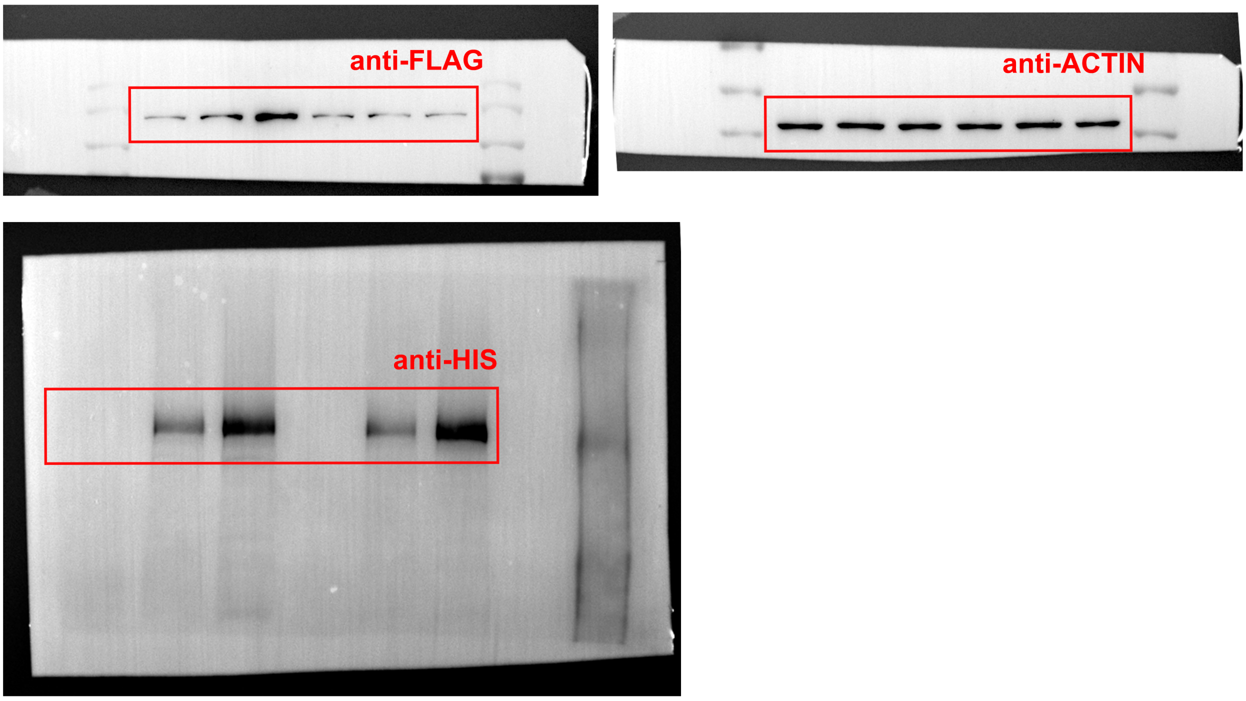

Figure 8I

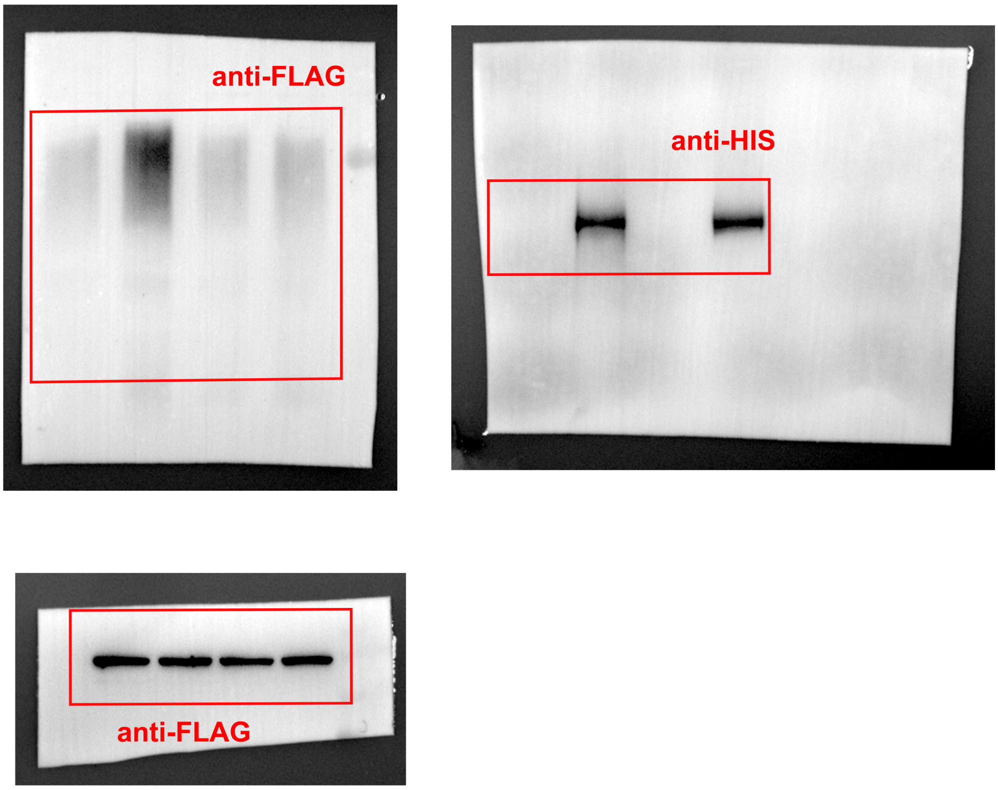

Figure 8J

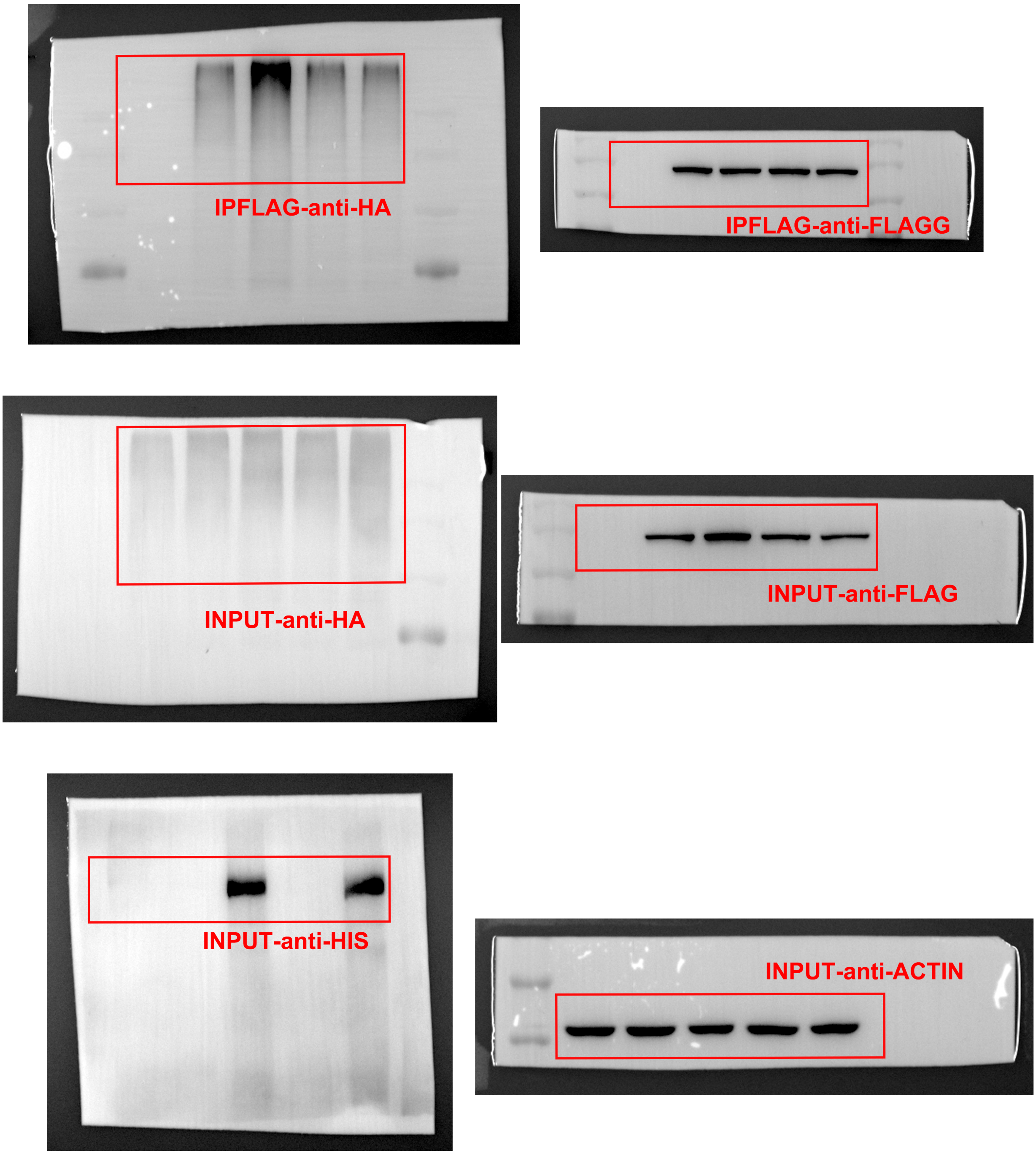
IPFLAG-anti-HA
IPFLAG-anti-FLAGG
INPUT-anti-HA
INPUT-anti-FLAG
INPUT-anti-HIS
INPUT-anti-ACTIN

Figure 8K

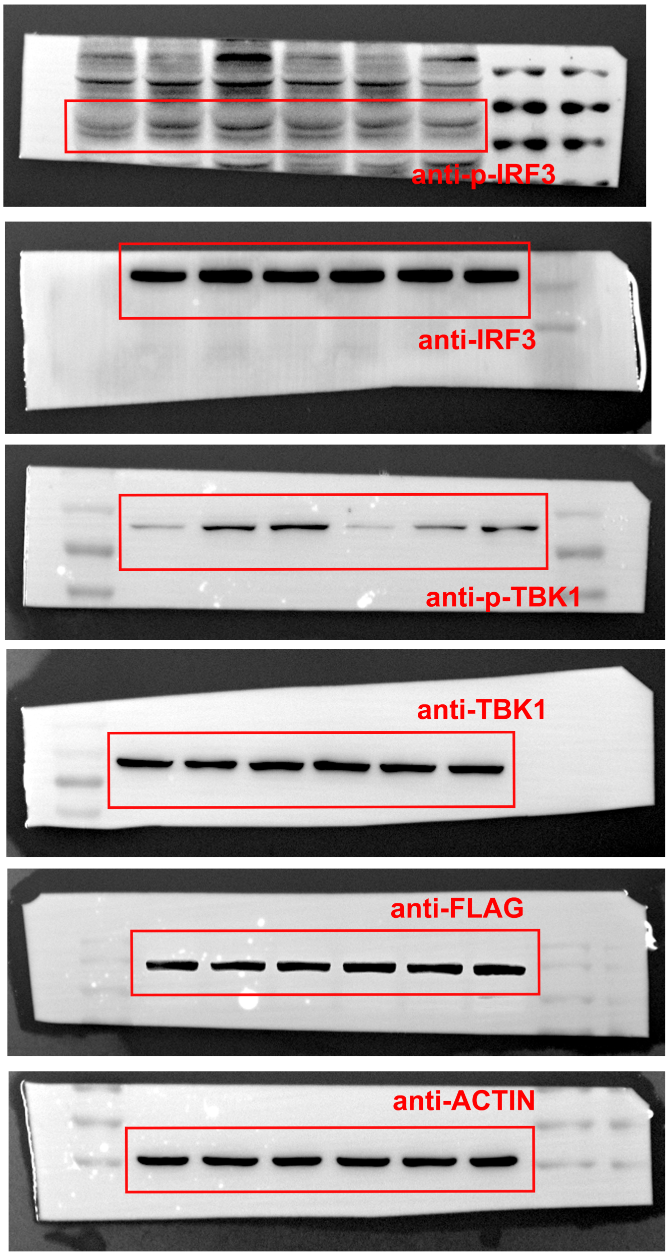
anti-p-IRF3
anti-IRF3
anti-p-TBK1
anti-TBK1
anti-FLAG
anti-ACTIN

Figure 8L

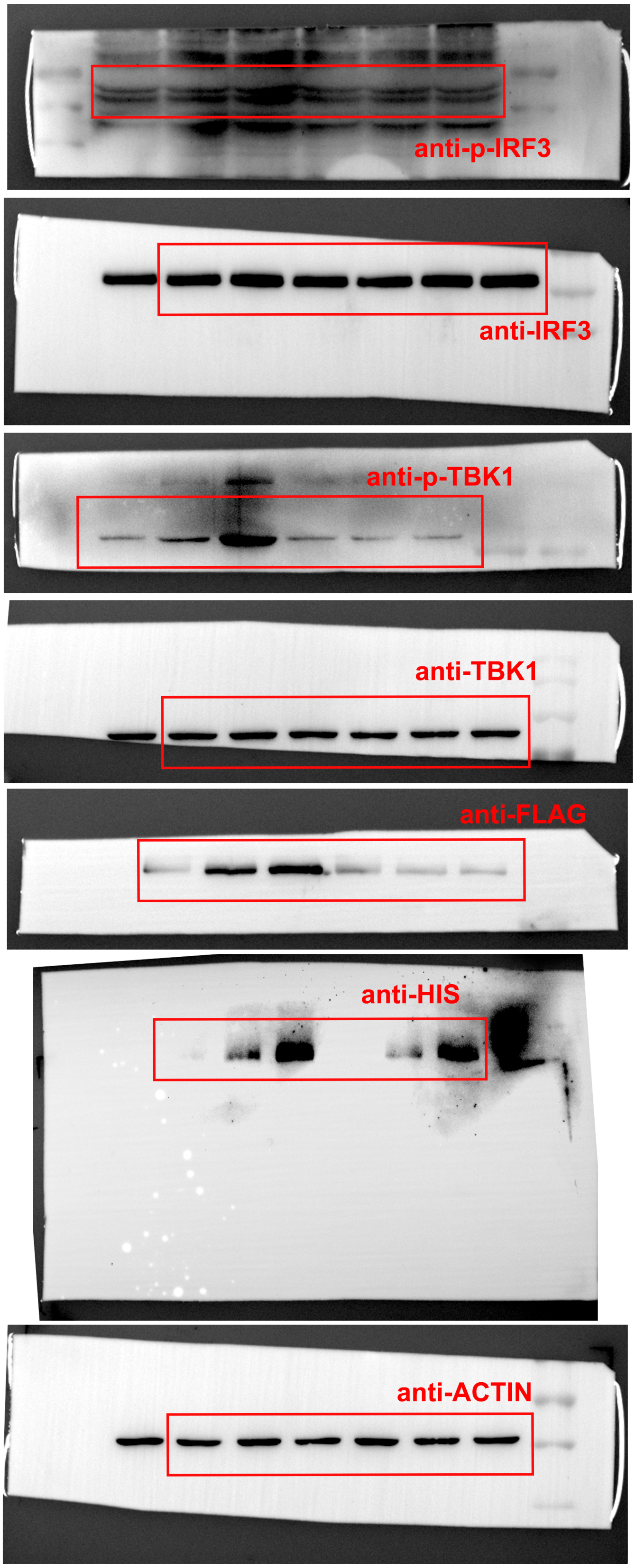
anti-p-IRF3
anti-IRF3
anti-p-TBK1
anti-TBK1
anti-FLAG
anti-HIS
anti-ACTIN

Figure 9C

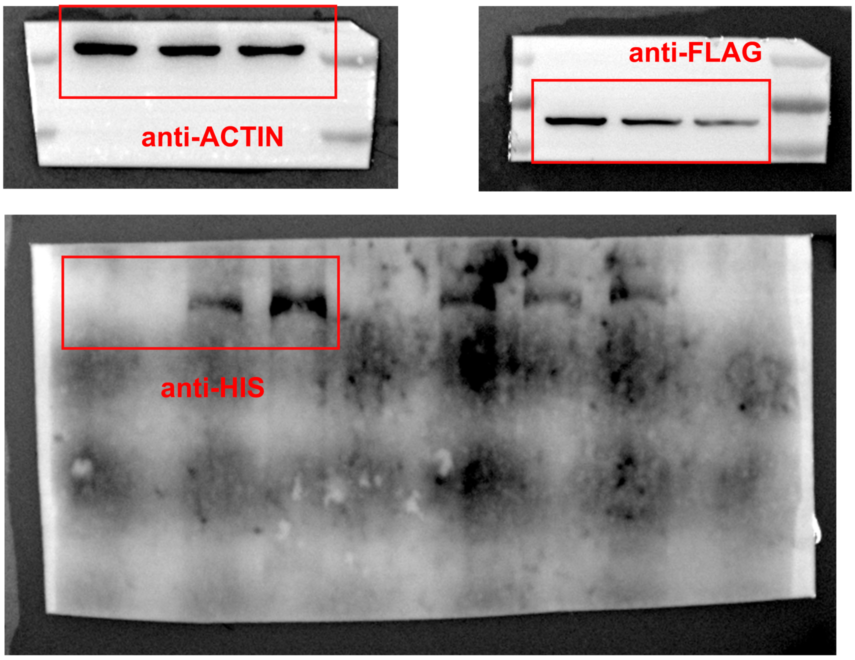

Figure 9D-left

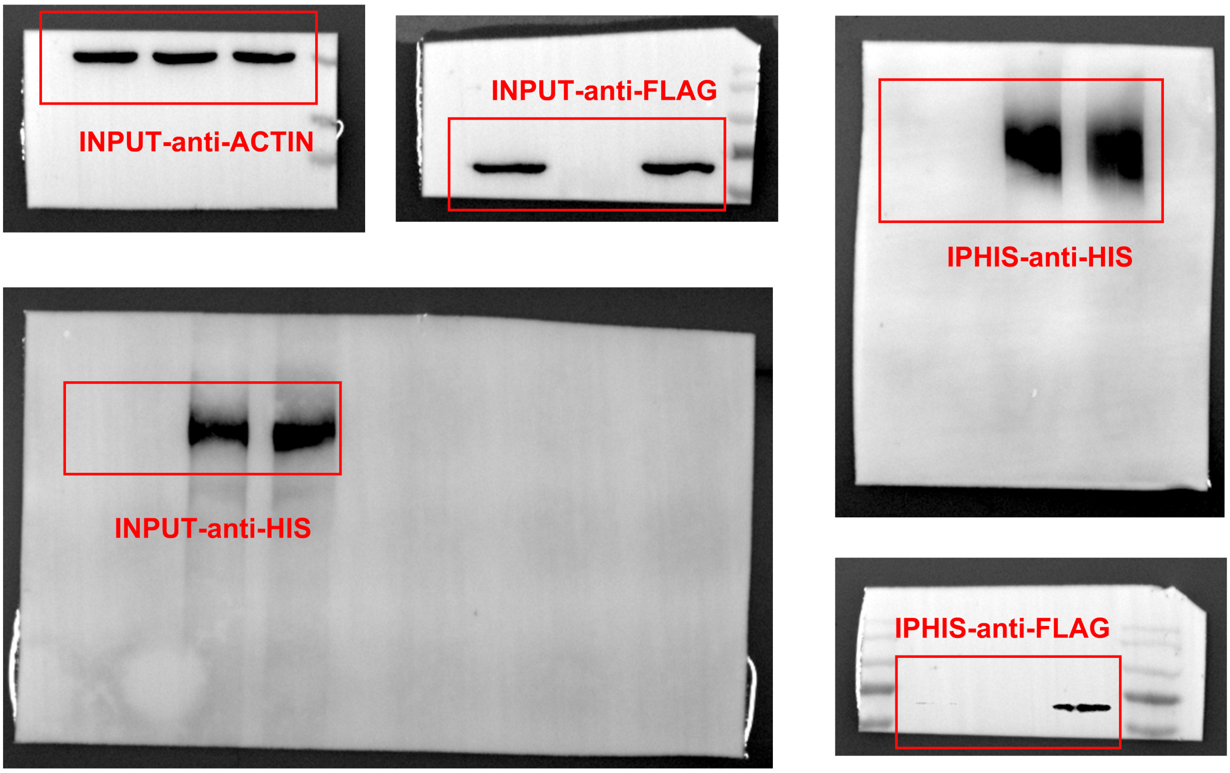

Figure 9D-right

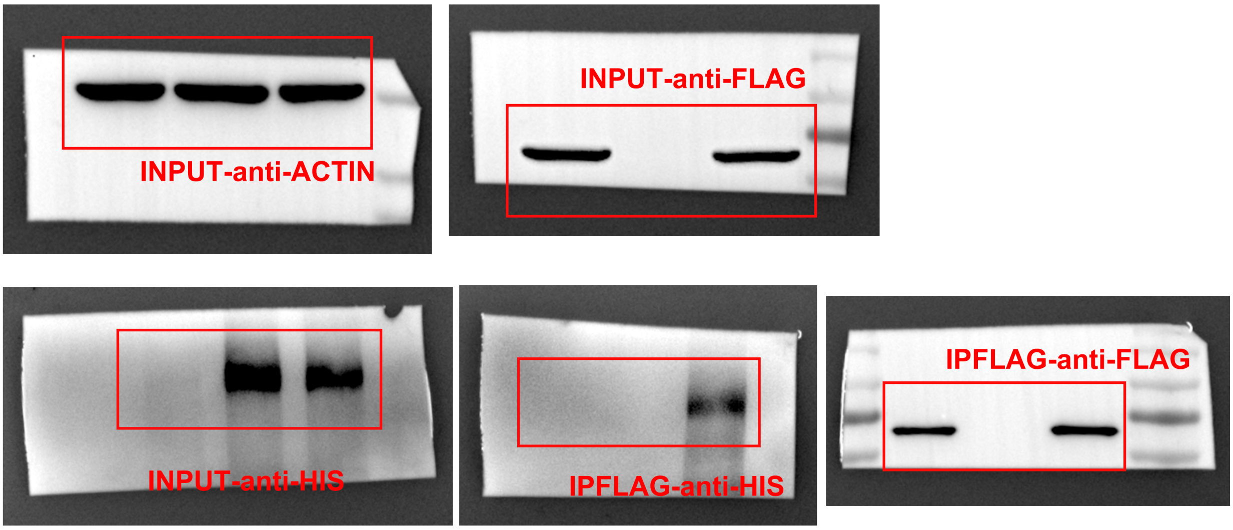

Figure 9E-left

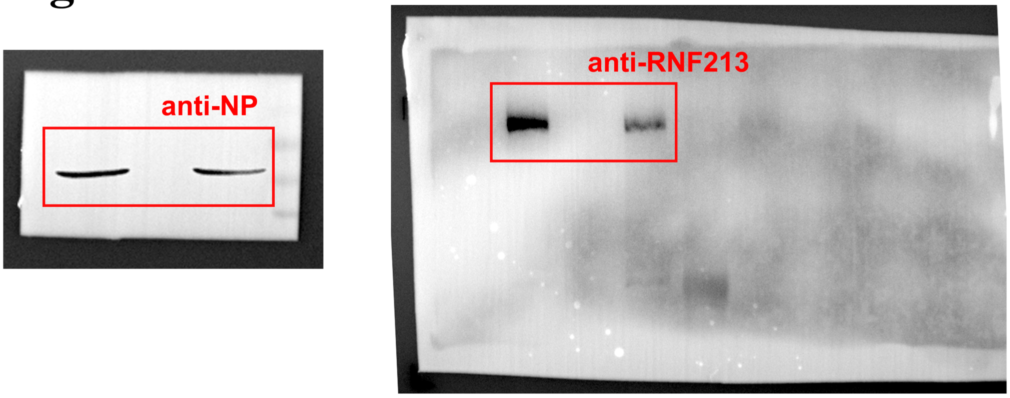

Figure 9E-Right

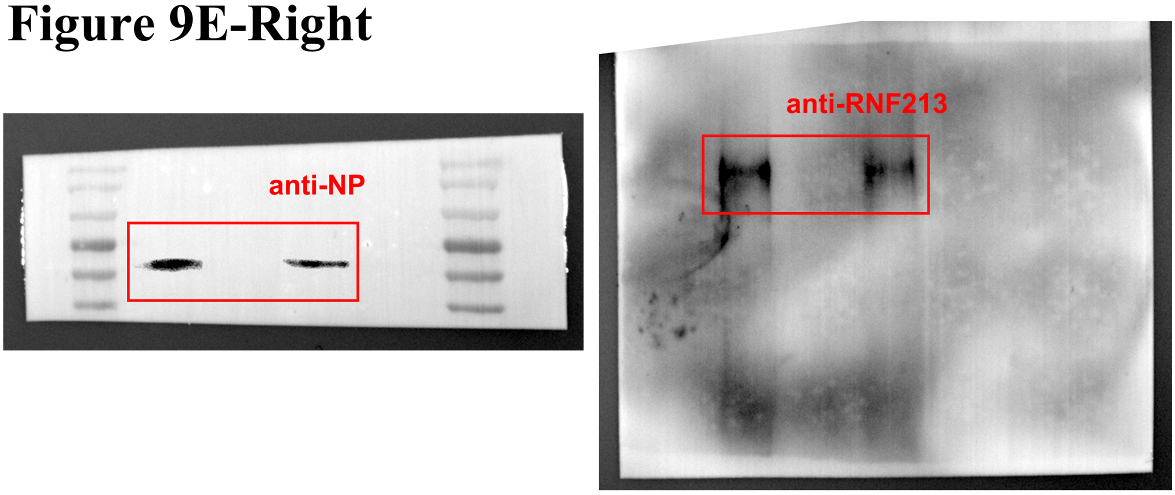

Figure 9F

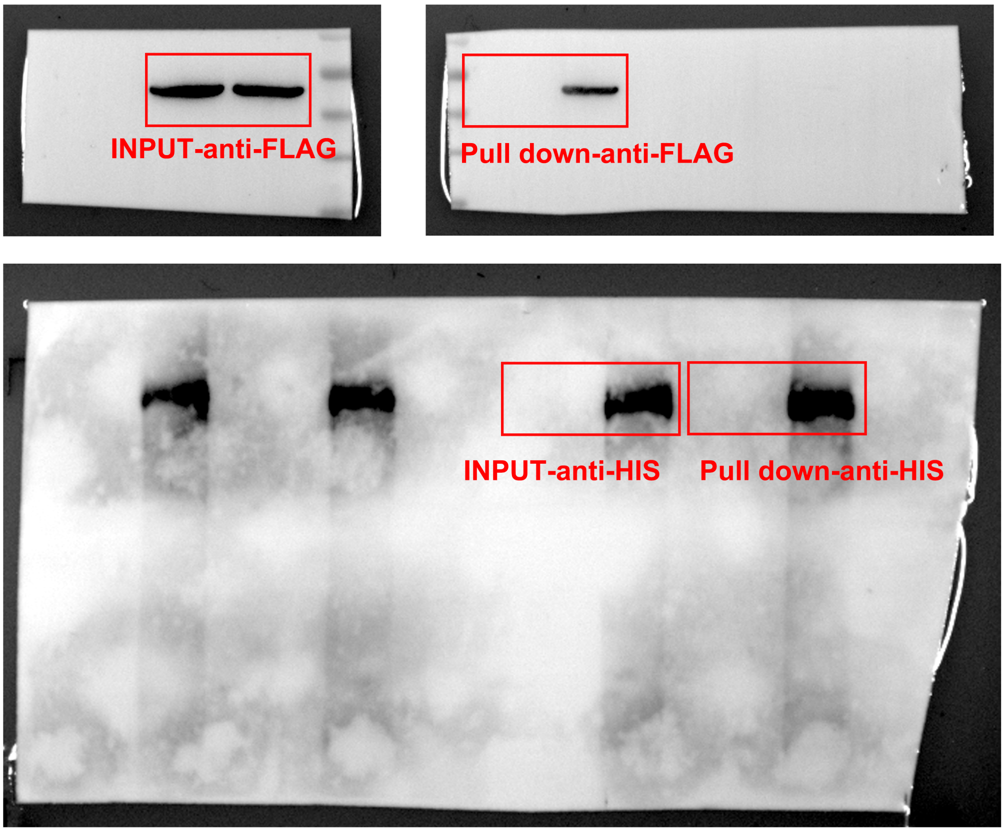

Figure 9G

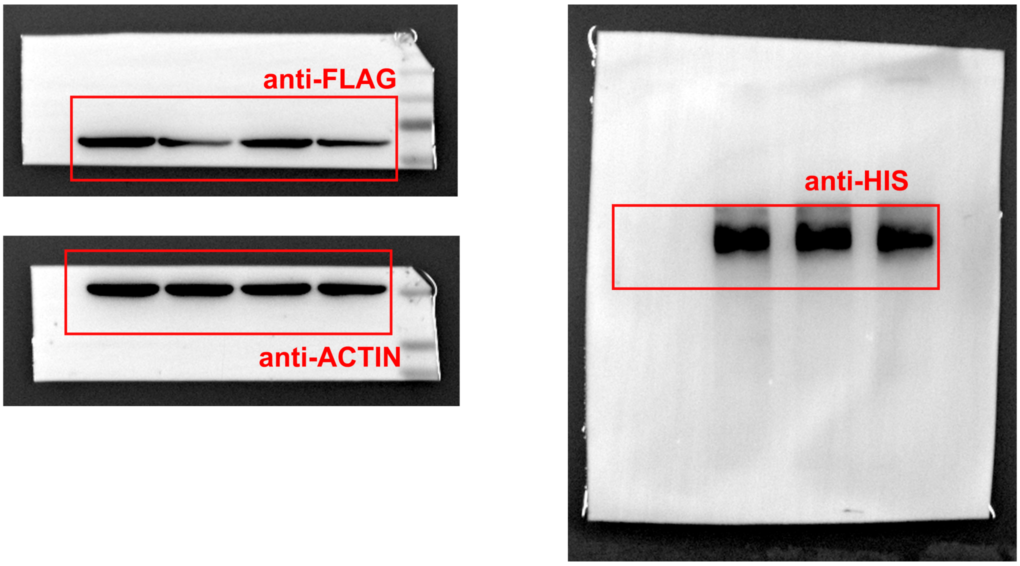

Figure 9H-left

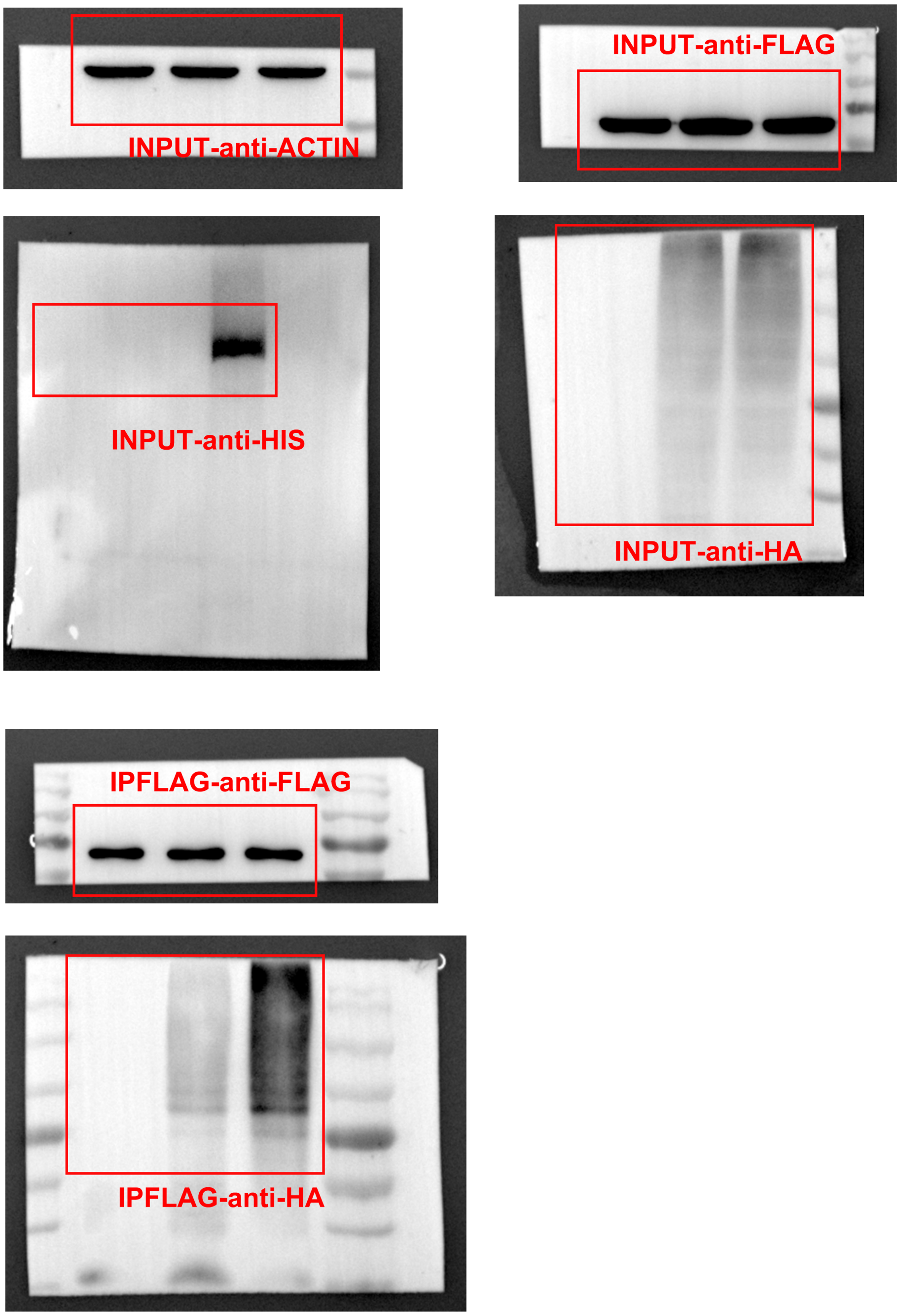

Figure 9H-right

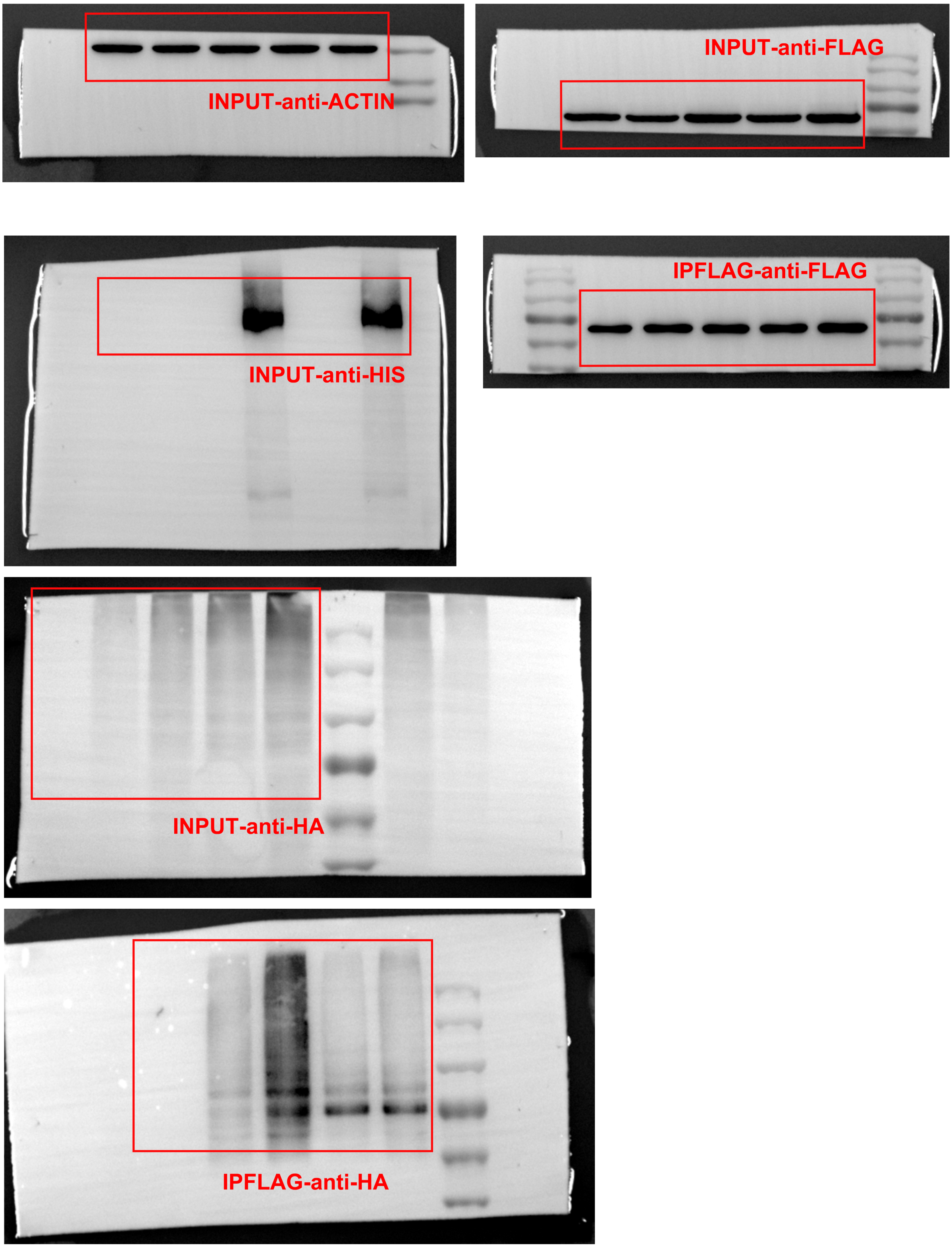

Figure S2

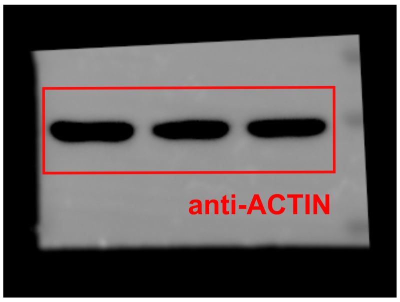

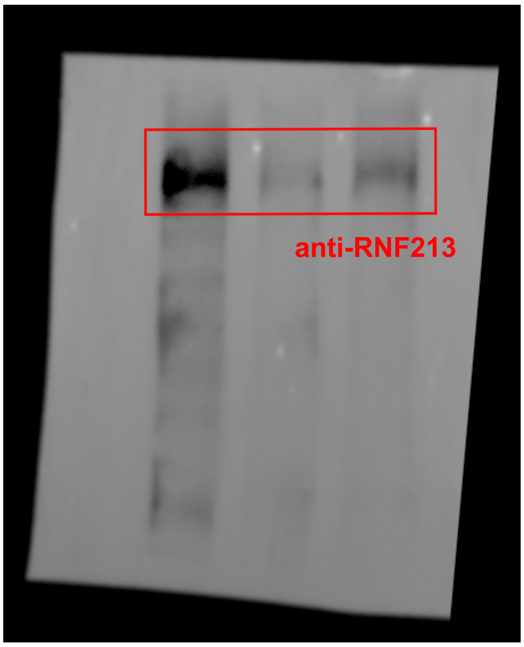

Figure S4

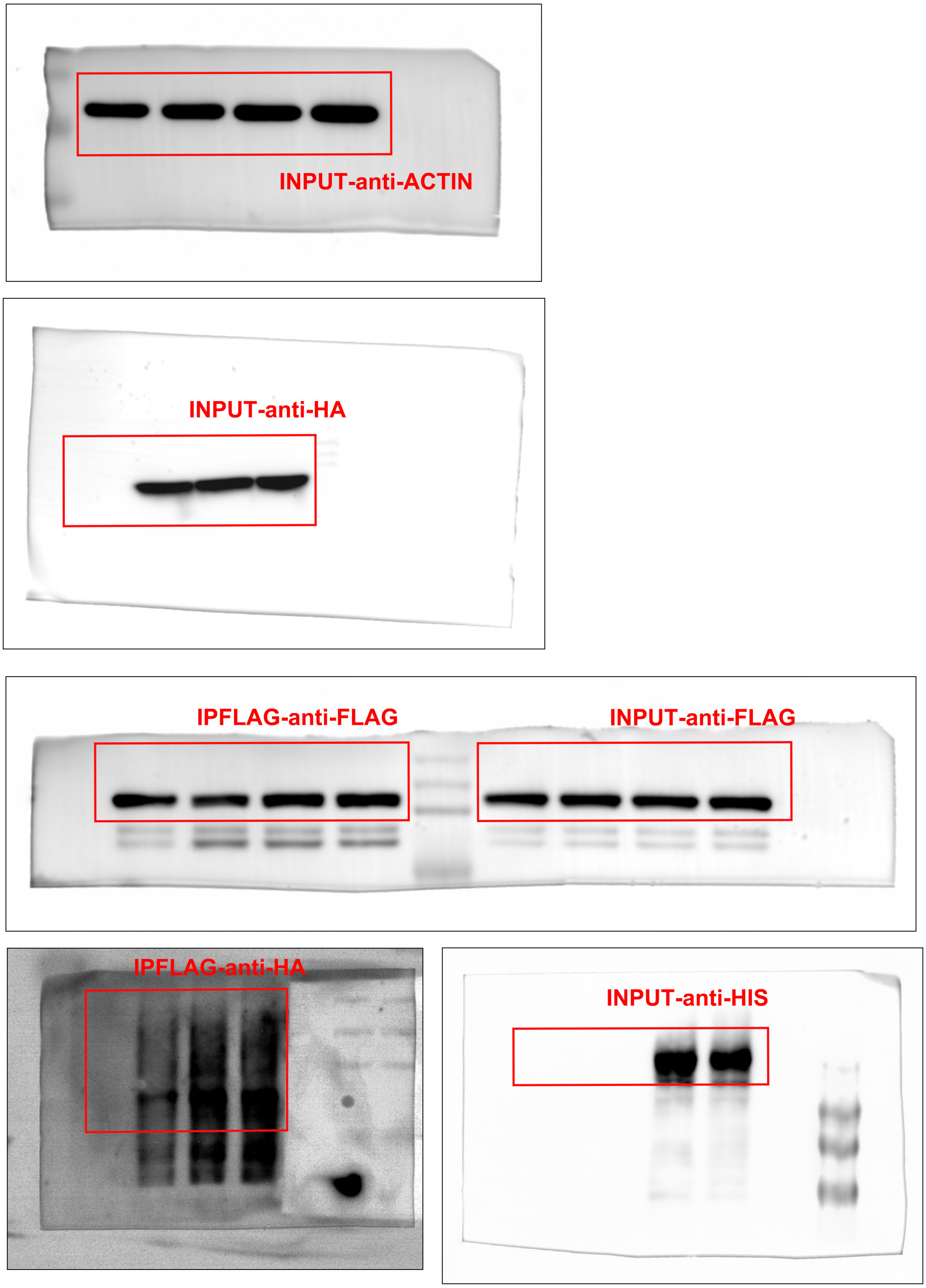

INPUT-anti-ACTIN
INPUT-anti-HA
IPFLAG-anti-FLAG
INPUT-anti-FLAG
IPFLAG-anti-HA
INPUT-anti-HIS
