# Supplementary material for: RNF213 Is an Interferon‐Stimulated Gene That Targets Influenza A Virus NP and Activates MDA5 to Restrict Infection
Source: Adv Sci (Weinh). 2026 Mar 18;13(31):e11012. doi: 10.1002/advs.202511012 (PMC13588011; doi:10.1002/advs.202511012)
Supplement: Supplementary file 4 [file ADVS-13-e11012-s006.docx]

**Table S3.** **Potential ubiquitination sites within MDA5.**
